# DNA hypomethylation leads to cGAS‐induced autoinflammation in the epidermis

**DOI:** 10.15252/embj.2021108234

**Published:** 2021-09-29

**Authors:** Mirjam A Beck, Heinz Fischer, Lisa M Grabner, Tamara Groffics, Mircea Winter, Simone Tangermann, Tina Meischel, Barbara Zaussinger‐Haas, Patrick Wagner, Carina Fischer, Christina Folie, Julia Arand, Christian Schöfer, Bernard Ramsahoye, Sabine Lagger, Georg Machat, Gregor Eisenwort, Stephanie Schneider, Alexandra Podhornik, Michael Kothmayer, Ursula Reichart, Martin Glösmann, Ido Tamir, Michael Mildner, Raheleh Sheibani‐Tezerji, Lukas Kenner, Peter Petzelbauer, Gerda Egger, Maria Sibilia, Andrea Ablasser, Christian Seiser

**Affiliations:** ^1^ Division of Cell and Developmental Biology Center for Anatomy and Cell Biology Medical University of Vienna Vienna Austria; ^2^ Unit of Laboratory Animal Pathology University of Veterinary Medicine Vienna Austria; ^3^ Spinal Cord Injury and Tissue Regeneration Center Salzburg (SCI‐TReCS) Salzburg Austria; ^4^ MRC Institute of Genetics & Molecular Medicine Centre for Genomic & Experimental Medicine University of Edinburgh Edinburgh UK; ^5^ Institute of Immunology Medical University of Vienna Vienna Austria; ^6^ VetCORE ‐ Facility for Research University of Veterinary Medicine Vienna Vienna Austria; ^7^ Campus Science Support Facilities GmbH Vienna Austria; ^8^ Research Division of Biology and Pathobiology of the Skin Medical University of Vienna Vienna Austria; ^9^ Ludwig Boltzmann Institute Applied Diagnostics Vienna Austria; ^10^ Christian Doppler Laboratory for Applied Metabolomics Vienna Austria; ^11^ Department of Pathology Medical University of Vienna Vienna Austria; ^12^ CBmed GmbH ‐ Center for Biomarker Research in Medicine Graz Austria; ^13^ Skin and Endothelium Research Division Department of Dermatology Medical University of Vienna Vienna Austria; ^14^ Department of Medicine I Comprehensive Cancer Center Institute of Cancer Research Medical University of Vienna) Vienna Austria; ^15^ Global Health Institute Swiss Federal Institute of Technology Lausanne (EPFL) Lausanne Switzerland; ^16^ Present address: Max Perutz Labs Vienna BioCenter Vienna Austria; ^17^ Present address: Department of Microbiology and Immunology The University of Melbourne at The Peter Doherty Institute for Infection and Immunity Melbourne Vic. Australia; ^18^ Present address: Department of Hematology and Hemostaseology Clinic for Internal Medicine I Medical University of Vienna Vienna Austria; ^19^ Present address: Gene Center ‐ Max von Pettenkofer‐Institute of Virology Ludwig‐Maximilians‐Universität München München Germany

**Keywords:** autoinflammation, cytosolic DNA, DNA methylation, epigenetics, innate immune system, Chromatin, Transcription & Genomics, Immunology

## Abstract

DNA methylation is a fundamental epigenetic modification, important across biological processes. The maintenance methyltransferase DNMT1 is essential for lineage differentiation during development, but its functions in tissue homeostasis are incompletely understood. We show that epidermis‐specific DNMT1 deletion severely disrupts epidermal structure and homeostasis, initiating a massive innate immune response and infiltration of immune cells. Mechanistically, DNA hypomethylation in keratinocytes triggered transposon derepression, mitotic defects, and formation of micronuclei. DNA release into the cytosol of DNMT1‐deficient keratinocytes activated signaling through cGAS and STING, thus triggering inflammation. Our findings show that disruption of a key epigenetic mark directly impacts immune and tissue homeostasis, and potentially impacts our understanding of autoinflammatory diseases and cancer immunotherapy.

## Graphical abstract

Deletion of maintenance methyltransferase DNMT1 in keratinocytes causes formation of micronuclei, whose rupture and cytosolic DNA release activates inflammatory signalling via cGAS/STING.

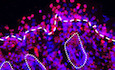

## Introduction

As the outermost organ of the body, the skin is continuously exposed to pathogens and plays a critical role in the defense against the environment and infectious agents. The most superficial layer of the skin, the epidermis, functions as a critical barrier against viruses, bacteria, and fungi (Alonso & Fuchs, 2003; Fuchs & Horsley, 2008). Various immune cell types reside in the skin or are recruited to maintain skin homeostasis upon exposure to pathogens, including innate immune cells (Kabashima *et al*, 2019; Nguyen & Soulika, 2019). Pathogen‐derived nucleic acids are an important signal for innate immunity and detected through specific RNA‐ and DNA‐sensing mechanisms. These mammalian systems distinguish self from non‐self through attributes including intracellular localization, secondary structure, post‐transcriptional modifications, and abundance of specific nucleic acids (Roers *et al*, 2016; Schlee & Hartmann, 2016; Bartok & Hartmann, 2020). A major mechanism for cytosolic DNA detection is the cGAS/STING pathway (Ablasser & Hur, 2020). The cGAS protein is a central receptor for cytosolic double‐stranded DNA that mediates the upregulation of type I interferons and other inflammatory cytokines and chemokines. This occurs via STING activation by the cyclic dinucleotide cGAMP and results in phosphorylation and nuclear translocation of IRF3. The cGAS/STING pathway also plays an important role in the recognition of malignant cells by the immune system (Flood *et al*, 2019; Hoong *et al*, 2020; Kwon & Bakhoum, 2020; Yum *et al*, 2020).

The epidermis is a key context for the operation of the innate immune system and is a self‐renewing tissue that requires precise spatiotemporal control of gene expression (Alonso & Fuchs, 2003; Mikkola, 2007). This is achieved through a well‐orchestrated interplay between transcription factors and epigenetic regulators mediating the regulation of gene expression (Miroshnikova *et al*, 2019). Heritable CpG methylation is probably the best‐studied epigenetic mechanism in mammalian organisms. DNA methylation is mediated by DNA methyltransferases (DNMTs) and plays an important role during development by repressing gamete‐specific genes or entire chromosomes such as mammalian X chromosome inactivation (Schubeler, 2015; Ambrosi *et al*, 2017). DNMTs are also involved in the control of allele‐specific gene expression and silencing of repetitive elements, thereby maintaining genome integrity (Ambrosi *et al*, 2017). Among the five known members of the DNMT family, DNMT1, DNMT3A, and DNMT3B play an important role during mouse development (Smith & Meissner, 2013). DNMT3A and DNMT3B are known as the *de novo* DNA methyltransferases showing high activity toward unmethylated DNA (reviewed in Espada & Esteller, 2010; Karemaker & Baubec, 2020). DNMT1 has substrate specificity for hemimethylated DNA, but can also transfer the methyl group to unmethylated DNA templates and is therefore considered as the maintenance methyltransferase (reviewed in Mohan and Chaillet (2013) and Karemaker and Baubec (2020)). Of note, DNMTs are relevant targets for small molecule inhibitors in cancer treatment and specific DNMT inhibitors are FDA‐approved for the treatment of acute myeloid leukemia (Jones *et al*, 2016).

Several severe chronic inflammatory skin diseases, including psoriasis and atopic dermatitis, show abnormal DNA methylation at genes regulating epidermal differentiation and innate immune response in the epidermis of patients as well as in their T cells (Gudjonsson & Krueger, 2012; Han *et al*, 2012; Rodriguez *et al*, 2014). In these diseases, the defective DNA methylation is accompanied by disruptions in both keratinocyte differentiation and the barrier function of the skin. Several studies suggest that the appearance of various inflammatory skin diseases and autoimmune disorders, as well as skin cancer, stems from altered epigenetic mechanisms (Javierre *et al*, 2012).

A function for DNA methylation in epidermal self‐renewal and differentiation was previously described using human *DNMT1* knockdown keratinocytes in immunodeficient mice (Sen *et al*, 2010). This study linked DNMT1 to the maintenance of progenitor function in the epidermis and its deletion resulted in premature differentiation and tissue loss. In another study, epidermal DNMT1 disruption in aging mice resulted in progressive alopecia due to decreased stem cell activation (Li *et al*, 2012). However, variable and incomplete deletion of *Dnmt1* was seen in this conditional mouse model. We therefore used a different approach, epidermal DNMT1 ablation in immune competent mice using the Keratin 5‐Cre (K5‐Cre) system (Winter *et al*, 2013), to examine the effects of this loss on the skin.

Here, we show that DNMT1 ablation triggers a strong pathological innate immune response in the skin, resulting in immune cell infiltration and subsequent destruction of normal skin architecture. DNA hypomethylation in keratinocytes led to formation of micronuclei and accumulation of cytosolic DNA due to mitotic defects and genomic instability. This in turn caused cGAS‐dependent activation of the innate immune system inducing an autoinflammatory phenotype. Intriguingly, additional deletion of cGAS in *Dnmt1*‐deficient mice significantly diminished the innate immune response and significantly ameliorated the skin destruction phenotype. Our study reveals a novel role of DNA methylation in restraint of the innate immune response to self‐nucleic acids.

## Results

### DNA hypomethylation results in disruption of epidermal homeostasis

**Figure 1:**
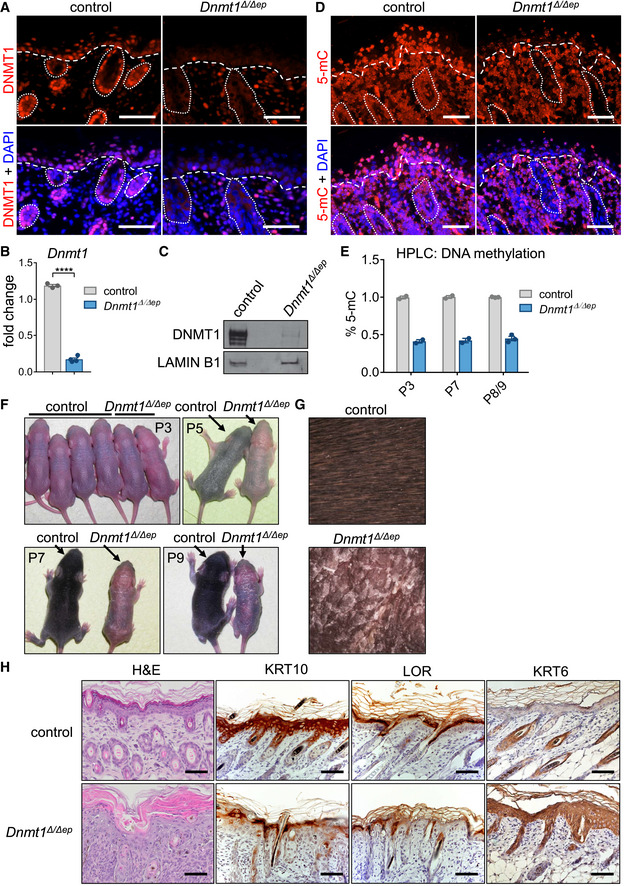
Epidermal DNA hypomethylation results in a severe skin phenotype AImmunofluorescence labeling of wild‐type mouse dorsal skin samples (*Dnmt1^f^* ^/^ *^f^*, skin from P3) shows expression of DNMT1 throughout epidermal layers whereas keratinocyte‐specific knockout (*Dnmt1^Δ^* ^/^ *^Δep^*) results in the almost absence of a specific staining in the epidermis and in hair follicles. Dashed lines indicate the dermal–epidermal border, and dotted lines indicate hair follicles.B, CDeletion of DNMT1 was also confirmed in isolated epidermis by analysis of RNA expression ((B), and data are mean ± SEM, two‐tailed *t*‐test, ****P ≤ 0.001, *n* = 3‐4 mice) and Western blotting (C).DKnockout of *Dnmt1* results in lower levels of DNA methylation in the epidermis and in hair follicles as determined by anti 5‐mC labeling of P3 skin. Dashed lines indicate the dermal–epidermal border, and dotted lines indicate hair follicles.EHPLC analysis of cytosine methylation in the epidermis of *Dnmt1^Δ^* ^/^ *^Δep^* mice compared with control littermates at different time points after birth. Values are percentages of methylated CpGs compared with total number of CpGs in the genome. P3: *n* = 2, P7: *n* = 2, P8/9: *n* = 3. Data are shown as mean ± SEM.F, GImages of control (*Dnmt1^f^* ^/^ *^f^*, *Dnmt1^f^* ^/+^) and *Dnmt1^Δ^* ^/^ *^Δep^* littermates at P3, P5, P7, and P9. An enlargement of the skin surface is shown on the right (G).HHematoxylin and eosin (H&E) staining, immunolabeling for KERATIN 10 (KRT10), LORICRIN (LOR), and KERATIN 6 (KRT6) expression of dorsal skin sections (P5) of control and *Dnmt1^Δ^* ^/^ *^Δep^* mice. Data information: Scale bars, 50 µm.

To study the role of DNA methylation in epidermal morphogenesis and homeostasis, we deleted *Dnmt1* in mouse epidermis using *Keratin 5*‐Cre (*K5‐Cre*), which is active from embryonic day 9.5 (E9.5) (Ramirez *et al*, 2004). Mice examined had the following genotypes: *K5‐Cre*+ *Dnmt1^f^* ^/^ *^f^* (*Dnmt1^Δ^* ^/^ *^Δep^*), *K5‐Cre* ^+^ *Dnmt1^f^* ^/+^ (*Dnmt1^Δ^* ^/+^ *^ep^*), *Dnmt1^f^* ^/^ *^f^* (control), and *Dnmt1^f^* ^/+^ (control). Immunolabeling of skin sections showed that Cre activation in *Dnmt1^Δ^* ^/^ *^Δep^* mice resulted in loss of DNMT1 expression in all layers of the epidermis and in hair follicles, as well as greatly reduced 5‐methylcytosine (5‐mC) in these cells (Fig 1A and D). Strong DNMT1 staining remained evident in non‐epithelial cells in the dermal layer (see also below). Analysis of mRNA and protein levels demonstrated that DNMT1 expression was strongly reduced in the epidermis of *Dnmt1^Δ^* ^/^ *^Δep^* mice (Fig 1B and C). In accordance with data obtained with DNMT1‐deficient embryonic stem cells (Li *et al*, 1992), deletion of *Dnmt1* in the epidermis resulted in a ˜ 60% reduction in genome‐wide DNA methylation in epidermal cells, as determined by quantitative HPLC (Fig 1E). Residual DNA methylation levels could be explained by DNMT1‐positive immune cells migrating into the epidermis as well as by compensating activities of DNMT3A and DNMT3B (Tsumura *et al*, 2006).

**Figure EV1:**
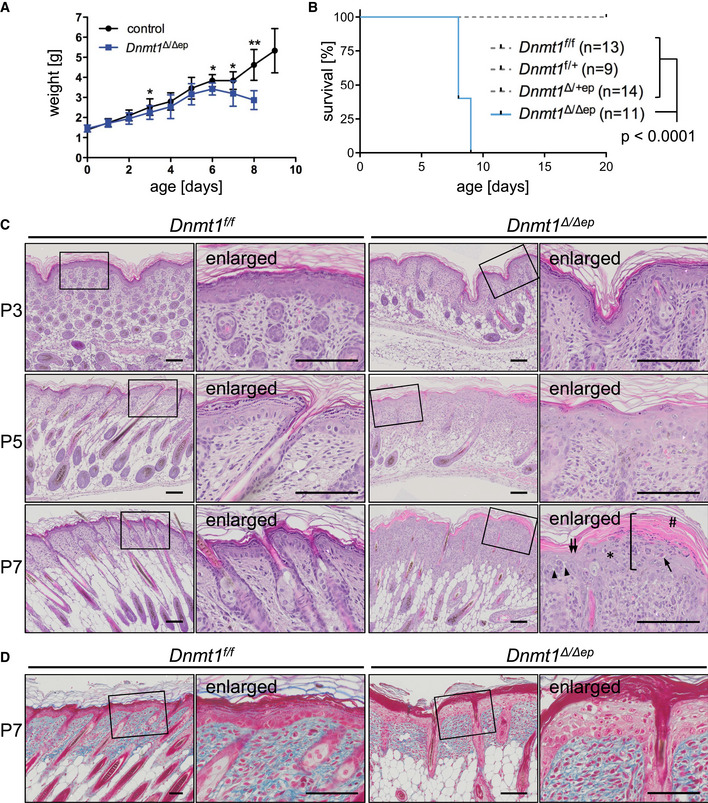
Keratinocyte‐specific deletion of *Dnmt1* in mice results in early postnatal lethality associated with severe dermatopathologic alterations Weight curve of control (*Dnmt1^f^* ^/^ *^f^*) and *Dnmt1^Δ^* ^/^ *^Δep^* mice from P0‐P10. Control: P0 *n* = 2; P1 *n* = 25; P2 *n* = 44; P3 *n* = 46; P4 *n* = 17; P5 *n* = 23; P6 *n* = 12; P7 *n* = 19; P8 *n* = 14; P9 *n* = 18. *Dnmt1^Δ^* ^/^ *^Δep^* mice: P0 *n* = 3; P1 *n* = 9; P2 *n* = 13; P3 *n* = 14; P4 *n* = 7; P5 *n* = 6; P6 *n* = 7; P7 *n* = 4; P8 *n* = 3; P9 all died. Data are mean ± SEM and compared using two‐tailed *t*‐test. *P ≤ 0.05, **P ≤ 0.01.Kaplan–Meier plot comparing control (*Dnmt1^f^* ^/^ *^f^ n* = 13, *Dnmt1^f^* ^/+^ *n* = 9, *Dnmt1^Δ^* ^/+^ *^ep^ n* = 14) and *Dnmt1^Δ^* ^/^ *^Δep^* (*n* = 11) mice using log‐rank test (Mantel–Cox). *P* < 0.0001.H&E staining of dorsal skin sections from control and *Dnmt1^Δ^* ^/^ *^Δep^* mice at P3, P5, and P7. Enlarged sections are indicated on the right. Dermatopathologic alterations found in *Dnmt1^Δ^* ^/^ *^Δep^* mice at P7 are indicated, such as acanthosis (bracket), hyperkeratosis (hashtag), loss of keratohyalin granules (double arrow), spongiosis (arrowheads), necrotic cells (arrow), and exocytosis (asterisk).Azan staining of dorsal skin section at P7. Enlarged sections are indicated on the right. Data information: Scale bars, 50 µm (D), 100 µm (C).

*Dnmt1^Δ^* ^/^ *^Δep^* mice were born at normal Mendelian ratios and initially displayed normal epidermal development, with regular barrier formation and unaffected differentiation (Appendix Fig S1A–C). However, from postnatal day 3 (P3) onward, *Dnmt1^Δ^* ^/^ *^Δep^* mice developed a severe skin phenotype characterized by dry, reddish, scaly, and atrophied skin as well as by alopecia (Figs 1F and G, and EV1C and D). At later stages (P7‐9), wounds and lesions appeared on mechanically challenged areas, such as the neck and the extremities (Fig 1F and G). Compared to control littermates, *Dnmt1^Δ^* ^/^ *^Δep^* mice gained less weight until P6, lost weight thereafter, and died between P8 and P9 (Fig EV1A and B). The full milk belly of those pups indicates that abandonment or starvation was not the cause of death.

Histological examination revealed that these phenotypes in *Dnmt1^Δ^* ^/^ *^Δep^* pups were underpinned by a prominent alteration in tissue architecture, characterized by dermal atrophy and massive infiltration of immune cells (Figs 1H and EV1C and D). The dermis showed mild to marked fibrosis, characterized by proliferating fibroblasts, increased numbers of fibrocytes and deposition of collagen, as determined by Azan staining (Fig EV1D). Within the epidermal compartment, we found acanthosis, hyperkeratosis, loss of keratohyalin granules, parakeratosis, and impaired keratinocyte differentiation, as shown by reduced levels of keratin 10 and loricrin (Figs 1H and EV1C).

The basal layer of the epidermis contains highly proliferative cells, which regularly exit the cell cycle and undergo terminal differentiation. Strikingly, cell proliferation was markedly enhanced in the epidermis of *Dnmt1^Δ^* ^/^ *^Δep^* mice, with significantly more keratinocytes immunopositive for Ki67 and PCNA compared with control samples (Appendix Fig S2A–C). This was accompanied by dramatic upregulation of keratin 6, a marker for hyperproliferative, activated keratinocytes, found in wound healing (Fig 1H). In addition, we observed infiltration of inflammatory cells into the epidermis (exocytosis) with formation of micro‐abscesses and crusts. Immunolabeling for p53 and for cleaved‐caspase 3 revealed enhanced apoptosis in cells of the interfollicular epidermis and in hair follicles of P7 *Dnmt1^Δ^* ^/^ *^Δep^* mice (Appendix Fig S2D and E). Furthermore, immunoblot analysis of epidermal isolates revealed induced DNA damage, indicated by the presence of gamma‐H2A.X. (Appendix Fig S2F). Together, this analysis indicates that epidermal DNA hypomethylation induces a severe skin pathology.

### Elevated innate immune response in the *Dnmt1^Δ^* ^/^ *^Δep^* epidermis

**Figure 2:**
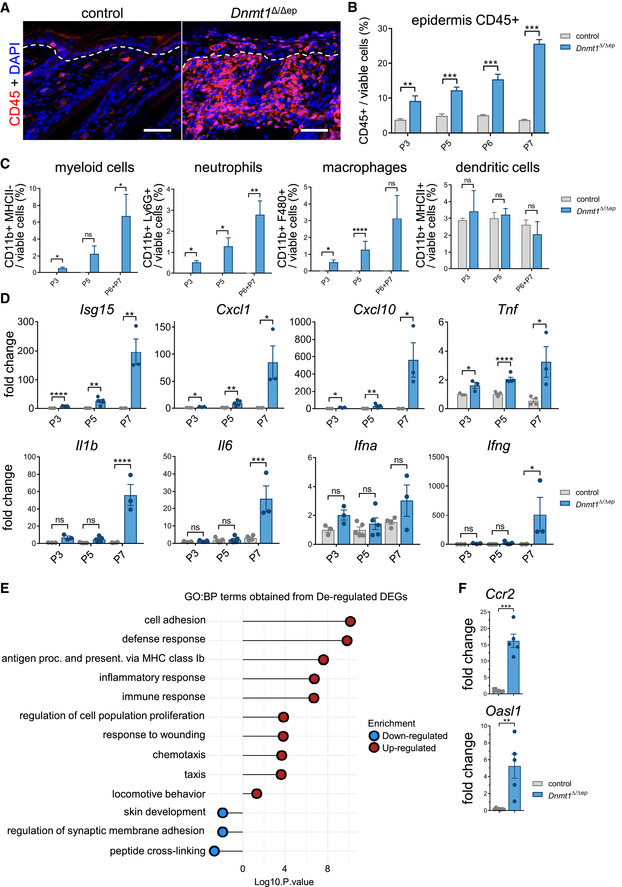
Epidermal DNA hypomethylation results in induced immune response Representative immunostaining for CD45 of dorsal skin sections from control and *Dnmt1^Δ^* ^/^ *^Δep^* mice at P7. Dashed lines indicate the dermal–epidermal border.Flow cytometric analysis of CD45‐positive cells isolated from the epidermis of control and *Dnmt1* ^Δ/Δep^ mice of different postnatal ages. Cells are shown as percent of viable cells. Data are mean ± SD, two‐tailed *t*‐test, **P ≤ 0.01, ***P ≤ 0.001, *n* ≥ 4 mice.Flow cytometric analysis of the respective immune cell populations from the epidermis of control and *Dnmt1* ^Δ/Δep^ mice of different postnatal ages. Cells are shown as percent of viable cells. Data are mean ± SEM, two‐tailed *t*‐test. ns, not significant, *P ≤ 0.05, **P ≤ 0.01, ****P ≤ 0.0001, *n* = 3 (P3), 4 (P5), and 4 (P6 + P7).Relative mRNA expression levels of immune‐related genes in control and *Dnmt1^Δ^* ^/^ *^Δep^* mice at P3 (*n* = 3), P5 (*n* = 5), and P7 (*n* ≥ 3). Data are mean ± SEM. *P*‐values are calculated using one‐way ANOVA with post hoc Tukey multiple comparison test, *P ≤ 0.05, **P ≤ 0.01, ***P ≤ 0.001, ****P ≤ 0.0001.Gene ontology analysis of significantly up‐ and downregulated genes (*p*‐value < 0.05) obtained from epidermal keratinocytes of control and *Dnmt1^Δ^* ^/^ *^Δep^* mice (*n* = 3 mice). Keratinocytes were isolated from 3‐day‐old mice and cultured for 72 h.Relative mRNA expression of representative immune‐related genes found to be deregulated by RNA sequencing in keratinocytes cultured for 72 h and isolated from 3‐day‐old control and *Dnmt1^Δ^* ^/^ *^Δep^* mice (*n* = 3 per group). Data are mean ± SEM, two‐tailed *t*‐test, **P ≤ 0.01; ***P ≤ 0.001. Data information: Scale bars, 50 µm.

**Figure EV2:**
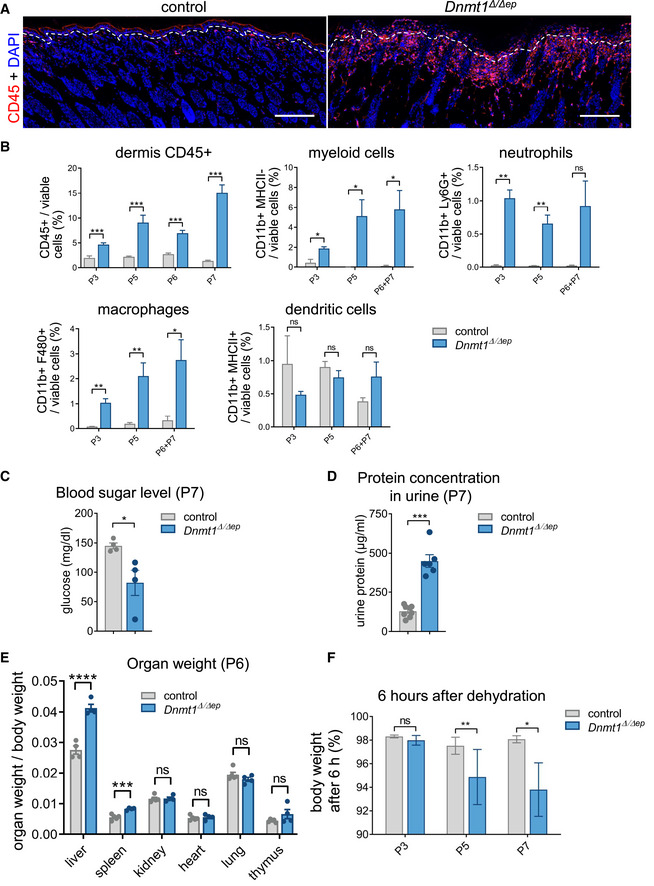
Epidermal DNA hypomethylation results in induced immune response and in systemic effects AImmunolabeling of dorsal skin sections from control and *Dnmt1^Δ^* ^/^ *^Δep^* mice for CD45. Dashed lines indicate dermal‐epidermal border or the edge of hair follicles.BFlow cytometric analysis of CD45‐positive cells and the respective immune cell populations in the dermis of control and *Dnmt1* ^Δ/Δep^ mice of different postnatal ages. Cells are shown as percent of viable cells. Data are mean ± SD, two‐tailed *t*‐test. ns, not significant, *P ≤ 0.05, **P ≤ 0.01, CD45, P3‐P7, *n* ≥ 4 mice. All other markers: *n* = 3 (P3), 4 (P5), and 4 (P6 + P7).C–EComparison of *Dnmt1^Δ^* ^/^ *^Δep^* and control mice regarding blood sugar level (C, *n* = 4), protein concentration of urine (D, *n* = 6), and organ weight (E, *n* ≥ 4).FThe inside out barrier of the skin of 3‐ to 7‐day‐old *Dnmt1^Δ^* ^/^ *^Δep^* and control pubs was tested by a dehydration assay, *n* = 5 (P3), *n* = 11 (P5, control), *n* = 6 (P5, *Dnmt1^Δ^* ^/^ *^Δep^*), *n* = 3 (P7, control), and *n* = 4 (P7, *Dnmt1^Δ^* ^/^ *^Δep^*). Extent of fluids loss was calculated by measuring the decrease of the weight during a certain time period and calculated as percent of body weight. Data information: For analysis of (C–F), two‐tailed Student's *t*‐test was used. Data are mean ± SEM. ns, not significant, *P ≤ 0.05, **P ≤ 0.01, ***P ≤ 0.001, ****P ≤ 0.0001. Scale bars, 200 µm.

**Figure EV3:**
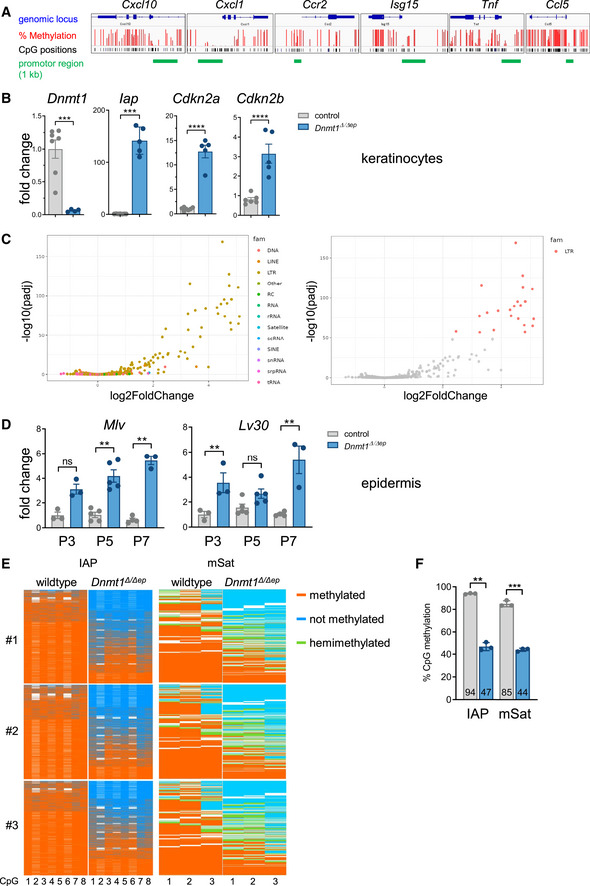
Keratinocytes of *Dnmt1^Δ^* ^/^ *^Δep^* mice exhibit DNA hypomethylation and derepression of transposon/repeat families ADNA methylation profile of wild‐type keratinocytes at selected upregulated genes upon knockout of *Dnmt1*. DNA Methylation data were obtained from Chatterjee *et al*, 2014 and visualized in the IGV browser. The proximal promotor regions show lower methylation levels than surrounding CpGs.BExpression of deregulated genes found by RNA sequencing of cultured primary keratinocytes that were isolated at P3 of control and *Dnmt1^Δ^* ^/^ *^Δe^* ^p^ mice (*n* = 5‐7). Statistical analyses were done using two‐tailed Student's *t*‐test. Data are mean ± SEM. ***P ≤ 0.001, ****P ≤ 0.0001.CChanges in expression of transposon/repeat families were plotted in a scatter plot of log fold change versus log10(adjusted *P*‐values).DRelative mRNA expression of transposable elements in the epidermis of control and *Dnmt1^Δ^* ^/^ *^Δep^* mice at P3 (*n* = 3), P5 (*n* = 5), and P7 (*n* = 3). Statistical analyses were done using one‐way ANOVA (Kruskal–Wallis test of multiple comparisons). Data are mean ± SEM. ns, not significant, **P ≤ 0.01.E, FDNA methylation of CD45‐depleted epidermal keratinocytes isolated from newborn wild‐type (*n* = 3) and *Dnmt1^Δ^* ^/^ *^Δe^* ^p^ (*n* = 3) mice. Methylation status of repetitive elements was analyzed using deep amplicon bisulfite sequencing (IAP‐LTR1a) or deep hairpin‐bisulfite sequencing (major Satellites, mSat1). Mean values of DNA methylation of wild‐type and *Dnmt1^Δ^* ^/^ *^Δe^* ^p^ mice are shown in (F), and statistical analysis was done using two‐tailed Student's *t*‐test (see also Supp. Tab. S4). Data are mean ± SEM. **P ≤ 0.01, ***P ≤ 0.001.

**Figure EV4:**
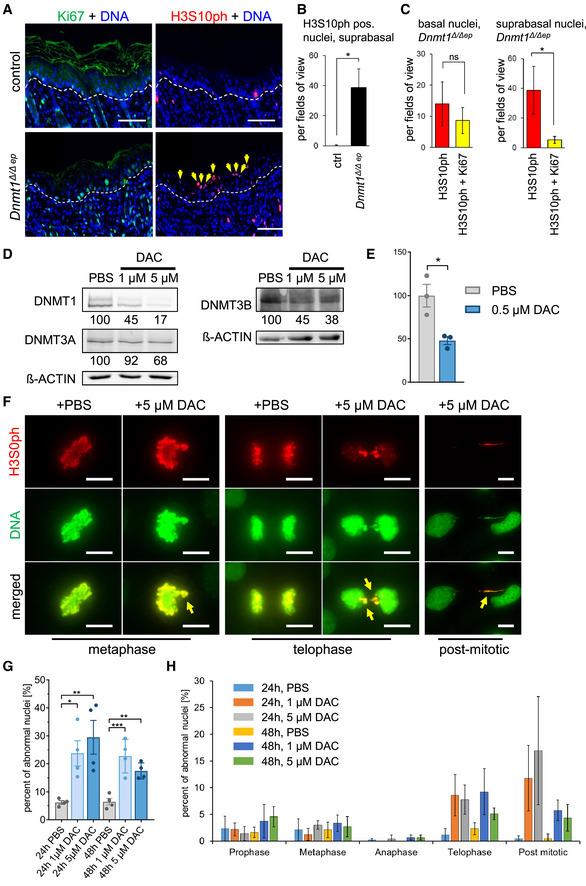
DNA hypomethylation results in defective mitosis and increase in H3S10ph‐positive G2/M cells in suprabasal epidermal layers A, BImmunofluorescence double‐labeling of G2/M cells (H3S10ph) and proliferating cells (Ki67) of dorsal skin sections from day 7 old control and *Dnmt1^Δ^* ^/^ *^Δep^* mice. Dashed lines indicate the dermal–epidermal border and arrows point to suprabasally located H3S10ph‐positive nuclei. (B) Quantification of suprabasal H3S10ph‐positive nuclei shown in (A) from day 7 old control (*n* = 4) and *Dnmt1^Δ^* ^/^ *^Δep^* mice (*n* = 4). Values were compared using two‐tailed Student's *t*‐test. Data are mean ± SEM. *P ≤ 0.05.CQuantification of basal (left) and suprabasal (right) cells from day 7 old control (*n* = 4) and *Dnmt1^Δ^* ^/^ *^Δep^* mice (*n* = 4) positive for H3S10ph and cells double positive for H3S10ph and Ki67 (as shown in A). Statistical analyses were done using two‐tailed Student's *t*‐test. Data are mean ± SEM. ns not significant, *P ≤ 0.05.D–FEffect of DAC treatment on expression of DNMTs and DNA methylation. (D) Cells were treated with 5‐aza‐2′‐deoxycytidine (DAC) or vehicle (PBS) for 72 h, and protein extracts were analyzed for expression of DNMT1, DNMT3A, and DNMT3B. ß‐Actin served as loading control. Signals were quantified relative to the ß‐Actin signal and indicated as percentage of the respective PBS control value. (E) 5‐meC quantification was performed by quantitative dot blot analysis. The PBS control value was set to 100%. The experiment was done in triplicates, and values were compared using two‐tailed Student`s *t*‐test. Data are mean ± SEM. *P ≤ 0.05. (f) Representative images from aberrant mitotic and post‐mitotic nuclei (H3S10ph‐positive) observed in human keratinocytes treated with DAC. Arrows indicate chromosomal fragments and nucleoplasmic bridges.GHuman keratinocytes were treated with PBS (control), 1 µM DAC, or 5 µM DAC for 24 or 48 h and immunostained for H3S10ph and DNA (PicoGreen). Slides were randomized and analyzed in a blinded manner for mitotic and post‐mitotic nuclear aberrations as shown in panel (F). About 100 H3S10ph‐positive mitotic and post‐mitotic nuclei per condition and replicate were analyzed for nuclear defects and calculated as percentage of the total number of H3S10ph‐positive mitotic and post‐mitotic nuclei. Statistical analyses were done using one‐way ANOVA with post hoc Tukey multiple comparison test. Data are mean ± SEM. *P ≤ 0.05, **P ≤ 0.01, ***P ≤ 0.001.HGraphical description of the abnormal nuclei during each cell cycle phase. Data are mean ± SEM from 4 biological replicates per condition. Data information: Scale bars, 10 µm (F), 100 µm (A).

**Figure EV5:**
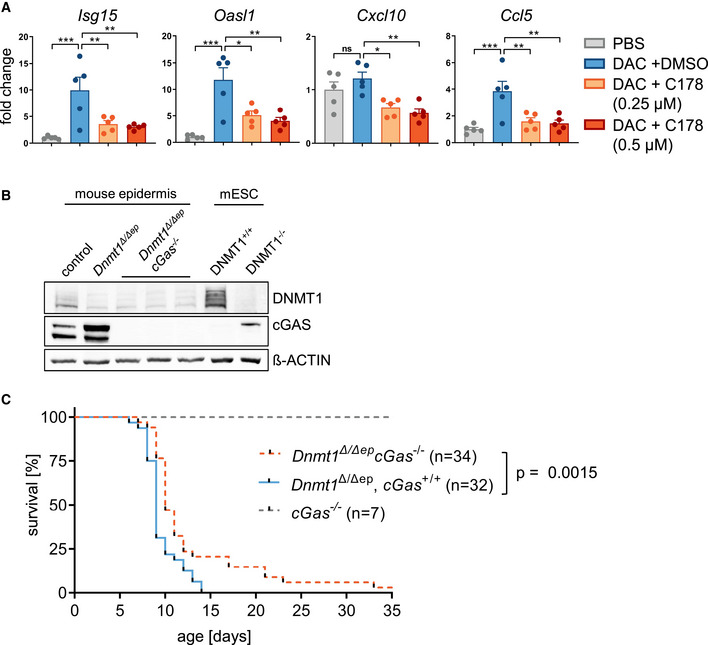
Ablation of the cGAS/STING pathway results in decreased expression of immune genes and increased lifespan of *Dnmt1^Δ^* ^/^ *^Δep^* mice Cultivated neonatal wild‐type primary mouse keratinocytes were treated with PBS (control) or 2'‐deoxy‐5‐azacytidine (DAC) for in total 72 h. After 48 h additionally, a specific STING inhibitor (C‐178) or DMSO was added, and 24 h later, cells were harvested. Expression of immune‐related genes was compared using one‐way ANOVA with post hoc Tukey multiple comparison test. Data are mean ± SEM from 4 biological replicates per condition. ns, not significant; *P ≤ 0.05, **P ≤ 0.01, ***P ≤ 0.001.Immunoblot analysis for expression of DNMT1 and cGAS in lysates of isolated mouse epidermis of control, *Dnmt1^Δ^* ^/^ *^Δep^*, and *Dnmt1^Δ^* ^/^ *^Δep^ Cgas* ^−/−^ mice. Additionally, mouse embryonic stem cells (mESC) with (DNMT1^−/−^) or without (DNMT1^+/+^) genetic deletion of *Dnmt1* were analyzed. Beta‐actin was used as loading control.Kaplan–Meier plot of control (*Cgas* ^−/−^, *n* = 7), *Dnmt1^Δ^* ^/^ *^Δep^ Cgas* ^−/−^ (*n* = 32), and *Dnmt1^Δ^* ^/^ *^Δep^ Cgas* ^−/−^ (*n* = 34) mice using log‐rank test (Mantel–Cox). *P* < 0.0015.

Pathological assessment of skin histology suggests that disrupted epidermal homeostasis in *Dnmt1^Δ^* ^/^ *^Δep^* mice is potentially the consequence of inflammatory disease. Thus, paraffin skin sections were immunolabeled for the presence of the common leukocyte antigen CD45. At P7, a massive increase in CD45‐positive cells was observed in both the epidermis and the dermis of *Dnmt1^Δ^* ^/^ *^Δep^* mice (Fig 2A). Although more leukocytes were found in all skin layers compared with control animals, immune cell infiltration was most obvious within the papillary dermis, with the highest density of immune cells found toward the dermal/epidermal border (Fig EV2A). To determine the dynamics of immune cell infiltration, flow cytometric analysis was performed from skin specimens at different postnatal days. The proportion of CD45‐positive cells in the epidermis and dermis increased over time in *Dnmt1* ^Δ/Δep^ mice compared with control samples (Figs 2B and EV2B). The increase in immune cells is mainly due to the CD11b+ myeloid population. Further analysis of markers showed that these cells represent neutrophils (CD11b^+^ Ly6G^+^) and macrophages (CD11b^+^ F4/80^+^), whereas the percentage of antigen‐presenting cells (LCs and DCs) remain largely unchanged (CD11b^+^ MHC‐II^+^) (Figs 2C and EV2B). Consistent with an increase in CD11b^+^ cells, qRT–PCR analysis of selected chemokines revealed that *Ifng* and interferon‐stimulated genes involved in innate immune responses, such as *Isg15*, *Cxcl1*, *Cxcl10*, *IL1b*, and *IL6*, were strongly induced in the epidermis, while *Tnfa* was moderately but significantly upregulated and *Ifna* was unchanged (Fig 2D). Furthermore, systemic effects such as reduced blood sugar levels, proteinuria, increased organ mass (liver and kidney), and reduced barrier function accompanied the immune phenotype and the early lethality of *Dnmt1^Δ^* ^/^ *^Δep^* mice (Fig EV2, EV3, EV4, EV5). These data suggest that *Dnmt1^Δ^* ^/^ *^Δep^* mice mount a strong innate immune response in the absence of infection.

To investigate whether the effects of DNMT1 ablation in keratinocytes are cell‐autonomous in the context of the innate immune response, RNA sequencing was performed using keratinocytes isolated from P3 *Dnmt1^Δ^* ^/^ *^Δep^* and control mice. We found 1,339 genes up‐ and 114 genes downregulated in the absence of DNMT1 (abs(log2FC) < 1; *P* ≤ 0.05) (Dataset EV1). Using gene ontology analysis of the deregulated genes, we found enrichment of several pathways that are highly connected to inflammation, including immune response, defense response, or chemotaxis (Fig 2E). Upregulation of several immune genes such as *Ccr2* and *Oasl1* was confirmed by qRT–PCR (Fig 2F). Notably, the promoter regions of most of these genes, except for *Ccr2*, are hypomethylated in control keratinocytes, suggesting that their induction in *Dnmt1^Δ^* ^/^ *^Δep^* keratinocytes is not a direct effect of local demethylation of regulatory regions in the absence of DNMT1 (Chatterjee *et al*, 2014) (Fig EV3A). With respect to proliferation, cultivated primary keratinocytes from *Dnmt1* ^Δ/Δep^ mice behaved differently when compared to keratinocytes *in vivo*. *In vitro*, *Dnmt1^Δ^* ^/^ *^Δep^* keratinocytes exhibited induction of the Cdk inhibitors *Cdkn2a* (*p16*) and *Cdkn2b* (*p15*) (Fig EV3B), while keratinocytes within the epidermis of *Dnmt1^Δ^* ^/^ *^Δep^* mice showed hyperproliferation (Appendix Fig S2A–C). This difference emphasizes the importance of examining the role of DNA methylation in the *in vivo* context of a given tissue. In summary, these findings show that DNA hypomethylation in the epidermis induces a strong immune response in the skin.

### The inflammatory phenotype of *Dnmt1^Δ^* ^/^ *^Δep^* mice does not depend on MDA5/MAVS signaling

**Figure 3:**
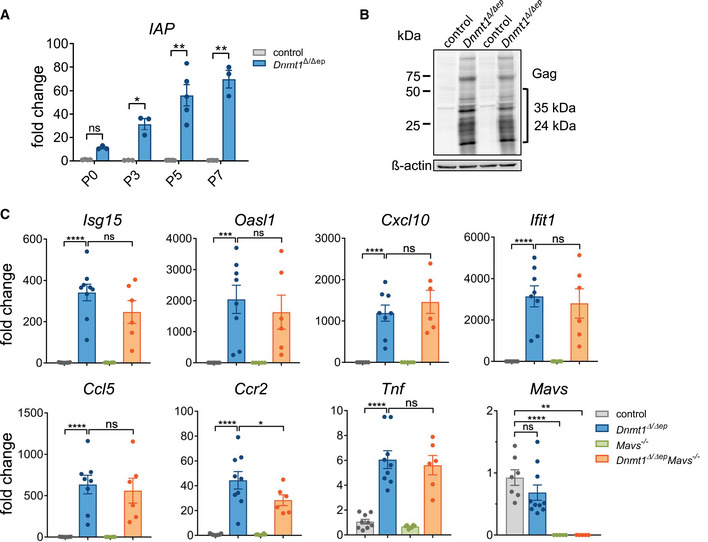
Epidermal deletion of DNMT1 results in derepression of transposable elements and activation of immune genes with minor impact of the MAVS pathway Relative mRNA expression levels of epidermal Intracisternal A‐type particles (IAPs) during different postnatal time points (P0 *n* ≥ 3, P3 *n* = 3, P5 *n* = 5, P7 *n* ≥ 3 mice), which were compared using one‐way ANOVA with post hoc Tukey multiple comparison test. Data are mean ± SEM. ns not significant, *P ≤ 0.05, **P ≤ 0.01.IAP protein was determined from epidermal isolates of two control and two *Dnmt1^Δ^* ^/^ *^Δep^* mice. A bracket indicates the size of group‐specific antigen (gag) proteins.Relative expression of immune‐related genes of the epidermis from P7 of control (*n* ≥ 7), *Dnmt1^Δ^* ^/^ *^Δep^*, (*n* ≥ 8), MAVS knockout (*Mavs* ^−/−^, *n* = 7*)*, and *Dnmt1^Δ^* ^/^ *^Δep^ Mavs* ^−/−^ (*n* = 6) mice was compared using one‐way ANOVA with post hoc Holm–Sidak multiple comparison test. Data are mean ± SEM. ns not significant, *P ≤ 0.05, **P ≤ 0.01, ***P ≤ 0.001, ****P ≤ 0.0001.

Previous studies have demonstrated that DNA‐demethylating agents activate the innate immune system via the MDA5 (melanoma differentiation‐associated protein 5) /MAVS (mitochondrial antiviral‐signaling) RNA recognition pathway in tumor cells (Chiappinelli *et al*, 2015; Roulois *et al*, 2015). This RNA‐sensing mechanism is induced by dsRNAs, which are in part derived from derepressed endogenous retroviral elements and its activation by viral mimicry was linked to the toxic effect of DNMT inhibitors on tumor cells (Licht, 2015). Analysis of transposon transcripts extracted from RNA sequencing data from *Dnmt1*‐deficient keratinocytes revealed that different classes and families of transposons, in particular LTR retrotransposons, are upregulated (Dataset EV2 and Fig EV3C). As expected (Gaudet *et al*, 2004), strong, time‐dependent activation of IAP (intracisternal A‐type particle) LTR retrotransposons as well as of MLV retroviruses and LV30 transposons was observed by qRT–PCR analysis of epidermal samples from P0‐P7 *Dnmt1^Δ^* ^/^ *^Δep^* mice (Figs 3A and EV3C and D) and immunoblot analysis of the IAP Gag protein (Fig 3B). A similar increase in IAP transcript levels was also detected in cultivated keratinocytes from *Dnmt1^Δ^* ^/^ *^Δep^* mice when compared to control samples (Fig EV3B). The increase in IAP transcripts was linked to reduced DNA methylation at IAP elements (Fig EV3E and F and Appendix Table S2). These data demonstrate that loss of DNMT1 in the epidermis results in DNA hypomethylation and derepression of transposons. Therefore, we examined whether activation of the MDA5/MAVS RNA recognition pathway is critical for the immune response and contributes to the skin phenotype observed in *Dnmt1^Δ^* ^/^ *^Δep^* mice. We generated double knockout mice lacking DNMT1 and functional MAVS, the essential mediator of dsRNA recognition by MDA5. However, the histopathological phenotype including immune cell infiltration, as well as survival of the mice, was found to be similar to DNMT1 single knockout (Appendix Figs S3A–E and S4). To analyze whether additional ablation of MAVS has an influence on transcript levels of immune‐related genes, qRT–PCR was performed from epidermal isolates of P7 mice. Most immune genes analyzed showed a tendency toward reduced levels upon additional deletion of *Mavs* in the *Dnmt1^Δ^* ^/^ *^Δep^* epidermis. However, with the exception of the chemokine *Ccr2*, none of these changes were statistically significant (Fig 3C). Taken together, these data indicate that induction of the innate immune system in the epidermis of *Dnmt1^Δ^* ^/^ *^Δep^* mice is independent of transposon transcript recognition by the MDA5/MAVS pathway.

### DNA hypomethylation induces the cGAS/STING pathway via cytoplasmic DNA

**Figure 4:**
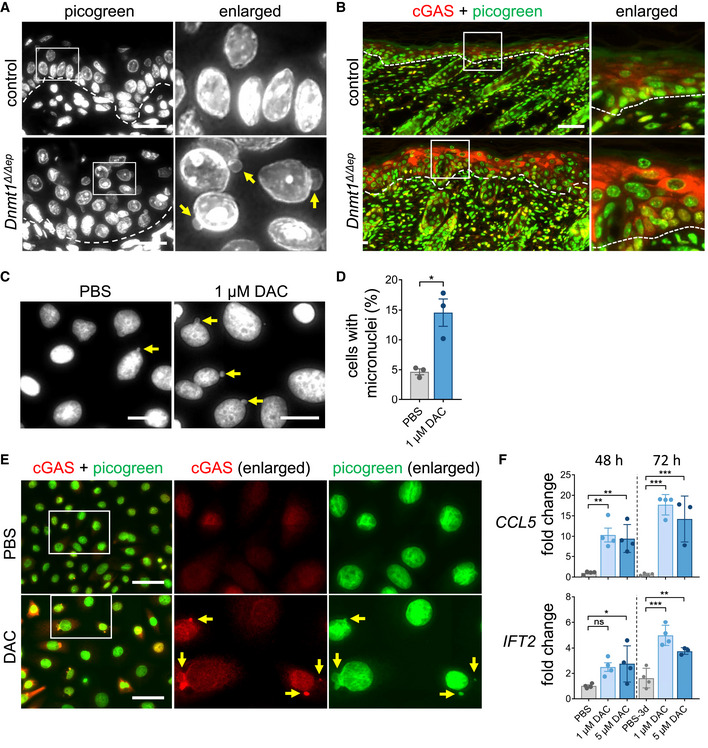
DNA hypomethylation results in release of cytoplasmic DNA and cGAS activation AParaffin skin sections (P5) of control and *Dnmt1^Δ^* ^/^ *^Δep^* mice were stained for DNA using PicoGreen. Enlarged sections are indicated on the right where arrows point to DNA‐positive nuclear protrusions. Dashed lines indicate the dermal–epidermal border.BLabeling of dorsal skin sections of control and *Dnmt1^Δ^* ^/^ *^Δep^* mice for cGAS and PicoGreen. Enlarged sections are indicated on the right. Dashed lines indicate the dermal–epidermal border.C–FImmortalized human keratinocytes (NHEK SV‐Tert3‐5) were treated with 5‐aza‐2′‐deoxycytidine (DAC) or vehicle (PBS). (C, D) PicoGreen staining of PBS‐ and DAC‐treated keratinocytes for 48 h detected micronuclei (arrow), which were analyzed in a blinded manner, and compared using two‐tailed *t*‐test. Data are mean ± SEM, *P ≤ 0.05 (*n* = 3 biological replicates) (D). (E) Co‐labeling of DNA by PicoGreen and by cGAS in PBS‐ and DAC‐treated keratinocytes. Within enlarged sections on the right arrows point at DNA/cGAS double‐positive blebs and micronuclei. (F) Relative expression of CCL5 and IFT2 in keratinocytes treated with PBS or DAC for 45 h or 72 h using one‐way ANOVA with post hoc Dunnett multiple comparison test. Data are mean ± SEM. ns not significant, *P ≤ 0.05, **P ≤ 0.01, ***P ≤ 0.001 (*n* = 4 biological replicates). Data information: Scale bars, 20 µm (A, D), 50 µm (B, E).

The *Dnmt1^Δ^* ^/^ *^Δep^* epidermis displays increased DNA damage and apoptosis. Previous studies demonstrated that DNA hypomethylation in human colon cancer cells induced genomic instability and the formation of micronuclei (Stopper *et al*, 1993; Costa *et al*, 2016). To investigate whether epidermal cells of *Dnmt1^Δ^* ^/^ *^Δep^* mice show similar nuclear aberrations, paraffin sections of the *Dnmt1^Δ^* ^/^ *^Δep^* epidermis were stained with the ultra‐sensitive DNA dye PicoGreen and microscopically evaluated. Epidermal *Dnmt1* deletion induced the formation of DNA blebs and micronuclei (Fig 4A). Importantly, these structures were not detected in the epidermis of control mice. Cytoplasmic double‐stranded DNA originating from viruses or chromosomal instability in senescent or malignant cells is sensed by the cGAS/STING pathway, which in turn induces interferon‐stimulated gene (ISG) expression (Dou *et al*, 2017; Gluck *et al*, 2017; Mackenzie *et al*, 2017; Bakhoum *et al*, 2018). In addition, the promoter region of the *Cgas* gene contains two adjacent IFN‐sensitive response elements and different pattern recognition receptor ligands induce cGAS expression in an IFN‐I‐dependent manner, indicating that cGAS itself can be regarded as an ISG (Ma *et al*, 2015). Therefore, we analyzed the expression of cGAS protein in the epidermis and found substantially increased levels of cGAS in the *Dnmt1* ^Δ/Δep^ epidermis compared with wild‐type control both by immunolabeling and immunoblot analysis (Figs 4B and EV5B).

Next, we studied the relationship between DNMT1 inactivation and formation of micronuclei in *in vitro* cultured immortalized human keratinocytes. The drug 5‐aza‐2′‐deoxycytidine (DAC) leads to DNMT inhibition and thus DNA hypomethylation (Stresemann & Lyko, 2008). Accordingly, DAC treatment of human keratinocytes for 72 h led to a reduction in protein levels of DNMT1 and the *de novo* methyltransferases DNMT3A and DNMT3B and reduced DNA methylation (Fig EV4D and E). Compared to control cells, DAC‐treated keratinocytes generated significantly more micronuclei as shown by DNA labeling using PicoGreen (Fig 4C and D). Moreover, the majority of detected micronuclei recruited cGAS, as shown by double staining of DNA and anti‐cGAS antibody (Fig 4E). Consistent with induction of an innate immune response, increased expression levels of the immune‐related *CCL5* and *IFT2* were observed in DAC‐treated keratinocytes (Fig 4F). DNA hypomethylation at pericentric chromosomal regions has been linked to defective mitosis, chromosome segregation errors, and chromosomal instability resulting in the formation of micronuclei, nuclear blebs, and nucleoplasmic bridges (Fenech *et al*, 2011; Costa *et al*, 2016). Indeed, visualization of mitotic chromosomes with an antibody specific for histone H3S10ph showed increased numbers of incomplete and disorganized mitosis in DAC‐treated keratinocytes (Fig EV4, EV5). In particular, aberrant mitotic figures during telophase and nucleoplasmic bridges in post‐mitotic cells were significantly elevated upon DAC treatment. Similarly, loss of *Dnmt1* led to DNA hypomethylation at pericentric repeats in murine keratinocytes (Fig EV3D and E). Analysis of H3S10ph‐positive G2/M cells in the *Dnmt1^Δ^* ^/^ *^Δep^* epidermis revealed a significant increase in G2/M cells in the suprabasal layers, which are normally devoid of G2/M cells as seen in control mice (Fig EV4, EV5). Interestingly, the H3S10ph‐positive G2/M cells in the suprabasal layers are negative for the proliferation marker Ki67, indicating that they are arrested in mitosis. Taken together, these findings suggest that loss of DNMT activity in keratinocytes induces chromosomal instability and disrupts cell cycle progression resulting in the formation of DNA blebs and micronuclei. The detection of cytosolic DNA by cGAS induces a strong innate immune response in *Dnmt1* ^Δ/Δep^ mice.

### Deletion of cGAS ameliorates the autoimmune phenotype of *Dnmt1* ^Δ/Δep^ mice

To examine whether cGAS/STING activation contributes to the autoinflammatory phenotype in response to DNA hypomethylation, we treated primary keratinocytes with DAC and a novel STING inhibitor C‐178 (Haag *et al*, 2018). DAC treatment of primary keratinocytes alone led to induction of immune genes including *Isg15*, *Oasl1*, and *Ccl5* (Fig EV5A). Importantly, simultaneous treatment with the STING inhibitor C‐178 resulted in significantly reduced activation of innate response genes. To further test the hypothesis that cGAS represents a critical mediator of pro‐inflammatory signals in the hypomethylated epidermis, we generated *Dnmt1* ^Δ/Δep^ *Cgas* ^−/−^ double knockout mice (Fig EV5B). The additional knockout of *Cgas* resulted in a significantly higher survival rate of mice when compared to *Dnmt1* ^Δ/Δep^ mice (Fig EV5C). The severely impaired epidermal homeostasis observed in the *Dnmt1* ^Δ/Δep^ mice before the full onset of an immune response suggests that DNMT1‐dependent effects that are not linked to the innate immune response contribute to the lethal phenotype. This is also supported by the study of Sen *et al*. (Sen *et al*, 2010) using a xenograft model with SCID mice. These immunocompromised mice show in the absence of DNMT1 severe epidermal defects independent of an activated immune system.

**Figure 5:**
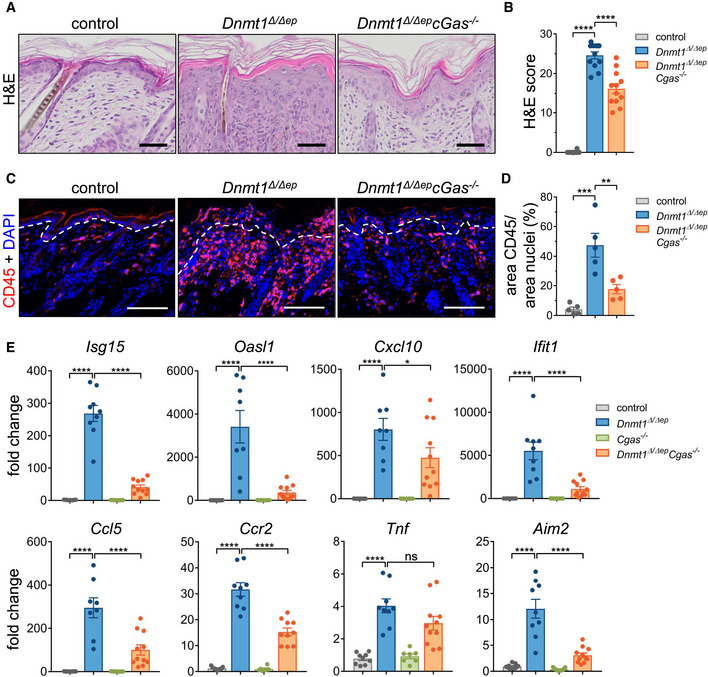
Additional deletion of cGAS attenuates the autoinflammatory *Dnmt1^Δ^* ^/^ *^Δep^* skin phenotype Representative images of P7 H&E‐labeled dorsal skin sections of control (*Dnmt1^f^* ^/^ *^f^)*, *Dnmt1^Δ^* ^/^ *^Δep^*, and *Dnmt1^Δ^* ^/^ *^Δep^ Cgas* ^−/−^ mice.The histopathology of at least 9 animals per genotype was assessed in a blinded manner, and an overall score was determined from individual parameters (see Appendix Fig 7 and Appendix Table S3). Comparison was performed using one‐way ANOVA with post hoc Holm–Sidak multiple comparison test. Data are mean ± SEM. ****P ≤ 0.0001.Representative images of CD45 immunofluorescence labeling of P7 dorsal skin sections from *Dnmt1^f^* ^/^ *^f^*, *Dnmt1^Δ^* ^/^ *^Δep^*, and *Dnmt1^Δ^* ^/^ *^Δep^ Cgas* ^−/−^ mice. Dashed lines indicate the dermal‐epidermal border.Quantification of CD45‐positive cells. Tissue sections approximately 1 cm in length were scanned, and the area of CD45‐positive cells per area of nuclei was quantified using ImageJ. Sections obtained from five mice per genotype were analyzed, and calculated values were compared using one‐way ANOVA with post hoc Holm–Sidak multiple comparison test. Data are mean ± SEM. **P ≤ 0.01, ***P ≤ 0.001.Epidermis obtained from P7 control, *Dnmt1^Δ^* ^/^ *^Δep^*, *Cgas* ^−/−^, and *Dnmt1^Δ^* ^/^ *^Δep^ Cgas* ^−/−^ mice was analyzed for relative mRNA expression levels of immune‐related genes using one‐way ANOVA with post hoc Holm–Sidak multiple comparison test. Data are mean ± SEM. ns not significant, *P ≤ 0.05, ****P ≤ 0.0001. At least 7 mice per genotype were used. Data information: Scale bars, 50 µm (A), 100 µm (C).

Importantly, histoscoring of H&E‐labeled paraffin skin sections revealed significant improvement of several histopathological parameters (Fig 5A and B, and Appendix Fig S4). Likewise, immune cell infiltration into the dermis and epidermis was strongly reduced upon additional deletion of *Cgas*, as shown by immunolabeling for CD45 (Fig 5C and D). To test, whether expression of immune‐related genes reflected improvement of skin pathology in *Dnmt1* ^Δ/Δep^ *Cgas* ^−/−^, epidermal isolates from P7 mice were analyzed. qRT–PCR revealed significant downregulation of interferon‐stimulated genes (*Isg15*, *Ifit1*, *Oasl1*) and of chemokines and cytokines (*Ccr2*, *Ccl5*, *Cxcl10*) (Fig 5E). In agreement with previously published data (de Koning *et al*, 2012), loss of epidermal DNMT1 resulted in a robust induction of AIM2 indicating a role of the inflammasome in the activation of the innate immune response. Interestingly, the AIM2 induction was largely dependent on the presence of cGAS supporting the idea of a cross‐talk between the cGAS and the inflammasome as previously described (Swanson *et al*, 2017). Taken together, these results indicate that activation of the innate immune system via the cGAS/STING pathway is a critical component of the inflammatory skin disease seen in *Dnmt1* ^Δ/Δep^ mice.

## Discussion

Our study reveals a crucial role for DNA methylation in epidermal homeostasis through both maintenance of genomic stability and prevention of inappropriate innate immune system activation. Mice with a *Keratin 5‐Cre*‐mediated deletion of *Dnmt1* show a normal epidermal development until birth. From P3 onwards, mice develop a severe skin phenotype characterized by disturbed epidermal homeostasis, and immune cell infiltration reflecting an excessive immune response. Sen *et al*. (Sen *et al*, 2010) demonstrated that loss of DNMT1 in human keratinocytes resulted in impaired self‐renewal and premature differentiation of epidermal progenitors. Indeed, *in vitro* cultivated murine *Dnmt1*‐deficient keratinocytes express increased levels of the senescence marker p15 and p16, reflecting in part the behavior of human *DNMT1* knockdown keratinocytes (Sen *et al*, 2010). In contrast, we observed hyperproliferation of keratinocytes in the epidermis of *Dnmt1^Δ^* ^/^ *^Δep^* mice, which might be caused by the presence of immune cells and resident dermal cells, leading to mutual stimulation *in vivo (*Zheng *et al*, 2007; Briso *et al*, 2013 *)*. Another study using *Keratin 14‐Cre* to delete *Dnmt1* in the epidermis reported reduced epidermal stem cell proliferation and hair germ formation in the absence of DNMT1 (Li *et al*, 2012). These mice are viable, and their epidermis shows uneven thickness due to hyperproliferative regions. The changes in epidermal proliferation were interpreted as arising from a compensatory mechanism (Li *et al*, 2012). Divergent results observed in these studies can be explained by differences in the origin of keratinocytes, the mouse models used, and the absence of immune cells in the xenograft model. The usage of an immunocompetent, conditional K5‐cre mouse model allowed us to conduct a robust analysis of *Dnmt1* deletion in the skin.

Several reports have implicated DNMT1 in the regulation of mammalian cell fate decisions in different tissues and organs. *Dnmt1* deletion during embryogenesis caused an acute phenotype of the intestine, characterized by weight loss, global DNA hypomethylation, genome instability, apoptosis, and loss of nascent villi (Elliott *et al*, 2015) (mitotic genes were top hits among downregulated genes). In the mature intestinal epithelium, DNMT1 controls cellular differentiation, but is dispensable for organ maintenance and organismal survival in adult mice (Sheaffer *et al*, 2014). In the nervous system, DNMT1 has been shown to be essential for survival of fetal mitotic neuroblasts. Mice with *Nestin‐Cre*‐mediated deletion of *Dnmt1* die after birth due to respiratory defects (Fan *et al*, 2001). In the pancreas, ablation of DNMT1 results in a decrease in differentiated pancreatic cells with a concomitant increase in p53 levels, cell cycle arrest, and progenitor cell apoptosis (Georgia *et al*, 2013). Taken together, all these studies indicate a regulatory role of DNMT1 in the control of progenitor self‐renewal and differentiation. The present study links tissue‐specific loss of DNMT1 to innate immune system activation and systemic defects, providing a holistic view of the impact of deregulated DNA methylation at the organismal level.

We here report for the first time that autoinflammation of the skin is a direct consequence of DNMT1 ablation *in vivo*. DNA hypomethylation in the epidermis results in the activation of the innate immune system, likely linked to the skin’s unique function as an immune organ consisting of highly proliferating basal keratinocytes and skin‐resident and infiltrating immune cells (Kabashima *et al*, 2019; Nguyen & Soulika, 2019). We find that *Dnmt1^Δ^* ^/^ *^Δep^* mice show an elevated innate immune response in the epidermis, immune cell infiltration in the skin, and systemic effects in response to inflammation. *In vitro* experiments with keratinocytes demonstrated that the elevated expression of immune genes in response to DNA hypomethylation is at least in part a cell‐intrinsic effect. Our data demonstrate that DNMT1 loss in the epidermis induces aberrant changes in cellular nucleic acid abundance and localization, triggering a strong innate immune response.

In accordance with previous reports on other cell types (Fan *et al*, 2001; Gaudet *et al*, 2004; Georgia *et al*, 2013), the *Dnmt1^Δ^* ^/^ *^Δep^* epidermis displays a dramatic increase in the expression of IAPs and other transposons. An RNA‐dependent immune response to DNA hypomethylation was previously reported for human tumor cells upon treatment with DNMT inhibitor (Chiappinelli *et al*, 2015; Roulois *et al*, 2015). In this setting, the MDA5/MAVS pathway is activated in response to retrotransposon‐derived double‐stranded RNA. In a follow‐up study, it was shown that upon inhibition of DNA methylation, intronic and intergenic SINEs activate MDA5 in cancer cells in an ADAR1‐dependent manner (Mehdipour *et al*, 2020). Accordingly, loss of the RNA‐editing enzyme ADAR1 causes an enhanced interferon response and embryonic lethality, which is rescued by additional *Mavs* knockout (Mannion *et al*, 2014). In the context of epidermal DNA hypomethylation, we did not detect a significant contribution of MDA5/MAVS signaling to the observed phenotypes. It remains possible that transposon activation contributes to the immune phenotype of *Dnmt1^Δ^* ^/^ *^Δep^* mice. LINE1 retrotransposon activation in response to depletion of the histone deacetylase SIRT6 leads to accumulation of cytoplasmic L1 cDNA, which triggers a strong type I interferon response via cGAS activation (Simon *et al*, 2019). Future work will test whether other nucleic acid‐sensing mechanisms are activated by (reverse) transcription of mobile elements.

A hallmark of *Dnmt1*‐deficient or 5‐aza‐2′‐deoxycytidine‐treated keratinocytes in our study is incomplete or defective mitosis resulting in the presence of cytosolic DNA, detected as blebs, micronuclei and nucleoplasmic bridges. DNA hypomethylation has been previously shown to induce genomic instability, DNA damage, and mitotic arrest in human cancer cells (Chen *et al*, 2007; Costa *et al*, 2016), and in combination with Poly(ADP ribose) polymerase inhibition, it causes inflammation and reduced DNA repair (McLaughlin *et al*, 2020). In macrophages, DNMT3A induces HDAC9 to deacetylate TBK1 for activation of antiviral innate immunity (Li *et al*, 2016). Mutations in the human DNMT3B gene result in ICF (immunodeficiency, centromeric region instability, and facial anomalies syndrome) (Xu *et al*, 1999). ICF patient cells show genomic instability highlighted by the presence of micronuclei and nuclear blebs. Our findings further highlight a crucial function for DNA methylation in the maintenance of chromosomal stability. Cytosolic DNA in the *Dnmt1^Δ^* ^/^ *^Δep^ epidermis* and 5‐aza‐C‐treated keratinocytes was recognized by cGAS, resulting in upregulation of the DNA‐sensor protein by feedback. Ablation of cGAS in *Dnmt1^Δ^* ^/^ *^Δep^* mice significantly attenuated the activation of immune genes, infiltration of immune cells, and destruction of the skin architecture suggesting a major contribution of the cGAS/STING pathway to the autoinflammatory phenotype of *Dnmt1^Δ^* ^/^ *^Δep^* mice. Mice deficient in the DNA exonuclease *Trex1* develop systemic inflammation (Morita *et al*, 2004) ameliorated by simultaneous deficiency in STING or cGAS (Gray *et al*, 2015). Similarly, deletion of *Cgas* or *Sting* in mice lacking DNase II, a lysosomal enzyme that digests DNA, rescued the lethal autoimmune phenotypes of the DNase II knockout mice (Ahn *et al*, 2012; Gao *et al*, 2015). Taken together, DNA‐modifying and processing enzymes prevent the accumulation of cytosolic DNA and thereby the unwanted activation of the innate immune system by self DNA.

Additional loss of cGAS does not fully abolish the autoinflammatory phenotype of *Dnmt1^Δ^* ^/^ *^Δep^* mice. It is conceivable that other pathways of the innate immune system such as TLR3 (dsRNA), TLR2/TLR4 (protein), the inflammasome (DNA, RNA, protein), TLR9 (hypomethylated ssDNA, RNA/DNA hybrids), or ZBP1 (Z‐DNA, Z‐RNA) are also induced in the epidermis of these mice (Kawai & Akira, 2011; Rigby *et al*, 2014; Bartok & Hartmann, 2020; Devos *et al*, 2020; Jiao *et al*, 2020). Gene mutations resulting in defective DNA/RNA modification and turnover pathways are associated with autoinflammatory diseases (Uggenti *et al*, 2019; Bartok & Hartmann, 2020; Melki & Fremond, 2020). In addition to genetic factors, also epigenetic dysregulation contributes to the development of pathogenesis (Alvarez‐Errico *et al*, 2017). Therefore, it will be interesting to investigate whether changes in epigenetic patterns and nucleic acid‐sensing mechanisms in the skin also contribute to autoinflammatory disorders.

The data presented here have potential implications for cancer treatment. cGAS/STING agonists have been considered as promising therapeutics in combination with immune checkpoint inhibitors in particular (Flood *et al*, 2019; Hoong *et al*, 2020). Current efforts to advance immunotherapy focus on testing drug combinations involving epigenetic drugs, which stimulate both innate and adaptive immune responses (Jones *et al*, 2019; Hogg *et al*, 2020) (Daver *et al*, 2018; Lai *et al*, 2021). Our study indicates that in addition to activation of RNA‐sensing pathways *via* derepression of mobile elements, DNMT inhibitors could be used to trigger the controlled activation of the immune system through DNA‐sensing pathways. Both processes might contribute to an increased efficacy of a combination of epigenetic and immune checkpoint therapies in tumor treatment.

## Materials and Methods

### Animal care, transgenic mouse lines, and mouse experiments

All mouse lines were bred to a mixed genetic background of C57BL/ 6J 129SV. *Keratin 5*‐Cre (*K5‐Cre*) mice (Ramirez *et al*, 2004) were mated to *Dnmt1^f^* ^/^ *^f^* (Jackson‐Grusby *et al*, 2001; Lee *et al*, 2001), *Mavs* ^−/−^ (Sun *et al*, 2006), and *Cgas* ^−/−^ (Schoggins *et al*, 2014) mice to generate mice with deletions of *Dnmt1* in the epidermis or epidermal *Dnmt1*, *Mavs*, and *Cgas*, respectively. Mice were kept in the animal facilities of the Medical University of Vienna in accordance with institutional policies and federal guidelines. All animal experiments were performed in accordance with a protocol authorized by the Austrian Ministry of Science and Research, following the approval by the National Ethical Committee for Animal Experimentation. The Austrian Ministry for Science and Research approved the corresponding proposals GZ BMWFW‐66.009/0138‐WF/V/3b/2017 and GZ BMWFW‐66.009/0276‐WF/V/3b/2017.

#### Genotyping primer

*K5‐Cre* forward and reverse (TAA TCG CCA TCT TCC AGC AG and CAA TTT ACT GAC CGT ACA C), *Dnmt1 flox* forward and reverse (AAC CGT TGG CTT TTT GAG TGA G and AGA AAT AAA AAG CCA GTT GTG T), *Cgas* forward, reverse 1, reverse 2 (ATA TTT CCC CCT GTG TTG GA, GTG CCA GGT GAC ACA ACA TC, CGG ATG GAT GAA CAA ACA GA), *Mavs 1*, *2*, *3* (AGC CAA GAT TCT AGA AGC TGA GAA, TAG CTG TGA GGC AGG ACA GGT AAG G and GTG GAA TGT GTG CGA GGC CAG AGG C).

For the dye exclusion assay, embryos (E19.5) were killed and incubated for 1 min in 25, 50, and 75% methanol in PBS, followed by a 1‐min incubation in 100% methanol, and a descending series of incubations in 75, 50, and 25% methanol in PBS for 1 min. Pups were washed in PBS and stained with 0.1% toluidine blue O (Sigma) for 1 min. Destaining was done in PBS. Blue‐stained navel served as positive control for successful staining.

Dehydration assay was performed to measure the extent of fluid loss during a certain time frame and reflects the dysfunction of the skin barrier. Pups were separated from their parents on the indicated postnatal day (P3‐P9) and kept at 37°C in the incubator for 6 h. Their weight was measured every 30 min, and weight loss was calculated as a function of time in relation to the starting weight of the pubs (Leyvraz *et al*, 2005).

Blood sugar was monitored with a glucose meter. Spontaneous urine was collected and analyzed with the pyrogallol red‐molybdate method, according to Fujita *et al*. performed by the company In Vitro Laboratory for veterinary Diagnostics and Hygiene GmbH Vienna, Austria.

### Epidermis isolation and keratinocyte culture

Murine skin was isolated and incubated with the dermis side in PBS containing 5 mg/ml Dispase (Roche) for 2 h, at 4°C for protein and RNA isolation and at 37°C for keratinocyte isolation. Epidermis was removed from dermis, washed in PBS, and snap‐frozen for protein and RNA analysis. In case of RNA isolation, solutions were supplemented with RNAse Inhibitor (Winter *et al*, 2013). Mouse epidermal keratinocytes were isolated as previously described (Sibilia *et al*, 2000) and cultured in keratinocyte growth medium (KGM Gold BulletKit, Lonza) containing 8% chelated fetal calf serum and 0.05 mM CaCl_2_ on collagen‐fibronectin‐coated dishes. Immortalized human epidermal keratinocytes NHEK/SVTERT3‐5 (Wagner *et al*, 2018) (EVERCYTE, Vienna, Austria, CLHT‐011‐0026‐5) were grown in KGM2 (C‐20011, PromoCell) adjusted to 0.06 mM CaCl_2_ and propagated according to the manufacturer’s instruction.

### Treatment and immunofluorescence (IF) analysis of keratinocytes

Cells were seeded either onto glass coverslips (for IF) placed in 6‐well plates or into 12‐well plates (for RNA expression analyses) one day before treatment. On the next day, cells were treated with either the vehicle control PBS or the DNMT1 inhibitor 5‐Aza‐2′‐deoxycytidine (DAC; Sigma‐Aldrich). To inhibit the cGas/Sting pathway, the cells were treated with the inhibitor C‐178 (generous gift from Andrea Ablasser (Haag *et al*, 2018)) 24 h after the first DAC treatment. Keratinocytes were harvested either 24 h (IF) or 48 h (IF, RNA) after DAC treatment. For IF analysis, cells were washed in ice‐cold PBS, fixed in 4% paraformaldehyde for 20 min at 4°C, washed with PBS, and stored at 4°C until further analysis. For IF, cells were permeabilized using 0.1% Triton X in PBS for 20 min at room temperature than blocked in 2% BSA in PBS with 10% goat serum for 30 min at room temperature. Antibodies specific for human cGAS (#D1D3G, Cell signaling Technology, 1:200) and ph‐Histone Histone H3, Ser 10 (SC‐8658‐R, Santa Cruz Biotechnology, 1:2,000) were overnight incubated at 4°C. After washing three times with PBS containing 0.1% Tween‐20, the coverslip sections were incubated with Alexa Fluor 546‐conjugated secondary antibody (Fisher Scientific) for 1 h at room temperature. After washing three times with PBS containing 0.1% Tween‐20, the samples were counterstained with 1 µg/ml 4′,6‐diamidino‐2‐phenylindol (DAPI, Sigma‐Aldrich) and PicoGreen dsDNA Reagent (Fisher Scientific, 1:2,000) for 20 min in TE buffer (10 mM Tris–HCl, 1 mM EDTA, pH 8.0). Coverslips were subsequently washed 3 times in PBS and mounted on SuperFrost Plus Adhesion slides (Thermo Scientific) using ProLong Gold (Thermo Fisher Scientific).

### Determination of chromosomal aberrations during mitosis

Human keratinocytes treated with DAC for 24 or 48 h were immunolabeled for histone H3S10ph and counterstained with PicoGreen. About 100 mitotic H3S10ph‐positive nuclei were analyzed in a blinded and randomized manner, classified into mitotic phases, and assessed for chromosomal aberrations.

### Hematoxylin and eosin (H&E) labeling, immunohistochemistry (IHC), immunofluorescence (IF), and TUNEL analyses

Tissue samples were fixed overnight in 4% PFA and further embedded in paraffin. Stainings were performed on 4‐µm sections using SuperFrost Plus Adhesion slides (Thermo Scientific). H&E stainings were carried out according to the standard procedure with a COT 20 Linear Slide Stainer (Medite). IF and IHC analyses were carried out according to protocols published previously (Fischer *et al*, 2005) with modifications. Briefly, paraffin sections were prepared for staining by heat antigen retrieval using a pressure cooker (2100 Antigen Retriever, Aptum Biologics) for 20 min at 121°C. Depending on the used antibody, the slides were cooked in 10 mM Citrate buffer (pH 6) or in Dako Target Retrieval Solution (pH 9; Agilent). Samples were cooled down and incubated with phosphate‐buffered saline, pH 7.2, plus 2% BSA and plus 10% goat serum (DAKO) for 20 min to block nonspecific binding. The slides were incubated overnight at 4°C using a humid chamber. For IHC, samples were incubated with the respective secondary antibody, stainings were visualized with the VECTASTAIN ABC kit and DAB substrate (Vector labs) and sections were counterstained with hematoxylin. For IF, sections were incubated with the appropriate Alexa Fluor 488/546‐conjugated secondary and counterstained with 1 µg/ml 4′,6‐diamidin‐2‐phenylindol (DAPI, Sigma‐Aldrich) and PicoGreen dsDNA Reagent (Fisher Scientific, 1:2,000) for 20 min in TE buffer (10 mM Tris–HCl, 1 mM EDTA, pH 8.0). The slides were mounted with Prolong Gold (Thermo Fisher Scientific). For *in situ* labeling of 3′‐OH ends of DNA, the TUNEL assay (Roche) was performed according to the manufacturer’s instructions (Fischer *et al*, 2005).

#### Primary antibodies used for IHC

Ki67 (1:500, Novocastra), Keratin 10, Keratin 6, Loricrin (1:1,000, Covance), p53 (1:50, NCL‐p53‐CM5p, Novocastra), cleaved‐caspase 3 (1:200, CC3, cs‐96615, Cell Signaling), PCNA (1:200, DAKO), DNMT1 (1:200, Abcam, ab188453), CGAS (1:100 Cell Signaling, 31659; 1:200 Cell Signaling 15102) 5 mC (1:500, Diagenode 006‐500), CD45 (1:1,000, Abcam ab10558), and histone H3S10ph (1:2,000, Santa Cruz Biotechnology SC‐8656‐R).

### Microscopy, image processing, and image analysis

H&E, IHC, and immunolabeling were analyzed using a slide scanner (VS120 S6, Olympus) equipped with a XM10 monochrome camera (Olympus) and a Pike F‐505C color camera (Allied Vision). Image scans were obtained and further processed using the following Programs: VS‐ASW 2.9, OlyVia (Olympus) and Adobe Photoshop CS6 (Adobe). For some IF analyzes, images were taken using a LSM Meta 510 confocal microscope (Zeiss). It was ensured that identical exposure times and gray value settings were used for the samples to be compared. For automated image quantification, a script was developed using Fiji software (Schindelin *et al*, 2012). In brief, a tissue mask was interactively created corresponding to the tissue region of interest. Particle analysis was done after applying a fix threshold to allow a comparative analysis. For enhanced particle separation, a watershed algorithm was used. Particle analysis revealed the parameters particle count, area, and area fraction of the tissue region of interest. For the quantification of Ki67, positive cells in the interfollicular epidermis (IFE), positive cells of the basal, and suprabasal layers of the IFE were counted and set in relation to the total cell number.

### Histopathology

Paraffin sections of back skin which were stained by H&E and by azan were scanned using a slide scanner (VS120 S6, Olympus) equipped with 40x objective and a Pike F‐505C color camera (Allied Vision). Scanned images were analyzed by a certified pathologist in a blinded manner using the OlyVia software (Olympus). Samples were scored for nine pathological alterations and graded from 0 to 5. For details of analyzed alterations and grading system, see Appendix Fig S4 and Appendix Table S1. All scores were summed up, and total H&E score is given in the figure.

### Quantification of 5‐methylcytosine (5‐meC)

For 5‐meC quantification in the mouse epidermis, genomic DNA was extracted using the phenol/chloroform method. Briefly, mouse epidermis (as isolated described above) was incubated overnight in 4.5 ml proteinase K buffer with 300 μl proteinase K (20 mg/ml, 50 mM Tris–HCl pH8.0, 100 mM NaCl) and 250 μl SDS at 55°C. Then, 200 μl of 5 M NaCl and 5 ml phenol/chloroform were added. This was vortexed for 30 sec and then centrifuged for 5 min at 1,150 *g* at 4°C. The aqueous phase was subsequently transferred to a new tube, and 5 ml of chloroform was added. This was vortexed and centrifuged again, and the aqueous phase was put into a fresh tube. Three volumes of 96% ethanol were added, and DNA was then fished out with a glass hook, was centrifuged again, and washed in 70% ethanol. After drying the pellet, it was resuspended and dissolved in 400 μl TE buffer (1 M Tris–HCl pH 8.0, 0.5 M EDTA) for 5 min at 65°C. HPLC analysis of isolated DNA was performed as previously described (Ramsahoye, 2002). Genomic methylation was assessed by measuring methyl group carrying and unmodified cytidine nucleotides. HPLC shows the percentage of deoxy‐methyl‐cytidine‐mono‐phosphate (dmCMP) from total cytidines (dmCMP and dCMP). For 5‐meC quantification in human keratinocytes, DNA methylation was analyzed by quantitative dot blot analysis as previously described (Zupkovitz *et al*, 2021).

### DNA methylation analysis of selected repetitive elements

Epidermis of P7 mice was prepared by treatment with Dispase II solution (5 mg/ml in 1x PBS; Roche) for 45 min, 37°C. Epidermal cells were isolated by shaking in DNase I solution containing 250 µg/ml DNase I (DN25, Sigma‐Aldrich), 8% chelated fetal bovine serum (F2524, Sigma‐Aldrich) in MEM (Sigma‐Aldrich) for 30 min at 37°C. After filtration using a 70‐µm cell strainer, CD45‐positive cells were depleted using Mojosort Mouse CD45 Nanobeads (480027, BioLegend) according to manufacturer’s instructions. Purity of epidermal keratinocytes was checked by flow cytometry using anti‐CD45. Genomic DNA of keratinocytes was prepared using Genelute Mammalian Genomic DNA Kit (G1N70 Sigma‐Aldrich). DNA methylation status of selected repetitive elements was analyzed using deep amplicon bisulfite sequencing (IAP‐LTR1a) or deep hairpin‐bisulfite sequencing (major Satellites) according to Arand *et al*, 2012 (Arand *et al*, 2012), with slight modifications. Briefly, after bisulfite conversion, DNA was bound to MagBinding Beads (Zymo Research) in 7 M guanidine‐HCl buffer. After washing with 80% EtOH in 10 mM Tris–HCl pH 8.0, DNA was desulfonated using 0.1 M NaOH in 70% isopropanol followed by two washes with 80% EtOH in 10 mM Tris–HCl pH 8.0 and elution in water. Amplicons were amplified using fusion primers of the repeat‐specific primer and part of the TruSeq adaptor sequence. A second amplification step with six cycles was performed using full TruSeq adaptor sequences involving custom dual‐index barcodes. Analysis for IAP‐LTR1a (specific primer sequences: GAT AGT TGT GTT TTA AGT GGT AAA TAA A and TCC CTA TTA AAA AAA ATT ATC CTT C) omitted restriction and ligation reaction before bisulfite treatment. After equimolar pooling of purified amplicons, the library was sequenced on a MiSeq nano 2 × 150 bp run. Reads were demultiplexed and trimmed for adaptor sequences using cutadapt v2.10 (Martin, 2011). Paired reads were merged using flash (Magoc & Salzberg, 2011), and different amplicons were separated using cutadapt v2.10 by primer sequence. CpG methylation was determined using BiQAnalyzerHT (Lutsik *et al*, 2011) according to Arand *et al* (2012). More than 1,300 reads were obtained for every sample, with an average conversion rate of 99.5% as determined from the Hairpin adaptor sequence (see Appendix Table S2).

### DNA methylation profile at selected genes upregulated in *Dnmt1^∆^* ^/^ *^∆ep^* mice

DNA methylation status of the genomic loci of immune genes derived from wild‐type keratinocytes was obtained from Chatterjee *et al*, 2014 (Chatterjee *et al*, 2014) and visualized in IGV browser (http://software.broadinstitute.org/software/igv/home), Broad Institute and UC San Diego, CA.

### RNA isolation and real‐time PCR analysis

Total RNA was isolated following the manufacturer’s instructions (TRIzol, Invitrogen). RNA was reversely transcribed with the iScript cDNA Synthesis Kit (Bio‐Rad). Real‐time PCR analysis was performed with the KAPA SYBR FAST qPCR MasterMix (Peqlab) on an iCycler IQ system (Bio‐Rad). Relative expression levels were normalized to HPRT (mouse) or *B2 M* (human).

#### qPCR primer for mRNA expression of mouse genes

*Hprt* forward (5′) and reverse (3`) (GCTGGTGAAAAGGACCTCT and CACAGGACTAGAACACCTGC *Cxcl1* forward and reverse (CTG CAC CCA AAC CGA AGT C and AGC TTC AGG GTC AAG GCA AG); *Cxcl10* forward and reverse (TTC TGC CTT CAT CCT GCT G and AGA CAT CTC TGC TCA TCA TTC); *Cldn1* forward and reverse (CTT CTC TGG GAT GGA TCG and GGG TTG CCT GCA AAG TAC TGT); *Cldn3* forward and reverse (GCG GCT CTG CTC ACC TTA GT and GAC GTA GTC CTT GCG GTC GTA); *Cldn8* forward and reverse (TCA GAA TGC AGT GCA AGG TC and AGC CGG TGA TGA AGA AGA TG), *Cdkn2a* forward and reverse (GAA CTC TTT CGG TCG TAC CCC and CAG TTC GAA TCT GCA CCG TAG); *Cdkn2b* forward and reverse (CCC TGC CAC CCT TAC CAG A and CCC TGC CAC CCT TAC CAG A); *Isg15* forward and reverse (AAG CAG ACT CCT TAA TTC C and CTG TAC CAC TAG CAT CAC); *Tnf* forward and reverse (CAG GAG GGA GAA CAG AAA C and ACA AGC AGG AAT GAG AAG AG); *Oasl1* forward and reverse (ACC AGC AGT ATG TGA GAG and GGT CCA GTA GAT ACA GAG G); *Ccr2* forward and reverse (ACG ATG ATG GTG AGC CTT GTC and CAG GAA GAG CAG GTC AGA GAT G); *IAP* forwards and reverse (ACT AAC TCC TGC TGA CTG G and TGT GGC TTG CTC ATA GAT TAG); *Dnmt1ex3‐4* forward and reverse (GAG GAA GGC TAC CTG GCT AAA G and TCA CTG TCC GAC TTG CTT CTC); *MLV* forward and reverse (CCA GAC TTG ATC CTG CTA CA and CTC AGT CAG CCA TCT CTG AC); *LV30* forward and reverse (CTC GCA CCT TTT CGC GCT CG and GCG AGG GGA TCA TCA TAA CA); Ccl5 forward and reverse (ATA TGG CTC GGA CAC CAC TC and CCC ACT TCT TCT CTG GGT TG); *Mavs* forward and reverse (GGA CAC ACT CTG GGG ACT CT and GGT CAG GGA TGT TGT GAC CT); Ifit1 forward and reverse (GCT CTG CTG AAA ACC CAG AG and CCC AAT GGG TTC TTG ATG TC); *Ccl5* forward and reverse (CCC TCA CCA TCA TCC TCA CT and GAG CAC TTG CTG CTG GTG TA); *Aim2* forward and reverse (CCT GAT TCA AAG TGC AGG TG and GAG GCA GCA GAG CAG TTT TC); *Il1b* forward and reverse (ACT CAT TGT GGC TGT GGA GA and TTG TTC ATC TCG GAG CCT GT); Il6 forward and reverse (GAG GAT ACC ACT CCC AAC AGA CC and AAG TGC ATC ATC GTT GTT CAT ACA); *Ifna* forward and reverse (CGC AGG AGA AGG TGG ATG and TGC TGG TGG AGG TCA TTG); *Ifng* forward and reverse (GCT GAC CTA GAG AAG ACA CAT C and TTC CAC ATC TAT GCC ACT TGA G).

#### qPCR primer for mRNA expression of human genes

*B2M* forward and reverse (GAG TAT GCC TGC CGT GTG AAC and TCT AAG TTG CCA GCC CTC CTA G); *CCL5* forward and reverse (CTG CTT TGC CTA CAT TGC CC and TCG GGT GAC AAA GAC GAC TG); *IFT2* forward and reverse (CAA AGG GCA AAA CGA GGC AG and CCC AGG CAT AGT TTC CCC AG).

### RNA sequencing and data analysis

For RNA sequencing, epidermis of three control and three *Dnmt1^∆^* ^/^ *^∆ep^* mice was isolated at P3 and keratinocytes were cultured as described above for 5 days. RNA was extracted as described above and then used for RNA sequencing. For transcriptome sequencing (RNA‐seq) experiments, RNA was subjected to poly(A) selection with a Dynabeads mRNA purification kit (Invitrogen), followed by reverse transcription using a NEB RNA Ultra kit and library generation using a TruSeq library generation kit (Illumina). We performed full‐length mRNA‐seq experiments in three biological replicates for *Dnmt1^∆^* ^/^ *^∆ep^* and the corresponding control keratinocytes and applied the “union” model of the htseq‐ count script (Anders *et al*, 2015) to calculate the number of reads associated with each of the 21,608 mouse RefSeq genes for each sample. We used these counts to compute reads per kilobase per million (RPKM) values for each gene and determined Spearman’s correlation coefficient (*p*) for each set of biological replicates. Based on the high correlation of the replicates (*P* = 0.99 between each 2 of the 3 control samples and between each 2 of the 3 knockout samples), we used the log‐transformed means of RPKM values under each condition to plot the distribution of gene expression levels by using kernel density estimation. Based on this distribution, we set the threshold for gene expression to 1 RPKM (log‐RPKM value equal to zero). This is consistent with data from previous studies, which estimated that the value of 1 RPKM corresponds to 1 transcript per cell (Mortazavi *et al*, 2008). The analysis of differentially expressed genes across the two conditions was performed by using htseq‐count and the Bioconductor edgeR package (Gentleman *et al*, 2004; Robinson *et al*, 2010). Genes that showed a minimum of a 2‐fold change in expression levels (adjusted *P*‐value of ≤ 0.05) were classified as upregulated, whereas genes displaying a fold change of ≤ 0.5 (adjusted *P*‐value of ≤ 0.05) were categorized as downregulated. The cutoffs for differential expression were set as absolute fold change > 2, and a corrected *P*‐value of > 0.05. GO analysis was performed with the DAVID (https://david.ncifcrf.gov/). The EntrezGeneIDs of the genes of interest were used as input for the web‐based analysis tools. The genes were functionally classified according to the Gene Ontology Consortium annotation category GO biological process.

#### Repeat analysis

To analyze repeat enrichment, we mapped the reads with bowtie (Langmead *et al*, 2009) (v 1.2.0, ‐m1 ‐‐max multi.fastq) against the human reference genome (GRCh38) or the mouse reference genome (MGSCv37). The uniquely aligning reads and the multi‐mappers were assigned to repeat classes, families, and types with RepEnrich (Criscione *et al*, 2014). The significantly differentially detected repeats were called using DESeq2 (Love *et al*, 2014) (v 1.10.1). The repeat count table from repseq was annotated with the class/family name of the repeat and plotted in a scatter plot of log fold change versus log10(adjusted *P*‐values).

#### RNA‐seq analysis

The 3' adaptors were removed using cutadapt (v1.4.2, Read1: cutadapt ‐‐match‐read‐wildcards ‐O 1 ‐a AGA TCG GAA GAG CAC ACG TCT GAA CTC CAG TCA C), and trimmed reads with a length of less than 18 bp were discarded. The trimmed reads were filtered with a contaminants database consisting of rDNA sequences (KY962518.1, NR_023363.1 and the ERCC.fa) with bowtie2 (v2.1.0 ‐‐very‐sensitive‐local). The recovered nonmatching reads were aligned to the genome/transcriptome using STAR‐align (v2.4.2a, STAR ‐‐outSAMstrandField None ‐‐outFilterIntronMotifs RemoveNoncanonical ‐‐outFilterMismatchNoverLmax 0.1 ‐‐outFilterMismatchNmax 10 ‐‐outFilterScoreMinOverLread 0.30 ‐‐outFilterMatchNminOverLread 0.30 ‐‐outFilterMatchNmin 30 ‐‐chimSegmentMin 15 ‐‐quantMode TranscriptomeSAM ‐‐chimJunctionOverhangMin 15 ‐‐twopassMode Basic ‐‐outSAMtype SAM ‐‐outSAMattributes All ‐‐outReadsUnmapped Fastx intronMotif ‐‐alignIntronMax 200000 ‐‐outSJfilterIntronMaxVsReadN 10000 20000 30000 50000 ‐‐outSJfilterOverhangMin 20 12 12 12 ‐‐outFilterType BySJout ‐‐alignMatesGapMax 0 ‐‐outFilterMultimapNmax 20). The STAR‐index used for alignment was generated from the human genome build NCBI GRCh38 (GCA_000001405.15) and a transcriptome GTF file from ENSEMBL build 78. Unique mappers from the resulting BAM file were used to create unstranded, stranded and reverse stranded coverage tracks for visualization using bedtools (v2.27, bedtools genomecov ‐split ‐bg, bedtools genomecov ‐split ‐bg ‐strand “+”, bedtools genomecov ‐split ‐bg ‐strand “‐”) and bedGraphToBigWig from the kent‐tools (bedGraphToBigWig v4, bedGraphToBigWig ‐blockSize=256 ‐itemsPerSlot=1024). Normalized coverage tracks were created by dividing the coverage at each position with the total number of aligned reads per million. The differentially expressed genes were called using DESeq2 (Love *et al*, 2014) (v 1.10.1).

### Protein and histone isolation and immunoblot analysis

Protein and isolation from skin and cultured keratinocytes for immunoblot analysis and subsequent immunoblot signal quantification were performed as previously described (Winter *et al*, 2013).

#### Primary antibodies for immunoblotting

DNMT1 (1:500, sc‐20701, Santa Cruz Biotechnology), DNMT3a (1:1,000, sc‐20703, Santa Cruz Biotechnology), DNMT3b (1:1,000, sc‐376043, Santa Cruz Biotechnology), Actin (1:20,000; A5316, Sigma), Lamin B (1:1,000, sc‐6216, Santa Cruz Biotechnology), H3 C‐terminal (1:500,000, ab1791, Abcam), IAP (1:500, kindly provided by the Bryan R. Cullen lab (Bogerd *et al*, 2006), Center for Virology and Department of Molecular Genetics and Microbiology, Duke University Medical Center, Durham), PCNA (1:1,000, C1 PC10, Dako), γH2A.X S139ph (1:1,000, ab2893, Abcam), and cGas (1:1,000, 31659, Cell Signaling).

### FACS analysis

Dermis and epidermis from pups were separated after overnight incubation in dispase solution (see above) at 4°C. Epidermis was cut into small pieces and incubated for 30 min at 37°C in 0.2 mg/ml DNAse I (Sigma) in RPMI. Dermis was cut and treated with RPMI containing DNAse and 1.6 mg/ml collagenase IV (Worthington) for 30 min at 37°C. A single cell suspension was prepared by passing the dissociated tissue through a 70‐μm cell strainer. Cells were washed with FACS buffer (1% BSA, 0.1% NaN3, PBS), and unspecific antibody binding was blocked with anti‐mouse CD16/CD32 (eBioscience). Approximately 1 × 10E6 cells per tube were stained with respective antibodies for 30 min at 4°C. 7AAD (Sigma) was added shortly before measurement for dead cell discrimination. Data were acquired on an FACS‐Aria and analyzed with FlowJo software according to the gating strategy shown in Appendix Fig S10.

#### Primary antibodies used for FACS

CD45 APC‐Cy7 (1:100, BioLegend, clone 30‐F11), MHC‐II PE (1:1,000, BioLegend, clone M5), CD11b PE‐Cy7 (1:100, BioLegend, clone M1/70), F4/80 APC (1:50, eBioscience, clone BM8), Ly6G PE (1:50, BioLegend), and 7AAD PerCP (2.5 μl/sample, Sigma).

### Quantifications and Statistical analysis

#### Quantification of Ki67 and Histone H3S10ph (H3S10ph) epidermal cells

Paraffin sections of back skin from P7 wild‐type and *Dnmt1^∆^* ^/^ *^∆ep^* mice were double immunolabeled for Ki67 and H3S10ph. For quantification, three random images per mouse were chosen and the number of basal and suprabasal epidermal H3S10ph‐positive and Ki67/H3S10ph double‐positive nuclei were counted in a blinded manner. The obtained triplicate values were averaged and compared with an unpaired two‐tailed *t*‐test.

#### Statistical analysis

In general, measurements were taken from distinct samples. qRT–PCR and quantification experiments were evaluated with Microsoft Excel. Immunoblot signal intensities were quantified using the ImageQuants software, and relative protein levels were normalized to beta‐Actin or Lamin B. The significance between groups was determined by the unpaired Student’s *t*‐test or by ANOVA. *P*‐values were calculated with the GraphPad Prism software, and standard deviation (s.d.) or standard error of the mean (SEM) is shown. *P ≤ 0.05; **P ≤ 0.01; ***P ≤ 0.001; ns = not significant (*P* > 0.05).

#### Quantification of micronuclei

PicoGreen‐stained cells were scanned using a 100x oil immersion objective. The scans were randomized, and 6 images each were blindly selected for analysis. Micronucleus‐associated nuclei were counted along with the total number of nuclei.

## Author contributions

CSe, MAB, HF, and MW conceptualized the study. CSe, MAB, HF, MW, MS, and AA designed the study. MAB, HF, MW, LG, SS, PW, TG, TM, BZ, CFi, CFo, JA, BR, SL, GM, AP, MK, GE, MM, and CSch carried out experiments. ST, UR, MG, PP, and LK analyzed data. IT and RST carried out bioinformatic analysis. CSe, MAB and HF wrote the manuscript.

## Conflict of interest

The authors declare that they have no conflict of interest.

## Supporting information



Appendix

Expanded View Figures PDF

Dataset EV1

Dataset EV2

## Data Availability

The RNA‐seq datasets have been deposited to the NCBI Gene Expression Omnibus (GEO) under GSE166356 (http://www.ncbi.nlm.nih.gov/geo/query/acc.cgi?acc=GSE166356).

## References

[embj2021108234-bib-0001] Ablasser A , Hur S (2020) Regulation of cGAS‐ and RLR‐mediated immunity to nucleic acids. Nat Immunol 21: 17–29 31819255 10.1038/s41590-019-0556-1

[embj2021108234-bib-0002] Ahn J , Gutman D , Saijo S , Barber GN (2012) STING manifests self DNA‐dependent inflammatory disease. Proc Natl Acad Sci USA 109: 19386–19391 23132945 10.1073/pnas.1215006109 PMC3511090

[embj2021108234-bib-0003] Alonso L , Fuchs E (2003) Stem cells of the skin epithelium. Proc Natl Acad Sci USA 100(Suppl 1): 11830–11835 12913119 10.1073/pnas.1734203100 PMC304094

[embj2021108234-bib-0004] Alvarez‐Errico D , Vento‐Tormo R , Ballestar E (2017) Genetic and epigenetic determinants in autoinflammatory diseases. Front Immunol 8: 318 28382039 10.3389/fimmu.2017.00318 PMC5360705

[embj2021108234-bib-0005] Anders S , Pyl PT , Huber W (2015) HTSeq–a Python framework to work with high‐throughput sequencing data. Bioinformatics 31: 166–169 25260700 10.1093/bioinformatics/btu638 PMC4287950

[embj2021108234-bib-0006] Ambrosi C , Manzo M , Baubec T (2017) Dynamics and context‐dependent roles of DNA methylation. J Mol Biol 429: 1459–1475 28214512 10.1016/j.jmb.2017.02.008

[embj2021108234-bib-0007] Arand J , Spieler D , Karius T , Branco MR , Meilinger D , Meissner A , Jenuwein T , Xu G , Leonhardt H , Wolf V *et al* (2012) In vivo control of CpG and non‐CpG DNA methylation by DNA methyltransferases. PLoS Genet 8: e1002750 22761581 10.1371/journal.pgen.1002750 PMC3386304

[embj2021108234-bib-0008] Bakhoum SF , Ngo B , Laughney AM , Cavallo J‐A , Murphy CJ , Ly P , Shah P , Sriram RK , Watkins TBK , Taunk NK *et al* (2018) Chromosomal instability drives metastasis through a cytosolic DNA response. Nature 553: 467–472 29342134 10.1038/nature25432 PMC5785464

[embj2021108234-bib-0009] Bartok E , Hartmann G (2020) Immune Sensing Mechanisms that Discriminate Self from Altered Self and Foreign Nucleic Acids. Immunity 53: 54–77 32668228 10.1016/j.immuni.2020.06.014 PMC7359798

[embj2021108234-bib-0010] Bogerd HP , Wiegand HL , Doehle BP , Lueders KK , Cullen BR (2006) APOBEC3A and APOBEC3B are potent inhibitors of LTR‐retrotransposon function in human cells. Nucleic Acids Res 34: 89–95 16407327 10.1093/nar/gkj416 PMC1326241

[embj2021108234-bib-0011] Briso EM , Guinea‐Viniegra J , Bakiri L , Rogon Z , Petzelbauer P , Eils R , Wolf R , Rincon M , Angel P , Wagner EF (2013) Inflammation‐mediated skin tumorigenesis induced by epidermal c‐Fos. Genes Dev 27: 1959–1973 24029918 10.1101/gad.223339.113 PMC3792473

[embj2021108234-bib-0012] Chatterjee R , He X , Huang D , FitzGerald P , Smith A , Vinson C (2014) High‐resolution genome‐wide DNA methylation maps of mouse primary female dermal fibroblasts and keratinocytes. Epigenetics Chromatin 7: 35 25699092 10.1186/1756-8935-7-35 PMC4333159

[embj2021108234-bib-0013] Chen T , Hevi S , Gay F , Tsujimoto N , He T , Zhang B , Ueda Y , Li E (2007) Complete inactivation of DNMT1 leads to mitotic catastrophe in human cancer cells. Nat Genet 39: 391–396 17322882 10.1038/ng1982

[embj2021108234-bib-0014] Chiappinelli K , Strissel P , Desrichard A , Li H , Henke C , Akman B , Hein A , Rote N , Cope L , Snyder A *et al* (2015) Inhibiting DNA methylation causes an interferon response in cancer via dsRNA including endogenous retroviruses. Cell 162: 974–986 26317466 10.1016/j.cell.2015.07.011 PMC4556003

[embj2021108234-bib-0015] Costa G , Barra V , Lentini L , Cilluffo D , Di Leonardo A (2016) DNA demethylation caused by 5‐Aza‐2'‐deoxycytidine induces mitotic alterations and aneuploidy. Oncotarget 7: 3726–3739 26771138 10.18632/oncotarget.6897 PMC4826165

[embj2021108234-bib-0016] Criscione SW , Zhang Y , Thompson W , Sedivy JM , Neretti N (2014) Transcriptional landscape of repetitive elements in normal and cancer human cells. BMC Genom 15: 583 10.1186/1471-2164-15-583 PMC4122776 25012247

[embj2021108234-bib-0017] Daver N , Boddu P , Garcia‐Manero G , Yadav SS , Sharma P , Allison J , Kantarjian H (2018) Hypomethylating agents in combination with immune checkpoint inhibitors in acute myeloid leukemia and myelodysplastic syndromes. Leukemia 32: 1094–1105 29487386 10.1038/s41375-018-0070-8 PMC6916728

[embj2021108234-bib-0018] Devos M , Tanghe G , Gilbert B , Dierick E , Verheirstraeten M , Nemegeer J , de Reuver R , Lefebvre S , De Munck J , Rehwinkel J *et al* (2020) Sensing of endogenous nucleic acids by ZBP1 induces keratinocyte necroptosis and skin inflammation. J Exp Med 217: e20191913 32315377 10.1084/jem.20191913 PMC7336309

[embj2021108234-bib-0019] Dou Z , Ghosh K , Vizioli MG , Zhu J , Sen P , Wangensteen KJ , Simithy J , Lan Y , Lin Y , Zhou Z *et al* (2017) Cytoplasmic chromatin triggers inflammation in senescence and cancer. Nature 550: 402–406 28976970 10.1038/nature24050 PMC5850938

[embj2021108234-bib-0020] Elliott EN , Sheaffer KL , Schug J , Stappenbeck TS , Kaestner KH (2015) Dnmt1 is essential to maintain progenitors in the perinatal intestinal epithelium. Development 142: 2163–2172 26023099 10.1242/dev.117341 PMC4483766

[embj2021108234-bib-0021] Espada J , Esteller M (2010) DNA methylation and the functional organization of the nuclear compartment. Semin Cell Dev Biol 21: 238–246 19892028 10.1016/j.semcdb.2009.10.006

[embj2021108234-bib-0022] Fan G , Beard C , Chen RZ , Csankovszki G , Sun YI , Siniaia M , Biniszkiewicz D , Bates B , Lee PP , Kühn R *et al* (2001) DNA hypomethylation perturbs the function and survival of CNS neurons in postnatal animals. J Neurosci 21: 788–797 11157065 10.1523/JNEUROSCI.21-03-00788.2001 PMC6762314

[embj2021108234-bib-0023] Fenech M , Kirsch‐Volders M , Natarajan AT , Surralles J , Crott JW , Parry J , Norppa H , Eastmond DA , Tucker JD , Thomas P (2011) Molecular mechanisms of micronucleus, nucleoplasmic bridge and nuclear bud formation in mammalian and human cells. Mutagenesis 26: 125–132 21164193 10.1093/mutage/geq052

[embj2021108234-bib-0024] Fischer H , Rossiter H , Ghannadan M , Jaeger K , Barresi C , Declercq W , Tschachler E , Eckhart L (2005) Caspase‐14 but not caspase‐3 is processed during the development of fetal mouse epidermis. Differentiation 73: 406–413 16316411 10.1111/j.1432-0436.2005.00046.x

[embj2021108234-bib-0025] Flood BA , Higgs EF , Li S , Luke JJ , Gajewski TF (2019) STING pathway agonism as a cancer therapeutic. Immunol Rev 290: 24–38 31355488 10.1111/imr.12765 PMC6814203

[embj2021108234-bib-0026] Fuchs E , Horsley V (2008) More than one way to skin. Genes Dev 22: 976–985 18413712 10.1101/gad.1645908 PMC2732395

[embj2021108234-bib-0027] Gao D , Li T , Li XD , Chen X , Li QZ , Wight‐Carter M , Chen ZJ (2015) Activation of cyclic GMP‐AMP synthase by self‐DNA causes autoimmune diseases. Proc Natl Acad Sci USA 112: E5699–E5705 26371324 10.1073/pnas.1516465112 PMC4620884

[embj2021108234-bib-0028] Gaudet F , Rideout 3rd WM , Meissner A , Dausman J , Leonhardt H , Jaenisch R (2004) Dnmt1 expression in pre‐ and postimplantation embryogenesis and the maintenance of IAP silencing. Mol Cell Biol 24: 1640–1648 14749379 10.1128/MCB.24.4.1640-1648.2004 PMC344181

[embj2021108234-bib-0029] Gentleman RC , Carey VJ , Bates DM , Bolstad B , Dettling M , Dudoit S , Ellis B , Gautier L , Ge Y , Gentry J *et al* (2004) Bioconductor: open software development for computational biology and bioinformatics. Genome Biol 5: R80 15461798 10.1186/gb-2004-5-10-r80 PMC545600

[embj2021108234-bib-0030] Georgia S , Kanji M , Bhushan A (2013) DNMT1 represses p53 to maintain progenitor cell survival during pancreatic organogenesis. Genes Dev 27: 372–377 23431054 10.1101/gad.207001.112 PMC3589554

[embj2021108234-bib-0031] Gluck S , Guey B , Gulen MF , Wolter K , Kang TW , Schmacke NA , Bridgeman A , Rehwinkel J , Zender L , Ablasser A (2017) Innate immune sensing of cytosolic chromatin fragments through cGAS promotes senescence. Nat Cell Biol 19: 1061–1070 28759028 10.1038/ncb3586 PMC5826565

[embj2021108234-bib-0032] Gray EE , Treuting PM , Woodward JJ , Stetson DB (2015) Cutting edge: cGAS is required for lethal autoimmune disease in the Trex1‐deficient mouse model of Aicardi‐Goutieres Syndrome. J Immunol 195: 1939–1943 26223655 10.4049/jimmunol.1500969 PMC4546858

[embj2021108234-bib-0033] Gudjonsson JE , Krueger G (2012) A role for epigenetics in psoriasis: methylated Cytosine‐Guanine sites differentiate lesional from nonlesional skin and from normal skin. J Invest Dermatol 132: 506–508 22327261 10.1038/jid.2011.364

[embj2021108234-bib-0034] Haag SM , Gulen MF , Reymond L , Gibelin A , Abrami L , Decout A , Heymann M , van der Goot FG , Turcatti G , Behrendt R *et al* (2018) Targeting STING with covalent small‐molecule inhibitors. Nature 559: 269–273 29973723 10.1038/s41586-018-0287-8

[embj2021108234-bib-0035] Han J , Park SG , Bae JB , Choi J , Lyu JM , Park SH , Kim HS , Kim YJ , Kim S , Kim TY (2012) The characteristics of genome‐wide DNA methylation in naive CD4+ T cells of patients with psoriasis or atopic dermatitis. Biochem Biophys Res Comm 422: 157–163 22564738 10.1016/j.bbrc.2012.04.128

[embj2021108234-bib-0036] Hogg SJ , Beavis PA , Dawson MA , Johnstone RW 2020) Targeting the epigenetic regulation of antitumour immunity. Nature Reviews Drug Discov 19: 776–800 10.1038/s41573-020-0077-5 32929243

[embj2021108234-bib-0037] Hoong BYD , Gan YH , Liu H , Chen ES (2020) cGAS‐STING pathway in oncogenesis and cancer therapeutics. Oncotarget 11: 2930–2955 32774773 10.18632/oncotarget.27673 PMC7392626

[embj2021108234-bib-0038] Jackson‐Grusby L , Beard C , Possemato R , Tudor M , Fambrough D , Csankovszki G , Dausman J , Lee P , Wilson C , Lander E *et al* (2001) Loss of genomic methylation causes p53‐dependent apoptosis and epigenetic deregulation. Nat Genet 27: 31–39 11137995 10.1038/83730

[embj2021108234-bib-0039] Javierre BM , Hernando H , Ballestar E (2012) Environmental triggers and epigenetic deregulation in autoimmune disease. Discov Med 12: 535–545 22204770

[embj2021108234-bib-0040] Jiao H , Wachsmuth L , Kumari S , Schwarzer R , Lin J , Eren RO , Fisher A , Lane R , Young GR , Kassiotis G *et al* (2020) Z‐nucleic‐acid sensing triggers ZBP1‐dependent necroptosis and inflammation. Nature 580: 391–395 32296175 10.1038/s41586-020-2129-8 PMC7279955

[embj2021108234-bib-0041] Jones PA , Issa JP , Baylin S (2016) Targeting the cancer epigenome for therapy. Nat Rev Genet 17: 630–641 27629931 10.1038/nrg.2016.93

[embj2021108234-bib-0042] Jones PA , Ohtani H , Chakravarthy A , De Carvalho DD (2019) Epigenetic therapy in immune‐oncology. Nat Rev Cancer 19: 151–161 30723290 10.1038/s41568-019-0109-9

[embj2021108234-bib-0043] Kabashima K , Honda T , Ginhoux F , Egawa G (2019) The immunological anatomy of the skin. Nat Rev Immunol 19: 19–30 30429578 10.1038/s41577-018-0084-5

[embj2021108234-bib-0044] Karemaker ID , Baubec T (2020) DNA methyltransferases hitchhiking on chromatin. Swiss Med Wkly 150: w20329 32920789 10.4414/smw.2020.20329

[embj2021108234-bib-0045] Kawai T , Akira S (2011) Toll‐like receptors and their crosstalk with other innate receptors in infection and immunity. Immunity 34: 637–650 21616434 10.1016/j.immuni.2011.05.006

[embj2021108234-bib-0046] de Koning HD , Bergboer JG , van den Bogaard EH , van Vlijmen‐Willems IM , Rodijk‐Olthuis D , Simon A , Zeeuwen PL , Schalkwijk J (2012) Strong induction of AIM2 expression in human epidermis in acute and chronic inflammatory skin conditions. Exp Dermatol 21: 961–964 23171461 10.1111/exd.12037

[embj2021108234-bib-0047] Kwon J , Bakhoum SF (2020) The Cytosolic DNA‐Sensing cGAS‐STING Pathway in Cancer. Cancer Discov 10: 26–39 31852718 10.1158/2159-8290.CD-19-0761 PMC7151642

[embj2021108234-bib-0048] Lai J , Fu Y , Tian S , Huang S , Luo X , Lin L , Zhang X , Wang H , Lin Z , Zhao H *et al* (2021) Zebularine elevates STING expression and enhances cGAMP cancer immunotherapy in mice. Mol Ther 29: 1758–1771 33571681 10.1016/j.ymthe.2021.02.005 PMC8116609

[embj2021108234-bib-0049] Langmead B , Trapnell C , Pop M , Salzberg SL (2009) Ultrafast and memory‐efficient alignment of short DNA sequences to the human genome. Genome Biol 10: R25 19261174 10.1186/gb-2009-10-3-r25 PMC2690996

[embj2021108234-bib-0050] Lee PP , Fitzpatrick DR , Beard C , Jessup HK , Lehar S , Makar KW , Pérez‐Melgosa M , Sweetser MT , Schlissel MS , Nguyen S *et al* (2001) A critical role for Dnmt1 and DNA methylation in T cell development, function, and survival. Immunity 15: 763–774 11728338 10.1016/s1074-7613(01)00227-8

[embj2021108234-bib-0051] Leyvraz C , Charles RP , Rubera I , Guitard M , Rotman S , Breiden B , Sandhoff K , Hummler E (2005) The epidermal barrier function is dependent on the serine protease CAP1/Prss8. J Cell Biol 170: 487–496 16061697 10.1083/jcb.200501038 PMC2171460

[embj2021108234-bib-0052] Li E , Bestor TH , Jaenisch R (1992) Targeted mutation of the DNA methyltransferase gene results in embryonic lethality. Cell 69: 915–926 1606615 10.1016/0092-8674(92)90611-f

[embj2021108234-bib-0053] Li J , Jiang TX , Hughes MW , Wu P , Yu J , Widelitz RB , Fan G , Chuong CM (2012) Progressive alopecia reveals decreasing stem cell activation probability during aging of mice with epidermal deletion of DNA methyltransferase 1. J Invest Dermatol 132: 2681–2690 22763785 10.1038/jid.2012.206 PMC3465630

[embj2021108234-bib-0054] Li X , Zhang Q , Ding Y , Liu Y , Zhao D , Zhao K , Shen Q , Liu X , Zhu X , Li N *et al* (2016) Methyltransferase Dnmt3a upregulates HDAC9 to deacetylate the kinase TBK1 for activation of antiviral innate immunity. Nat Immunol 17: 806–815 27240213 10.1038/ni.3464

[embj2021108234-bib-0055] Licht JD (2015) DNA methylation inhibitors in cancer therapy: the immunity dimension. Cell 162: 938–939 26317460 10.1016/j.cell.2015.08.005

[embj2021108234-bib-0056] Love MI , Huber W , Anders S (2014) Moderated estimation of fold change and dispersion for RNA‐seq data with DESeq2. Genome Biol 15: 550 25516281 10.1186/s13059-014-0550-8 PMC4302049

[embj2021108234-bib-0057] Lutsik P , Feuerbach L , Arand J , Lengauer T , Walter J , Bock C (2011) BiQ Analyzer HT: locus‐specific analysis of DNA methylation by high‐throughput bisulfite sequencing. Nucleic Acids Res 39: W551–W556 21565797 10.1093/nar/gkr312 PMC3125748

[embj2021108234-bib-0058] Ma F , Li B , Liu SY , Iyer SS , Yu Y , Wu A , Cheng G (2015) Positive feedback regulation of type I IFN production by the IFN‐inducible DNA sensor cGAS. J Immunol 194: 1545–1554 25609843 10.4049/jimmunol.1402066 PMC4324085

[embj2021108234-bib-0059] Mackenzie KJ , Carroll P , Martin C‐A , Murina O , Fluteau A , Simpson DJ , Olova N , Sutcliffe H , Rainger JK , Leitch A *et al* (2017) cGAS surveillance of micronuclei links genome instability to innate immunity. Nature 548: 461–465 28738408 10.1038/nature23449 PMC5870830

[embj2021108234-bib-0060] Magoc T , Salzberg SL (2011) FLASH: fast length adjustment of short reads to improve genome assemblies. Bioinformatics 27: 2957–2963 21903629 10.1093/bioinformatics/btr507 PMC3198573

[embj2021108234-bib-0061] Mannion N , Greenwood SM , Young R , Cox S , Brindle J , Read D , Nellåker C , Vesely C , Ponting C , McLaughlin P *et al* (2014) The RNA‐editing enzyme ADAR1 controls innate immune responses to RNA. Cell Rep 9: 1482–1494 25456137 10.1016/j.celrep.2014.10.041 PMC4542304

[embj2021108234-bib-0062] Martin M (2011) Cutadapt removes adapter sequences from high‐throughput sequencing reads. EMBnet.journal 17: 10

[embj2021108234-bib-0063] McLaughlin LJ , Stojanovic L , Kogan AA , Rutherford JL , Choi EY , Yen R‐W , Xia L , Zou Y , Lapidus RG , Baylin SB *et al* (2020) Pharmacologic induction of innate immune signaling directly drives homologous recombination deficiency. Proc Natl Acad Sci USA 117: 17785–17795 32651270 10.1073/pnas.2003499117 PMC7395437

[embj2021108234-bib-0064] Mehdipour P , Marhon SA , Ettayebi I , Chakravarthy A , Hosseini A , Wang Y , de Castro FA , Loo Yau H , Ishak C , Abelson S *et al* (2020) Epigenetic therapy induces transcription of inverted SINEs and ADAR1 dependency. Nature 588: 169–173 33087935 10.1038/s41586-020-2844-1

[embj2021108234-bib-0065] Melki I , Fremond ML (2020) Type I Interferonopathies: from a Novel Concept to Targeted Therapeutics. Curr Rheumatol Rep 22: 32 32548765 10.1007/s11926-020-00909-4

[embj2021108234-bib-0066] Mikkola ML (2007) Genetic basis of skin appendage development. Semin Cell Dev Biol 18: 225–236 17317239 10.1016/j.semcdb.2007.01.007

[embj2021108234-bib-0067] Miroshnikova YA , Cohen I , Ezhkova E , Wickstrom SA (2019) Epigenetic gene regulation, chromatin structure, and force‐induced chromatin remodelling in epidermal development and homeostasis. Curr Opin Genet Dev 55: 46–51 31112907 10.1016/j.gde.2019.04.014 PMC8259782

[embj2021108234-bib-0068] Mohan KN , Chaillet JR (2013) Cell and molecular biology of DNA methyltransferase 1. Int Rev Cell Mol Biol 306: 1–42 24016522 10.1016/B978-0-12-407694-5.00001-8

[embj2021108234-bib-0069] Morita M , Stamp G , Robins P , Dulic A , Rosewell I , Hrivnak G , Daly G , Lindahl T , Barnes DE (2004) Gene‐targeted mice lacking the Trex1 (DNase III) 3'–>5' DNA exonuclease develop inflammatory myocarditis. Mol Cell Biol 24: 6719–6727 15254239 10.1128/MCB.24.15.6719-6727.2004 PMC444847

[embj2021108234-bib-0070] Mortazavi A , Williams BA , McCue K , Schaeffer L , Wold B (2008) Mapping and quantifying mammalian transcriptomes by RNA‐Seq. Nat Methods 5: 621–628 18516045 10.1038/nmeth.1226 PMC13303166

[embj2021108234-bib-0071] Nguyen AV , Soulika AM (2019) The dynamics of the Skin's immune system. Int J Mol Sci 20: 1811 10.3390/ijms20081811 PMC6515324 31013709

[embj2021108234-bib-0072] Ramirez A , Page A , Gandarillas A , Zanet J , Pibre S , Vidal M , Tusell L , Genesca A , Whitaker DA , Melton DW *et al* (2004) A keratin K5Cre transgenic line appropriate for tissue‐specific or generalized Cre‐mediated recombination. Genesis 39: 52–57 15124227 10.1002/gene.20025

[embj2021108234-bib-0073] Ramsahoye BH (2002) Measurement of genome‐wide DNA cytosine‐5 methylation by reversed‐phase high‐pressure liquid chromatography. Methods Mol Biol 200: 17–27 11951652 10.1385/1-59259-182-5:017

[embj2021108234-bib-0074] Rigby RE , Webb LM , Mackenzie KJ , Li Y , Leitch A , Reijns MAM , Lundie RJ , Revuelta A , Davidson DJ , Diebold S *et al* (2014) RNA:DNA hybrids are a novel molecular pattern sensed by TLR9. EMBO J 33: 542–558 24514026 10.1002/embj.201386117 PMC3989650

[embj2021108234-bib-0075] Robinson MD , McCarthy DJ , Smyth GK (2010) edgeR: a Bioconductor package for differential expression analysis of digital gene expression data. Bioinformatics 26: 139–140 19910308 10.1093/bioinformatics/btp616 PMC2796818

[embj2021108234-bib-0076] Rodríguez E , Baurecht H , Wahn AF , Kretschmer A , Hotze M , Zeilinger S , Klopp N , Illig T , Schramm K , Prokisch H *et al* (2014) An integrated epigenetic and transcriptomic analysis reveals distinct tissue‐specific patterns of DNA methylation associated with atopic dermatitis. J Invest Dermatol 134: 1873–1883 24739813 10.1038/jid.2014.87

[embj2021108234-bib-0077] Roers A , Hiller B , Hornung V (2016) Recognition of endogenous nucleic acids by the innate immune system. Immunity 44: 739–754 27096317 10.1016/j.immuni.2016.04.002

[embj2021108234-bib-0078] Roulois D , Loo Yau H , Singhania R , Wang Y , Danesh A , Shen S , Han H , Liang G , Jones P , Pugh T *et al* (2015) DNA‐demethylating agents target colorectal cancer cells by inducing viral mimicry by endogenous transcripts. Cell 162: 961–973 26317465 10.1016/j.cell.2015.07.056 PMC4843502

[embj2021108234-bib-0079] Schindelin J , Arganda‐Carreras I , Frise E , Kaynig V , Longair M , Pietzsch T , Preibisch S , Rueden C , Saalfeld S , Schmid B *et al* (2012) Fiji: an open‐source platform for biological‐image analysis. Nat Methods 9: 676–682 22743772 10.1038/nmeth.2019 PMC3855844

[embj2021108234-bib-0080] Schlee M , Hartmann G (2016) Discriminating self from non‐self in nucleic acid sensing. Nat Rev Immunol 16: 566–580 27455396 10.1038/nri.2016.78 PMC7097691

[embj2021108234-bib-0081] Schoggins JW , MacDuff DA , Imanaka N , Gainey MD , Shrestha B , Eitson JL , Mar KB , Richardson RB , Ratushny AV , Litvak V *et al* (2014) Pan‐viral specificity of IFN‐induced genes reveals new roles for cGAS in innate immunity. Nature 505: 691–695 24284630 10.1038/nature12862 PMC4077721

[embj2021108234-bib-0082] Schubeler D (2015) Function and information content of DNA methylation. Nature 517: 321–326 25592537 10.1038/nature14192

[embj2021108234-bib-0083] Sen GL , Reuter JA , Webster DE , Zhu L , Khavari PA (2010) DNMT1 maintains progenitor function in self‐renewing somatic tissue. Nature 463: 563–567 20081831 10.1038/nature08683 PMC3050546

[embj2021108234-bib-0084] Sheaffer KL , Kim R , Aoki R , Elliott EN , Schug J , Burger L , Schubeler D , Kaestner KH (2014) DNA methylation is required for the control of stem cell differentiation in the small intestine. Genes Dev 28: 652–664 24637118 10.1101/gad.230318.113 PMC3967052

[embj2021108234-bib-0085] Sibilia M , Fleischmann A , Behrens A , Stingl L , Carroll J , Watt FM , Schlessinger J , Wagner EF (2000) The EGF receptor provides an essential survival signal for SOS‐dependent skin tumor development. Cell 102: 211–220 10943841 10.1016/s0092-8674(00)00026-x

[embj2021108234-bib-0086] Simon M , Van Meter M , Ablaeva J , Ke Z , Gonzalez RS , Taguchi T , De Cecco M , Leonova KI , Kogan V , Helfand SL *et al* (2019) LINE1 derepression in aged wild‐type and SIRT6‐deficient mice drives inflammation. Cell Metab 29: 871–885.e5 30853213 10.1016/j.cmet.2019.02.014 PMC6449196

[embj2021108234-bib-0087] Smith ZD , Meissner A (2013) DNA methylation: roles in mammalian development. Nat Rev Genet 14: 204–220 23400093 10.1038/nrg3354

[embj2021108234-bib-0088] Stopper H , Korber C , Schiffmann D , Caspary WJ (1993) Cell‐cycle dependent micronucleus formation and mitotic disturbances induced by 5‐azacytidine in mammalian cells. Mutat Res 300: 165–177 7687016 10.1016/0165-1218(93)90048-i

[embj2021108234-bib-0089] Stresemann C , Lyko F (2008) Modes of action of the DNA methyltransferase inhibitors azacytidine and decitabine. Int J Cancer 123: 8–13 18425818 10.1002/ijc.23607

[embj2021108234-bib-0090] Sun Q , Sun L , Liu HH , Chen X , Seth RB , Forman J , Chen ZJ (2006) The specific and essential role of MAVS in antiviral innate immune responses. Immunity 24: 633–642 16713980 10.1016/j.immuni.2006.04.004

[embj2021108234-bib-0091] Swanson KV , Junkins RD , Kurkjian CJ , Holley‐Guthrie E , Pendse AA , El Morabiti R , Petrucelli A , Barber GN , Benedict CA , Ting JP (2017) A noncanonical function of cGAMP in inflammasome priming and activation. J Exp Med 214: 3611–3626 29030458 10.1084/jem.20171749 PMC5716045

[embj2021108234-bib-0092] Tsumura A , Hayakawa T , Kumaki Y , Takebayashi S‐I , Sakaue M , Matsuoka C , Shimotohno K , Ishikawa F , Li EN , Ueda HR *et al* (2006) Maintenance of self‐renewal ability of mouse embryonic stem cells in the absence of DNA methyltransferases Dnmt1, Dnmt3a and Dnmt3b. Genes Cells 11: 805–814 16824199 10.1111/j.1365-2443.2006.00984.x

[embj2021108234-bib-0093] Uggenti C , Lepelley A , Crow YJ (2019) Self‐awareness: nucleic acid‐driven inflammation and the type I interferonopathies. Annu Rev Immunol 37: 247–267 30633609 10.1146/annurev-immunol-042718-041257

[embj2021108234-bib-0094] Wagner T , Gschwandtner M , Strajeriu A , Elbe‐Burger A , Grillari J , Grillari‐Voglauer R , Greiner G , Golabi B , Tschachler E , Mildner M (2018) Establishment of keratinocyte cell lines from human hair follicles. Sci Rep 8: 13434 30194332 10.1038/s41598-018-31829-0 PMC6128885

[embj2021108234-bib-0095] Winter M , Moser MA , Meunier D , Fischer C , Machat G , Mattes K , Lichtenberger BM , Brunmeir R , Weissmann S , Murko C *et al* (2013) Divergent roles of HDAC1 and HDAC2 in the regulation of epidermal development and tumorigenesis. EMBO J 32: 3176–3191 24240174 10.1038/emboj.2013.243 PMC3981143

[embj2021108234-bib-0096] Xu GL , Bestor TH , Bourc'his D , Hsieh CL , Tommerup N , Bugge M , Hulten M , Qu X , Russo JJ , Viegas‐Pequignot E (1999) Chromosome instability and immunodeficiency syndrome caused by mutations in a DNA methyltransferase gene. Nature 402: 187–191 10647011 10.1038/46052

[embj2021108234-bib-0097] Yum S , Li M , Chen ZJ (2020) Old dogs, new trick: classic cancer therapies activate cGAS. Cell Res 30: 639–648 32541866 10.1038/s41422-020-0346-1 PMC7395767

[embj2021108234-bib-0098] Zheng Y , Danilenko DM , Valdez P , Kasman I , Eastham‐Anderson J , Wu J , Ouyang W (2007) Interleukin‐22, a T(H)17 cytokine, mediates IL‐23‐induced dermal inflammation and acanthosis. Nature 445: 648–651 17187052 10.1038/nature05505

[embj2021108234-bib-0099] Zupkovitz G , Kabiljo J , Kothmayer M , Schlick K , Schofer C , Lagger S , Pusch O (2021) Analysis of methylation dynamics reveals a tissue‐specific, age‐dependent decline in 5‐methylcytosine within the genome of the vertebrate aging model Nothobranchius furzeri. Front Mol Biosci 8: 627143 34222326 10.3389/fmolb.2021.627143 PMC8242171

