# Supplementary material for: DNA hypomethylation leads to cGAS‐induced autoinflammation in the epidermis
Source: EMBO J. 2021 Sep 29;40(22):e108234. doi: 10.15252/embj.2021108234 (PMC8591534; doi:10.15252/embj.2021108234)
Supplement: Supplementary file 1 — Appendix [file EMBJ-40-e108234-s004.pdf]

Appendix

DNA hypomethylation leads to cGAS-induced autoinflammation in the epidermis

Table of content

Appendix Figure S1: Newborn *Dnmt1*$^{\Delta/\Delta ep}$ mice show normal formation of the skin barrier and unchanged epidermal differentiation………….……………………………………………………..2

Appendix Figure S2: Increased proliferation, apoptosis and DNA damage in the epidermis of *Dnmt1*$^{\Delta/\Delta ep}$ mice ……………………………..………………………………………………….4

Appendix Figure S3: *Dnmt1*$^{\Delta/\Delta ep}$ *Mavs*$^{-/-}$ mice do not show improvement in survival, histopathology or immune cell infiltration……………………………………………………………6

Appendix Figure S4: Detailed results of the dermatohistopathological parameters analyzed in control, *Dnmt1*$^{\Delta/\Delta ep}$, *Dnmt1*$^{\Delta/\Delta ep}$ *Cgas*$^{-/-}$ and *Dnmt1*$^{\Delta/\Delta ep}$ *Mavs*$^{-/-}$ mice…………………………….8

Appendix Table S1: Histopathological score sheet for evaluation of the effects of epidermal *Dnmt1* deletion……………………………………………………………………………….…10

Appendix Table S2: Bisulfite Amplicon sequencing of selected repeats…..........................…10

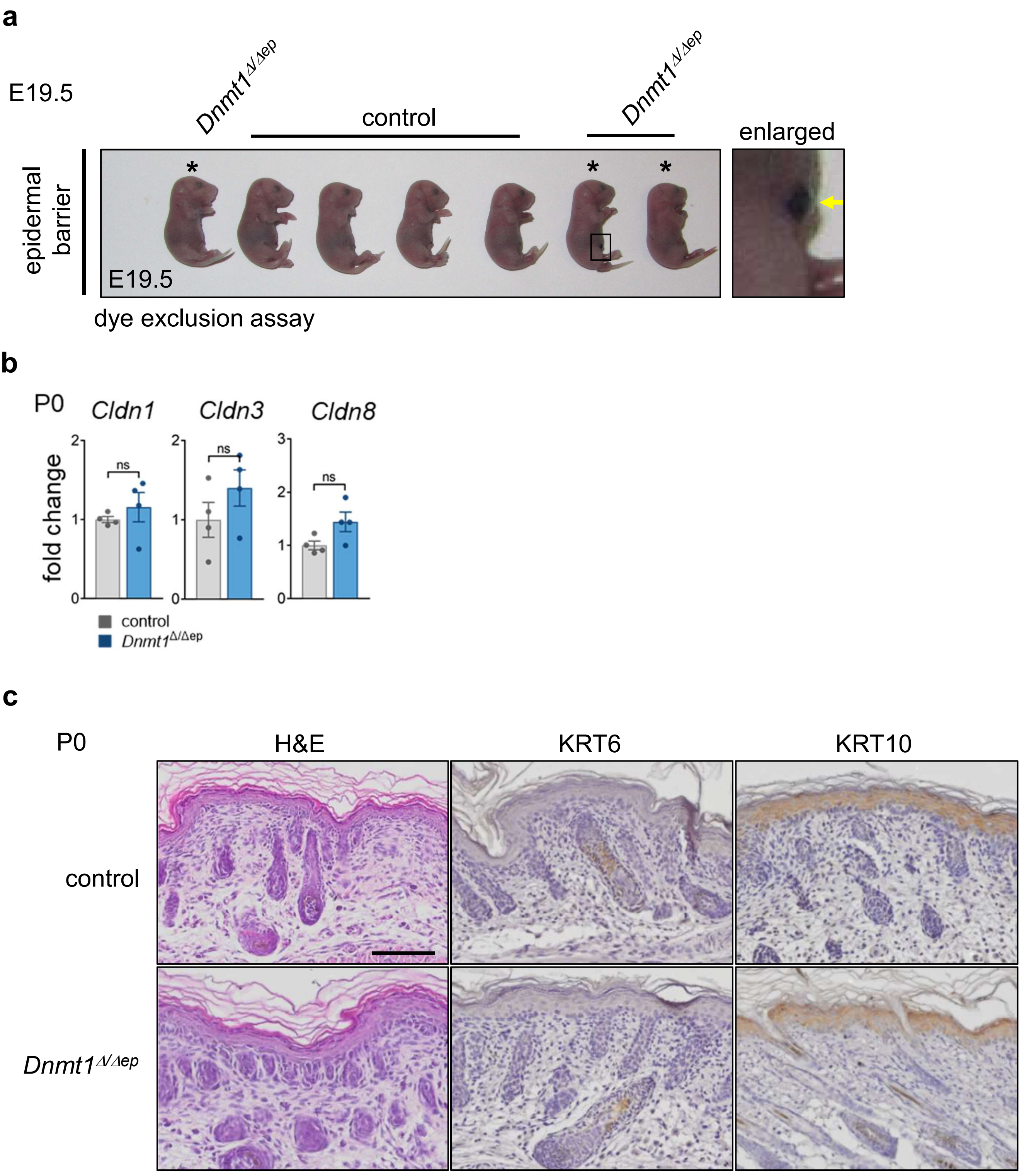

Appendix Figure S1

Appendix Figure S1: Newborn *Dnmt1*$^{\Delta/\Delta ep}$ mice show normal formation of the skin barrier and unchanged epidermal differentiation

(**a**) Dye exclusion assay by toluidine blue staining of control and *Dnmt1*$^{\Delta/\Delta ep}$ mice (indicated by an asterisk) at E19.5. The stained umbilicus serves as a positive control for successful staining, which is indicated by an arrow in the enlarged image.

(**b**) Comparison of expression levels of tight junction associated genes of P0 of control (n=4) and *Dnmt1*$^{\Delta/\Delta ep}$ (n=4) mice using two-tailed t-test. Data are mean ± SEM. ns not significant.

(**c**) Dorsal skin sections of P0 of control and *Dnmt1*$^{\Delta/\Delta ep}$ mice, which were stained for H&E or immunolabeled for KERATIN 6 (KRT6) or KERATIN 10 (KRT10). Data information: Scale bar, 100 µm.

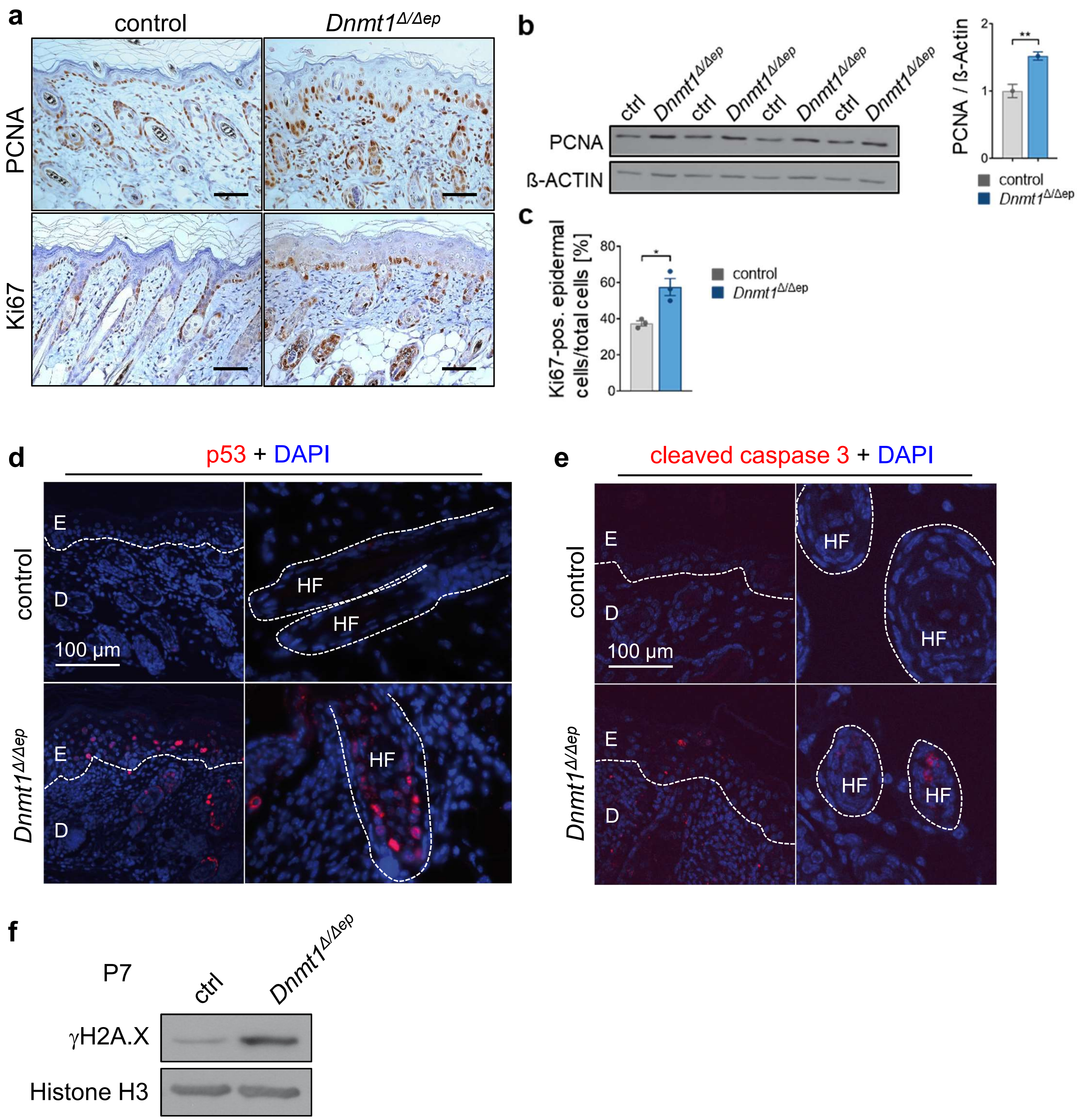

Appendix Figure S2

Appendix Figure S2: Increased proliferation, apoptosis and DNA damage in the epidermis of *Dnmt1*$^{\Delta/\Delta ep}$ mice

(**a**) Immunolabeling of dorsal skin sections obtained from control and *Dnmt1*$^{\Delta/\Delta ep}$ (P5) mice for PCNA and Ki67 counterstained with Hematoxylin. Data information: Scale bar, 100 µm.

(**b)** Immunoblot analysis for PCNA of P5 epidermal protein extracts from control and *Dnmt1*$^{\Delta/\Delta ep}$ epidermis. β-actin was used as loading control. Relative intensities of signals were quantified by densitometric scanning and are shown relative to β-actin (right panel). Data are mean ± SEM and compared using two-tailed t-test ** p≤0.01.n=4 mice per group.

(**c**) The number of Ki67-positive cells in the epidermis of control and *Dnmt1*$^{\Delta/\Delta ep}$ mice were compared and calculated relative to the total number of epidermal cells using two-tailed students t-test. Data are mean ± SEM. * p≤0.05 n=3.

(**d, e**) IF staining of dorsal skin sections obtained from P7 control and *Dnmt1*$^{\Delta/\Delta ep}$ mice for p53 (**d**) and active caspase 3 (**e**). Dashed lines indicate dermal-epidermal border or the edge of hair follicles .

(**f**) Immunoblot analyses of P7 epidermal protein extracts from control and *Dnmt1*$^{\Delta/\Delta ep}$ epidermis with antibodies specific for γH2A.X and for the loading control histone H3.

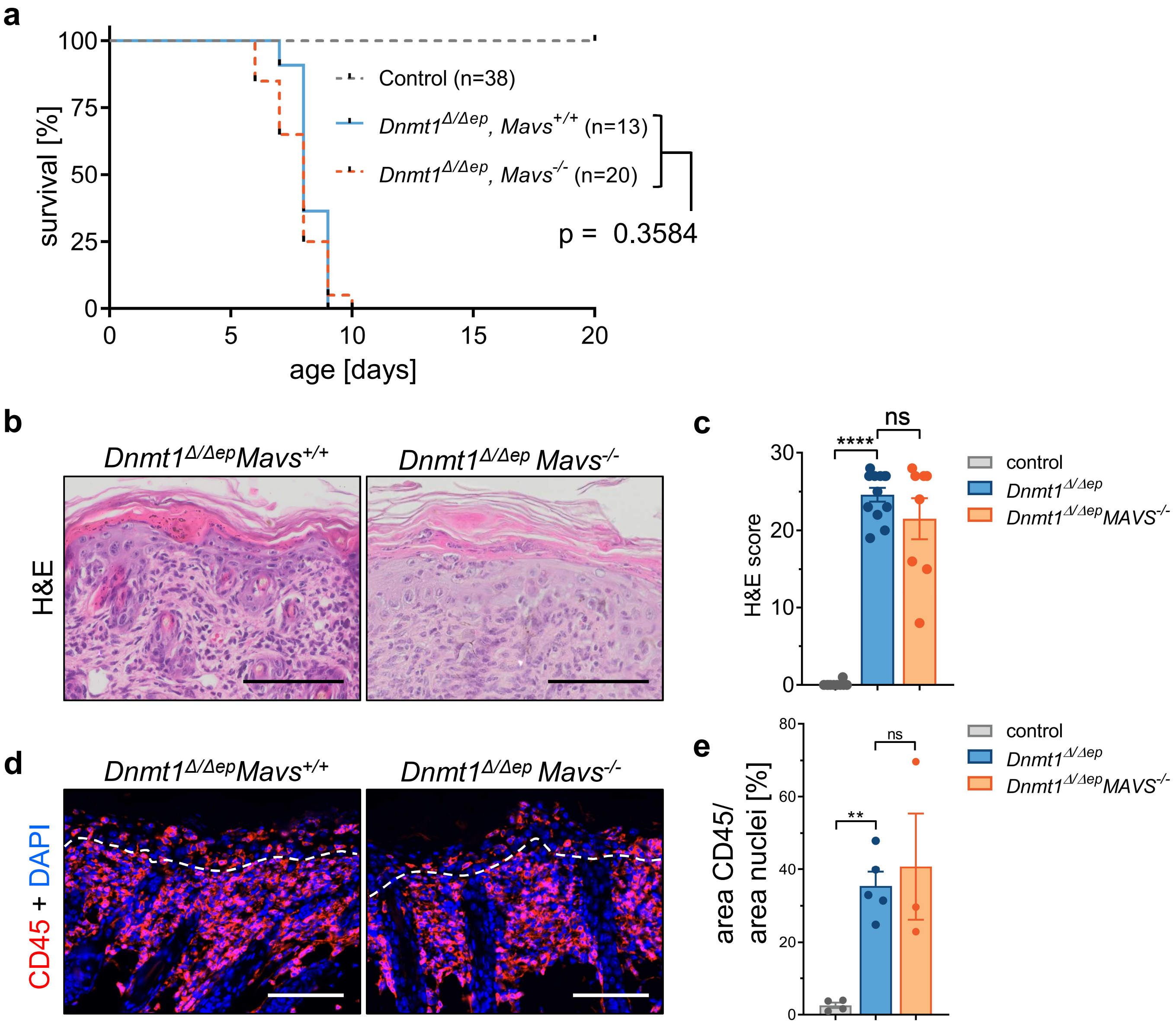

Appendix Figure S3

Appendix Figure S3: *Dnmt1$^{\Delta/\Delta ep}$ Mavs$^{-/-}$* mice do not show improvement in survival, histopathology or immune cell infiltration

(**a**) Kaplan-Meier plot showing survival of control (n=38), *Dnmt1$^{\Delta/\Delta ep}$ Mavs$^{+/+}$* (n=13) and *Dnmt1$^{\Delta/\Delta ep}$ Mavs$^{-/-}$* mice (n=20). No statistical differences was detected when *Dnmt1$^{\Delta/\Delta ep}$ Mavs$^{+/+}$* and *Dnmt1$^{\Delta/\Delta ep}$ Mavs$^{-/-}$* mice were compared by log rank test (Mantel Cox), P=0.3584.

(**b**) Representative images of H&E staining of dorsal skin sections from *Dnmt1$^{\Delta/\Delta ep}$* and *Dnmt1$^{\Delta/\Delta ep}$ Mavs$^{-/-}$* mice from P7.

(**c**) Histoscoring of P7 H&E dorsal skin sections from control, *Dnmt1$^{\Delta/\Delta ep}$Mavs$^{+/+}$* and *Dnmt1$^{\Delta/\Delta ep}$Mavs$^{-/-}$* mice was performed using different histopathological parameters (see **Appendix Figure S4**) of at least 8 animals per genotype that were assessed in a blinded manner. Comparison was performed using one way-ANOVA with post hoc Holm-Sidak multiple comparison test. Data are mean ± SEM. ns not significant, **** p≤0.0001.

(**d**) Representative images of CD45 labeling of dorsal skin sections from control, *Dnmt1$^{\Delta/\Delta ep}$Mavs$^{+/+}$* and *Dnmt1$^{\Delta/\Delta ep}$Mavs$^{-/-}$* mice from P7. Dashed lines indicate the dermal-epidermal border.

(**e**) Quantification of CD45-positve cells obtained from control (n=4), *Dnmt1$^{\Delta/\Delta ep}$ Mavs$^{+/+}$* (n=4) and *Dnmt1$^{\Delta/\Delta ep}$ Mavs$^{-/-}$* (n=3) mice. Tissue sections approximately 1 cm in length were scanned and the area of CD45 positive cells per area of nuclei was quantified using ImageJ. Statistical analysis was done using one way-ANOVA with post hoc Holm-Sidak multiple comparison test. Data are mean ± SEM. ns not significant, ** p≤0.05.

Data information: Scale bars, 100 µm.

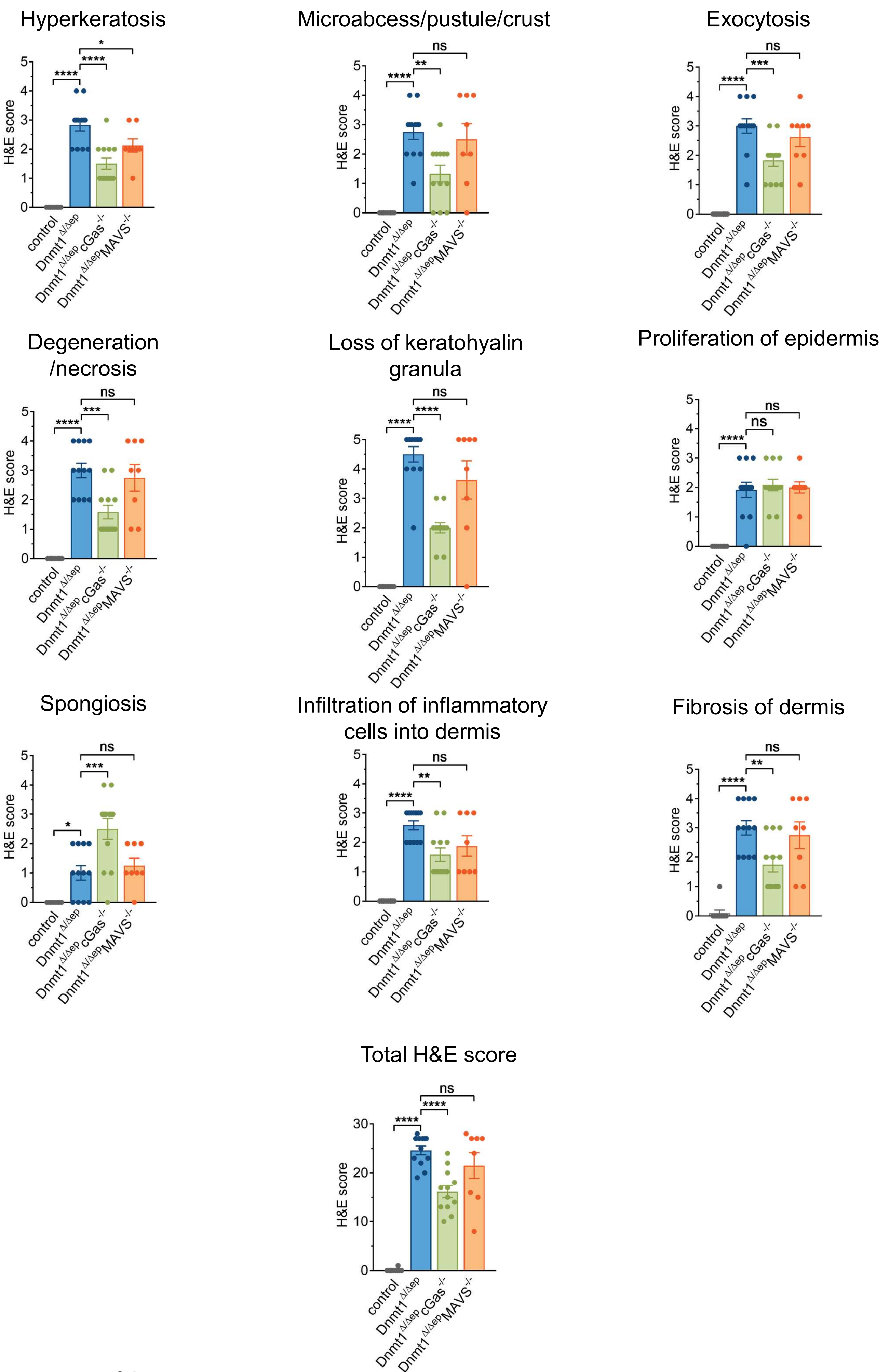

Appendix Figure S4

Appendix Figure S4: Detailed results of the dermatohistopathological parameters analyzed in control, *Dnmt1$^{Δ/Δep}$*, *Dnmt1$^{Δ/Δep}$ Cgas$^{-/-}$* and *Dnmt1$^{Δ/Δep}$ Mavs$^{-/-}$* mice

Histopathological analysis of skin from 7 days old control, *Dnmt1$^{Δ/Δep}$*, *Dnmt1$^{Δ/Δep}$ Mavs$^{-/-}$* and *Dnmt1$^{Δ/Δep}$ Cgas$^{-/-}$* mice (n≥8, each). Coded slides were scanned and analyzed in a blinded manner for several histopathological parameters (See **Appendix Table S1)**. The different parameters are scored in a range of from 0 (not present) to 5 (massive) by the pathologist in a blinded histopathological analysis. Total H&E score was deduced from single histopathological parameters. For every parameter genotyped were compared using one way-ANOVA with (post hoc Tukey multiple comparison test. ns not significant, Data are mean ± SEM. ns not significant,* p≤0.05, ** p≤0.01, *** p≤0.001, **** p≤0.0001.

Appendix Table S1: Histopathological score sheet for evaluation of the effects of epidermal *Dnmt1* deletion

**Hyperkeratosis**	**Inflammatory cell infiltrate into the epidermis or dermis**	**Spongiosis**	**Proliferation of epidermis**	**Loss of keratohyalin granula**	**Degeneration/necrosis of the epidermis**	**Exocytosis**	**Fibrosis of the dermis**
0 = none	0 = none	0 = none	0 = none	0 = not present	0 = none	0 = none	0 = none
1 = minimal	1 = minimal	1 = minimal	1 = minimal	1 = 1 – 20 %	1 = single cell necrosis	1 = minimal	1 = minimal
2 = marked	2 = marked	2 = marked	2 = marked	2 = 20 – 40 %	2 = Multiple foci affected	2 = marked	2 = marked
3 = moderate	3 = moderate	3 = moderate	3 = moderate	3 = 40 – 60 %	3 = Numerous foci / few erosions	3 = moderate	3 = moderate
4 = severe	4 = severe	4 = severe	4 = severe	4 = 60 – 80 %	4 = Multiple erosions and /or ulceration	4 = severe	4 = severe
5 = massive	5 = massive	5 = massive	5 = massive	5 = 80 – 100%	5 = multifocal extensive ulceration	5 = massive	5 = massive

Appendix Table S2: Bisulfite Amplicon sequencing of selected repeats

Samples	IAP-LTR1a		major Satellites		
	reads analyzed	% mCpG	reads analyzed	% mCpG	% conversion HP linker
control1	3106	94	1361	88.04	99.25
control2	7703	93.4	1777	83.38	99.56
control3	5573	94.4	1718	83.52	99.50
Dnmt1$^{\Delta/\Delta ep}$1	4390	45.6	3918	44.98	99.41
Dnmt1$^{\Delta/\Delta ep}$2	10402	43.8	1887	42.6	99.54
Dnmt1$^{\Delta/\Delta ep}$3	5732	50.6	1350	45.03	99.70
