# Supplementary material for: DNA hypomethylation leads to cGAS‐induced autoinflammation in the epidermis
Source: EMBO J. 2021 Sep 29;40(22):e108234. doi: 10.15252/embj.2021108234 (PMC8591534; doi:10.15252/embj.2021108234)
Supplement: Supplementary file 2 — Expanded View Figures PDF [file EMBJ-40-e108234-s001.pdf]

Colour online

Expanded View Figures

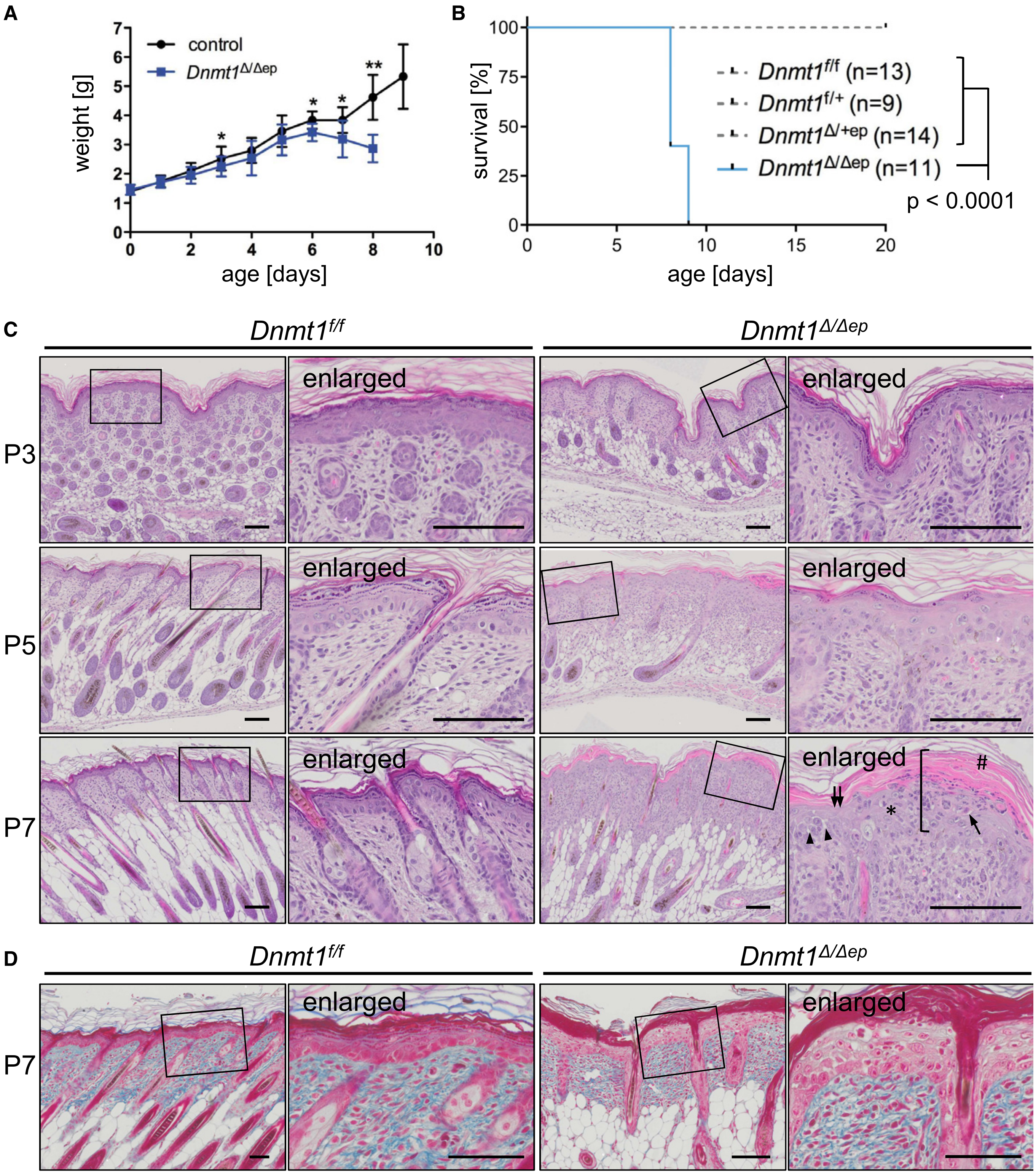

Figure EV1.

© 2021 The Authors The EMBO Journal 40: e108234 | 2021 19 of 18

Figure EV1. Keratinocyte-specific deletion of *Dnmt1* in mice results in early postnatal lethality associated with severe dermatopathologic alterations.

A Weight curve of control (*Dnmt1*$^{f/f}$) and *Dnmt1*$^{\Delta/\Delta ep}$ mice from P0-P10. Control: P0 $n = 2$; P1 $n = 25$; P2 $n = 44$; P3 $n = 46$; P4 $n = 17$; P5 $n = 23$; P6 $n = 12$; P7 $n = 19$; P8 $n = 14$; P9 $n = 18$. *Dnmt1*$^{\Delta/\Delta ep}$ mice: P0 $n = 3$; P1 $n = 9$; P2 $n = 13$; P3 $n = 14$; P4 $n = 7$; P5 $n = 6$; P6 $n = 7$; P7 $n = 4$; P8 $n = 3$; P9 all died. Data are mean ± SEM and compared using two-tailed *t*-test. *$P \leq 0.05$, **$P \leq 0.01$.

B Kaplan–Meier plot comparing control (*Dnmt1*$^{f/f}$ $n = 13$, *Dnmt1*$^{f/+}$ $n = 9$, *Dnmt1*$^{\Delta/+ep}$ $n = 14$) and *Dnmt1*$^{\Delta/\Delta ep}$ ($n = 11$) mice using log-rank test (Mantel–Cox). $P < 0.0001$.

C H&E staining of dorsal skin sections from control and *Dnmt1*$^{\Delta/\Delta ep}$ mice at P3, P5, and P7. Enlarged sections are indicated on the right. Dermatopathologic alterations found in *Dnmt1*$^{\Delta/\Delta ep}$ mice at P7 are indicated, such as acanthosis (bracket), hyperkeratosis (hashtag), loss of keratohyalin granules (double arrow), spongiosis (arrowheads), necrotic cells (arrow), and exocytosis (asterisk).

D Azan staining of dorsal skin section at P7. Enlarged sections are indicated on the right.

Data information: Scale bars, 50 μm (D), 100 μm (C).

Figure EV2. Epidermal DNA hypomethylation results in induced immune response and in systemic effects.

A Immunolabeling of dorsal skin sections from control and *Dnmt1*$^{\Delta/\Delta ep}$ mice for CD45. Dashed lines indicate dermal-epidermal border or the edge of hair follicles.

B Flow cytometric analysis of CD45-positive cells and the respective immune cell populations in the dermis of control and *Dnmt1*$^{\Delta/\Delta ep}$ mice of different postnatal ages. Cells are shown as percent of viable cells. Data are mean ± SD, two-tailed *t*-test. ns, not significant, *$P \leq 0.05$, **$P \leq 0.01$, CD45, P3-P7, $n \geq 4$ mice. All other markers: $n = 3$ (P3), 4 (P5), and 4 (P6 + P7).

C–E Comparison of *Dnmt1*$^{\Delta/\Delta ep}$ and control mice regarding blood sugar level (C, $n = 4$), protein concentration of urine (D, $n = 6$), and organ weight (E, $n \geq 4$).

F The inside out barrier of the skin of 3- to 7-day-old *Dnmt1*$^{\Delta/\Delta ep}$ and control pubs was tested by a dehydration assay, $n = 5$ (P3), $n = 11$ (P5, control), $n = 6$ (P5, *Dnmt1*$^{\Delta/\Delta ep}$), $n = 3$ (P7, control), and $n = 4$ (P7, *Dnmt1*$^{\Delta/\Delta ep}$). Extent of fluids loss was calculated by measuring the decrease of the weight during a certain time period and calculated as percent of body weight.

Data information: For analysis of (C–F), two-tailed Student's *t*-test was used. Data are mean ± SEM. ns, not significant, *$P \leq 0.05$, **$P \leq 0.01$, ***$P \leq 0.001$, ****$P \leq 0.0001$. Scale bars, 200 μm.

 © 2021 The Authors

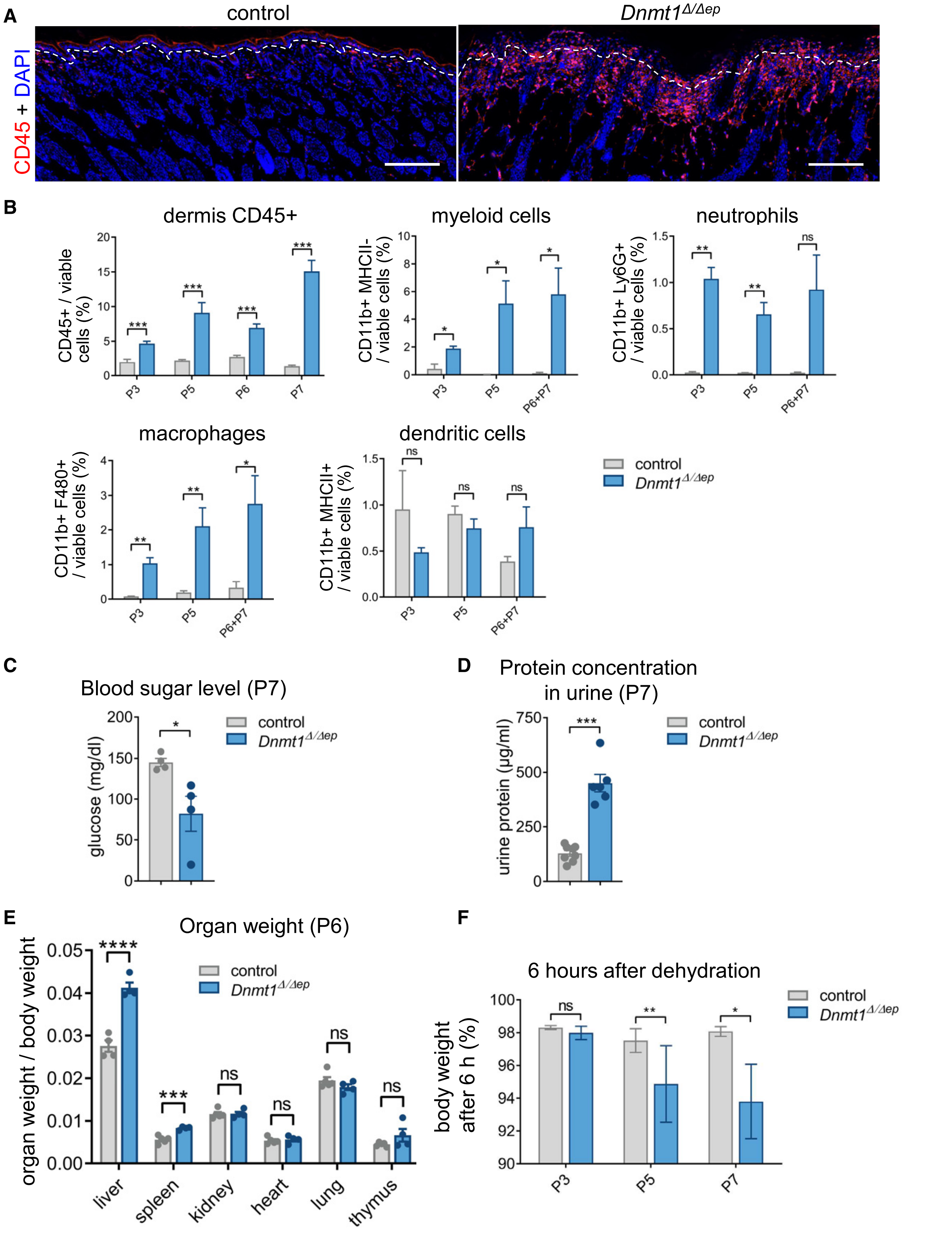
A
control
Dnmt1Δ/Δep
CD45 + DAPI
B
dermis CD45+
CD45+ / viable cells (%)
P3 P5 P6 P7
myeloid cells
CD11b+ MHCII- / viable cells (%)
P3 P5 P6+P7
neutrophils
CD11b+ Ly6G+ / viable cells (%)
macrophages
CD11b+ F480+ / viable cells (%)
dendritic cells
CD11b+ MHCII+ / viable cells (%)
ns
control
Dnmt1Δ/Δep
C
Blood sugar level (P7)
glucose (mg/dl)
D
Protein concentration in urine (P7)
urine protein (µg/ml)
E
Organ weight (P6)
organ weight / body weight
liver spleen kidney heart lung thymus
F
6 hours after dehydration
body weight after 6 h (%)
P3 P5 P7

Figure EV2.

Figure EV3. Keratinocytes of *Dnmt1*$^{\Delta/\Delta ep}$ mice exhibit DNA hypomethylation and derepression of transposon/repeat families.

A DNA methylation profile of wild-type keratinocytes at selected upregulated genes upon knockout of *Dnmt1*. DNA Methylation data were obtained from Chatterjee *et al*, 2014 and visualized in the IGV browser. The proximal promotor regions show lower methylation levels than surrounding CpGs.

B Expression of deregulated genes found by RNA sequencing of cultured primary keratinocytes that were isolated at P3 of control and *Dnmt1*$^{\Delta/\Delta ep}$ mice (n = 5-7). Statistical analyses were done using two-tailed Student's t-test. Data are mean ± SEM. ***$P \leq 0.001$, ****$P \leq 0.0001$.

C Changes in expression of transposon/repeat families were plotted in a scatter plot of log fold change versus log10(adjusted P-values).

D Relative mRNA expression of transposable elements in the epidermis of control and *Dnmt1*$^{\Delta/\Delta ep}$ mice at P3 (n = 3), P5 (n = 5), and P7 (n = 3). Statistical analyses were done using one-way ANOVA (Kruskal–Wallis test of multiple comparisons). Data are mean ± SEM. ns, not significant, **$P \leq 0.01$.

E, F DNA methylation of CD45-depleted epidermal keratinocytes isolated from newborn wild-type (n = 3) and *Dnmt1*$^{\Delta/\Delta ep}$ (n = 3) mice. Methylation status of repetitive elements was analyzed using deep amplicon bisulfite sequencing (IAP-LTR1a) or deep hairpin-bisulfite sequencing (major Satellites, mSat1). Mean values of DNA methylation of wild-type and *Dnmt1*$^{\Delta/\Delta ep}$ mice are shown in (F), and statistical analysis was done using two-tailed Student's t-test (see also Supp. Tab. S4). Data are mean ± SEM. **$P \leq 0.01$, ***$P \leq 0.001$.

 © 2021 The Authors

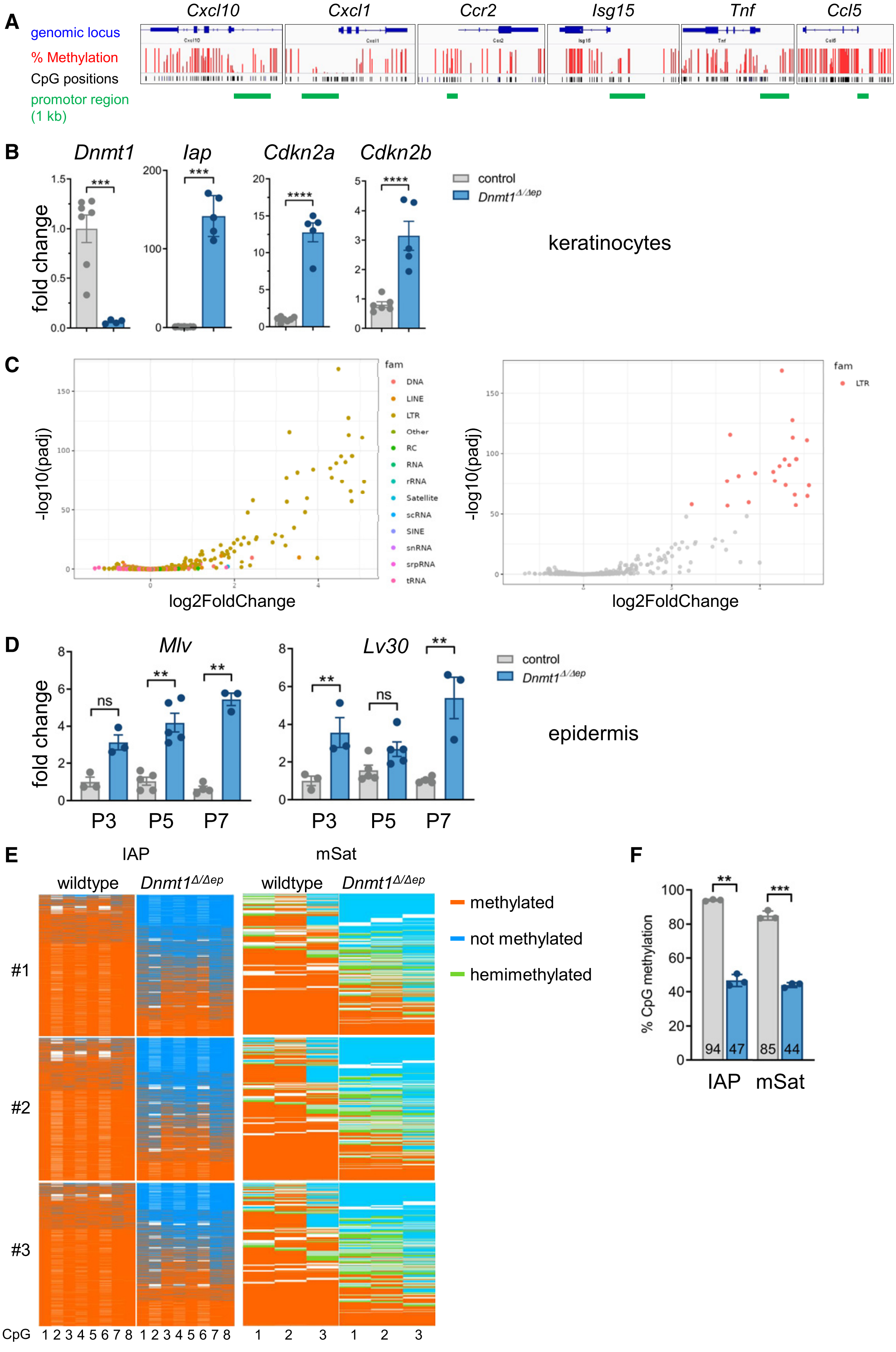
A
Cxcl10
Cxcl1
Ccr2
Isg15
Tnf
Ccl5
genomic locus
% Methylation
CpG positions
promotor region
(1 kb)
B
Dnmt1
Iap
Cdkn2a
Cdkn2b
fold change
control
Dnmt1Δ/Δep
keratinocytes
C
-log10(padj)
log2FoldChange
fam
DNA
LINE
LTR
Other
RC
RNA
rRNA
Satellite
scRNA
SINE
snRNA
srpRNA
tRNA
D
Mlv
Lv30
fold change
ns
P3
P5
P7
epidermis
E
IAP
mSat
wildtype
Dnmt1Δ/Δep
methylated
not methylated
hemimethylated
#1
#2
#3
CpG
F
% CpG methylation
94
47
85
44
IAP
mSat

Figure EV3.

Figure EV4. DNA hypomethylation results in defective mitosis and increase in H3S10ph-positive G2/M cells in suprabasal epidermal layers.

A, B Immunofluorescence double-labeling of G2/M cells (H3S10ph) and proliferating cells (Ki67) of dorsal skin sections from day 7 old control and *Dnmt1*$^{\Delta/\Delta ep}$ mice. Dashed lines indicate the dermal–epidermal border and arrows point to suprabasally located H3S10ph-positive nuclei. (B) Quantification of suprabasal H3S10ph-positive nuclei shown in (A) from day 7 old control (n = 4) and *Dnmt1*$^{\Delta/\Delta ep}$ mice (n = 4). Values were compared using two-tailed Student's *t*-test. Data are mean ± SEM. *$P \leq 0.05$.

C Quantification of basal (left) and suprabasal (right) cells from day 7 old control (n = 4) and *Dnmt1*$^{\Delta/\Delta ep}$ mice (n = 4) positive for H3S10ph and cells double positive for H3S10ph and Ki67 (as shown in A). Statistical analyses were done using two-tailed Student's *t*-test. Data are mean ± SEM. ns not significant, *$P \leq 0.05$.

D–F Effect of DAC treatment on expression of DNMTs and DNA methylation. (D) Cells were treated with 5-aza-2′-deoxycytidine (DAC) or vehicle (PBS) for 72 h, and protein extracts were analyzed for expression of DNMT1, DNMT3A, and DNMT3B. ß-Actin served as loading control. Signals were quantified relative to the ß-Actin signal and indicated as percentage of the respective PBS control value. (E) 5-meC quantification was performed by quantitative dot blot analysis. The PBS control value was set to 100%. The experiment was done in triplicates, and values were compared using two-tailed Student`s *t*-test. Data are mean ± SEM. *$P \leq 0.05$. (f) Representative images from aberrant mitotic and post-mitotic nuclei (H3S10ph-positive) observed in human keratinocytes treated with DAC. Arrows indicate chromosomal fragments and nucleoplasmic bridges.

G Human keratinocytes were treated with PBS (control), 1 μM DAC, or 5 μM DAC for 24 or 48 h and immunostained for H3S10ph and DNA (PicoGreen). Slides were randomized and analyzed in a blinded manner for mitotic and post-mitotic nuclear aberrations as shown in panel (F). About 100 H3S10ph-positive mitotic and post-mitotic nuclei per condition and replicate were analyzed for nuclear defects and calculated as percentage of the total number of H3S10ph-positive mitotic and post-mitotic nuclei. Statistical analyses were done using one-way ANOVA with post hoc Tukey multiple comparison test. Data are mean ± SEM. *$P \leq 0.05$, **$P \leq 0.01$, ***$P \leq 0.001$.

H Graphical description of the abnormal nuclei during each cell cycle phase. Data are mean ± SEM from 4 biological replicates per condition.

Data information: Scale bars, 10 μm (F), 100 μm (A).

 © 2021 The Authors

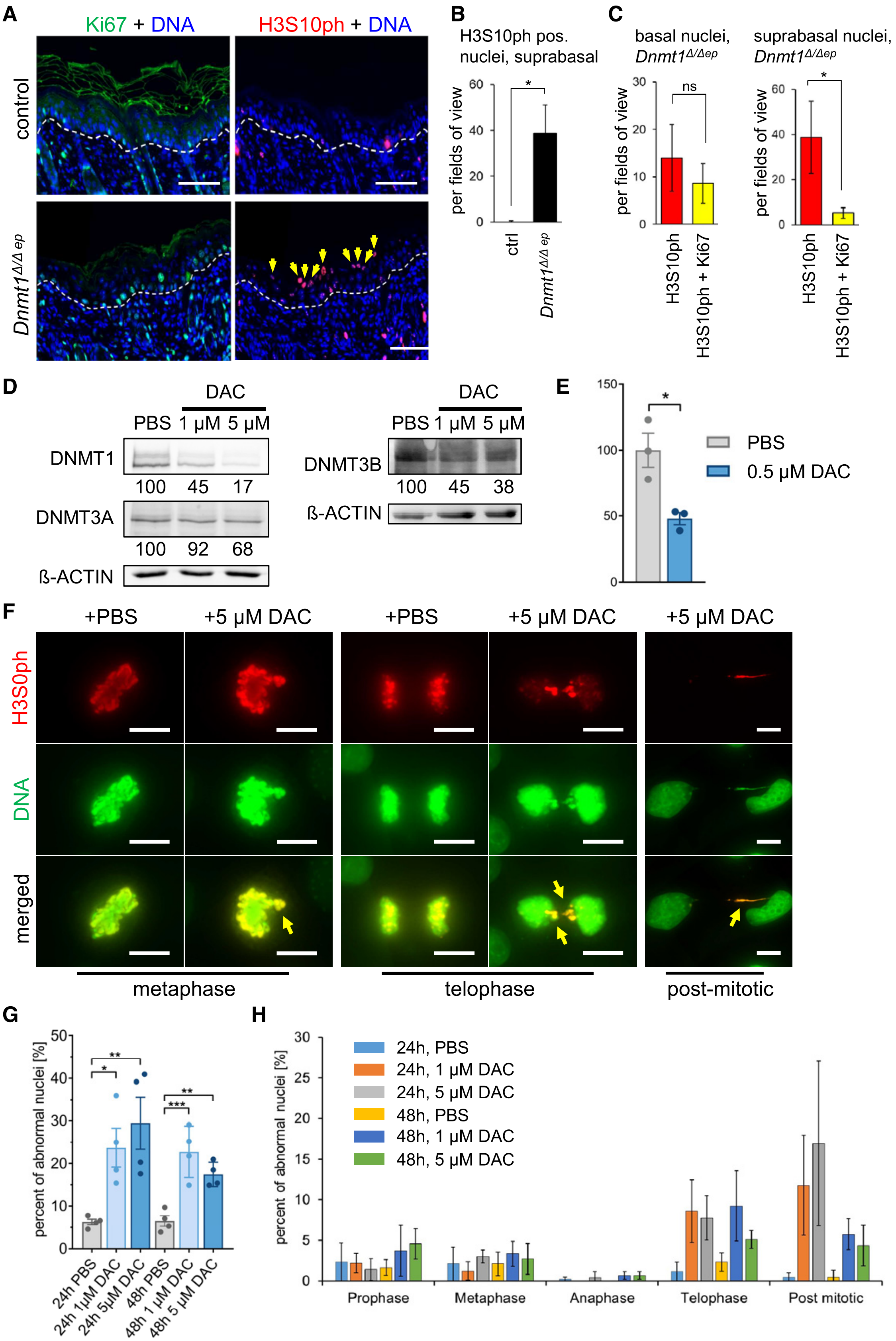
A
Ki67 + DNA
H3S10ph + DNA
control
Dnmt1Δ/Δ ep
B
H3S10ph pos. nuclei, suprabasal
per fields of view
ctrl
Dnmt1Δ/Δ ep
C
basal nuclei, Dnmt1Δ/Δep
suprabasal nuclei, Dnmt1Δ/Δep
ns
H3S10ph
H3S10ph + Ki67
D
DAC
PBS 1 μM 5 μM
DNMT1
100 45 17
DNMT3A
100 92 68
ß-ACTIN
DNMT3B
100 45 38
E
PBS
0.5 μM DAC
F
+PBS
+5 μM DAC
H3S0ph
DNA
merged
metaphase
telophase
post-mitotic
G
percent of abnormal nuclei [%]
24h PBS
24h 1μM DAC
24h 5μM DAC
48h PBS
48h 1 μM DAC
48h 5 μM DAC
H
24h, PBS
24h, 1 μM DAC
24h, 5 μM DAC
48h, PBS
48h, 1 μM DAC
48h, 5 μM DAC
Prophase
Metaphase
Anaphase
Telophase
Post mitotic

Figure EV4.

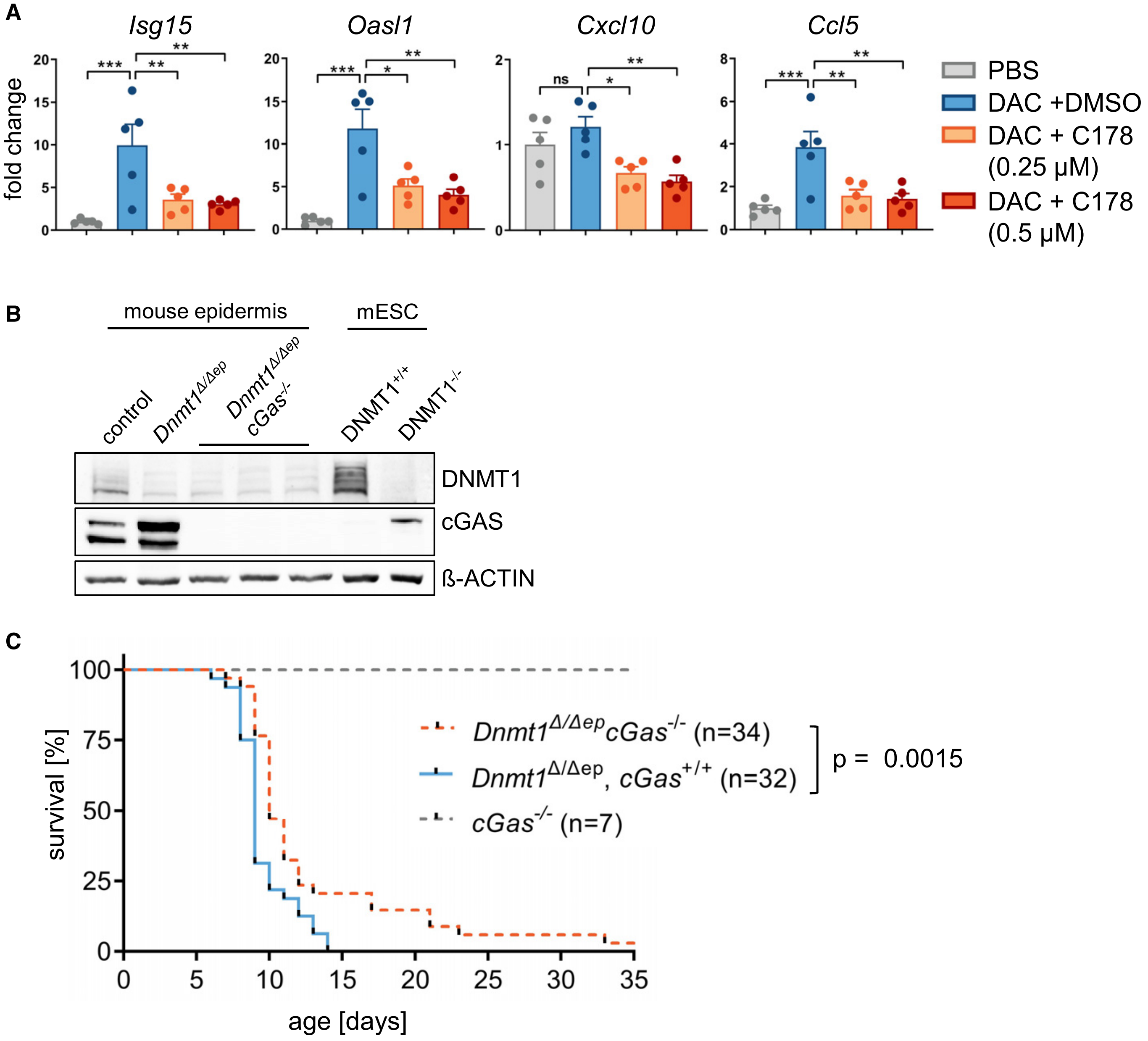

Figure EV5. Ablation of the cGAS/STING pathway results in decreased expression of immune genes and increased lifespan of *Dnmt1*$^{\Delta/\Delta ep}$ mice.

A Cultivated neonatal wild-type primary mouse keratinocytes were treated with PBS (control) or 2'-deoxy-5-azacytidine (DAC) for in total 72 h. After 48 h additionally, a specific STING inhibitor (C-178) or DMSO was added, and 24 h later, cells were harvested. Expression of immune-related genes was compared using one-way ANOVA with post hoc Tukey multiple comparison test. Data are mean ± SEM from 4 biological replicates per condition. ns, not significant; *$P \leq 0.05$, **$P \leq 0.01$, ***$P \leq 0.001$.

B Immunoblot analysis for expression of DNMT1 and cGAS in lysates of isolated mouse epidermis of control, *Dnmt1*$^{\Delta/\Delta ep}$, and *Dnmt1*$^{\Delta/\Delta ep}$ *Cgas*$^{-/-}$ mice. Additionally, mouse embryonic stem cells (mESC) with (DNMT1$^{-/-}$) or without (DNMT1$^{+/+}$) genetic deletion of *Dnmt1* were analyzed. Beta-actin was used as loading control.

C Kaplan–Meier plot of control (*Cgas*$^{-/-}$, $n = 7$), *Dnmt1*$^{\Delta/\Delta ep}$ *Cgas*$^{-/-}$ ($n = 32$), and *Dnmt1*$^{\Delta/\Delta ep}$ *Cgas*$^{-/-}$ ($n = 34$) mice using log-rank test (Mantel–Cox). $P < 0.0015$.

© 2021 The Authors
