# Supplementary material for: DNA hypomethylation leads to cGAS‐induced autoinflammation in the epidermis
Source: EMBO J. 2021 Sep 29;40(22):e108234. doi: 10.15252/embj.2021108234 (PMC8591534; doi:10.15252/embj.2021108234)
Supplement: Supplementary file 5 [file EMBJ-40-e108234-s002.pdf]

THE EMBO JOURNAL

DNA hypomethylation leads to cGAS-induced autoinflammation in the epidermis

Mirjam Beck, Heinz Fischer, Lisa Grabner, Tamara Groffics, Mircea Winter, Simone Tangermann, Tina Meischel, Barbara Zaussinger-Haas, Patrick Wagner, Carina Fischer, Christina Folie, Julia Arand, Christian Schoefer, Bernard Ramsahoye, Sabine Lagger, Georg Machat, Gregor Eisenwort, Stephanie Schneider, Alexandra Podhornik, Micheal Kothmayer, Ursula Reichart, Martin Glösmann, Ido Tamir, Michael Mildner, Raheleh Sheibani-Tezerji, Lukas Kenner, Peter Petzelbauer, Gerda Egger, Maria Sibilia, Andrea Ablasser, and Christian Seiser
DOI: 10.15252/embj.2021108234

Corresponding author(s): Christian Seiser (christian.seiser@meduniwien.ac.at)

Review Timeline:

Submission Date:	13th Mar 21
Editorial Decision:	28th Apr 21
Revision Received:	26th Jul 21
Editorial Decision:	24th Aug 21
Revision Received:	28th Aug 21
Accepted:	31st Aug 21

Editor: Karin Dumstrei

Transaction Report:

(Note: With the exception of the correction of typographical or spelling errors that could be a source of ambiguity, letters and reports are not edited. Depending on transfer agreements, referee reports obtained elsewhere may or may not be included in this compilation. Referee reports are anonymous unless the Referee chooses to sign their reports.)

1st Editorial Decision 28th Apr 2021

Dear Christian,

Thank you for submitting your manuscript to The EMBO Journal. I am sorry for the delay in getting back to you with a decision, but I have now received the input from three referees.

As you can see below, all three referees find the analysis interesting and insightful. They have only minor concerns that should be easy to resolve. I would therefore like you to invite a revised version. Let me know if we need to discuss any of the revisions further.

When preparing your letter of response to the referees' comments, please bear in mind that this will form part of the Review Process File, and will therefore be available online to the community. For more details on our Transparent Editorial Process, please visit our website: https://www.embopress.org/page/journal/14602075/authorguide#transparentprocess

Congratulations on a nice study!

with best wishes

Karin

Karin Dumstrei, PhD
Senior Editor
The EMBO Journal

Instructions for preparing your revised manuscript:

Please check that the title and abstract of the manuscript are brief, yet explicit, even to non-specialists.

When assembling figures, please refer to our figure preparation guideline in order to ensure proper formatting and readability in print as well as on screen:
https://bit.ly/EMBOPressFigurePreparationGuideline

IMPORTANT: When you send the revision we will require
- a point-by-point response to the referees' comments, with a detailed description of the changes made (as a word file).
- a word file of the manuscript text.
- individual production quality figure files (one file per figure)
- a complete author checklist, which you can download from our author guidelines (https://www.embopress.org/page/journal/14602075/authorguide).
- Expanded View files (replacing Supplementary Information)
Please see out instructions to authors
https://www.embopress.org/page/journal/14602075/authorguide#expandedview

Please remember: Digital image enhancement is acceptable practice, as long as it accurately

represents the original data and conforms to community standards. If a figure has been subjected to significant electronic manipulation, this must be noted in the figure legend or in the 'Materials and Methods' section. The editors reserve the right to request original versions of figures and the original images that were used to assemble the figure.

Further information is available in our Guide For Authors:
https://www.embopress.org/page/journal/14602075/authorguide

The revision must be submitted online within 90 days; please click on the link below to submit the revision online before 27th Jul 2021.

https://emboj.msubmit.net/cgi-bin/main.plex

Referee #1:

In this interesting manuscript from Beck et al. the authors investigate the downstream consequences of DNMT1 inactivation in epidermis. This is an interesting and well executed study. The manuscript is well written and easy to understand. The results are timely. Overall, I am very positive to this study and would like to congratulate the authors to a very exciting manuscript. I just have a few minor the comments that the authors may want to consider:

1. In Fig 1e DNA-methylation levels only decreased 60% as detected by HPLC. Why not more? Please explain in the text.

2. RNAseq: The incomplete loss of DNA methylation could perhaps explain the relatively low FC in the RNA seq data (Table S2)? It would be interesting to see a more detailed analysis of transposable elements from the RNAseq data in a scatter plot (including the visualization of TE families). Quite a low number of elements that are affected according to Table S2.

3. It would be valuable to investigate if the upregulated IAP-elements are reverse transcribed resulting in the generation of dsDNA-molecules.

4. I felt that the human in vitro experiments were very preliminary in nature. I think the manuscript would be better if the human part was removed.

5. Since the additional loss of cGAS not fully abolishes the inflammation, they speculate (page 12) that it could be other sensing pathways involved, such as TLR receptors sensing dsRNA, ssDNA, RNA/DNA hybrids. To their speculation, I think it would be fair to add that the presence of viral proteins (IAP-Gag) could also activate inflammation via TLRs.

6. The last paragraph in the discussion becomes a bit far-fetched speculation for me.

7. The knowledge of the various roles of DNA methylation has dramatically expanded the last decade due to technical advancements, yet the paper (Schubeler) cited in the introduction is 6 years old. Please include some recent references as well.

8. Fig1: In the upper left photo in 1f it is a bit unclear which pups belong to control or Dnmt□/□ep,

please indicate this better.

9. Please change the text accordingly:
DNMT1 - human protein
DNMT1 - human gene

Dnmt1 - mouse protein
Dnmt1 - mouse gene

10. Page 12: Sentence starting on second row "Mutations in DNMT3..." needs re-writing to fit into the following sentence.

Referee #2:

The manuscript by Beck et al. show that DNMT1 ablation in mouse epidermis induces a strong innate immune response with immune cell infiltration and disruption of epidermal homeostasis. The authors demonstrate that DNA hypomethylation led to genomic instability and DNA inflow into the cytosol. Cytosolic DNA activated the sensor and defense mechanism via cGAS, which the authors have evidence supporting an essential role using double knocked-out DNMT1 and cGAS mice. Double knock-out of DNMT1 and MAVS, which is a sensor for dsRNA, indicate that dsRNA was not the key cause for the induction of innate immunity via DNMT1 reduction. This manuscript is a well conducted study corroborating the role of DNMT1 for homeostasis in mouse epidermis. Although the authors also use DAC, which inhibits DNMT1 (and also DNMT3A/B), the study is a little too focused on the role of DNMT1. Broadening the view (and experiments) more into DNMT3A/B (not only in Fig. 4c) would have been better. The strength of this manuscript is that it helps to resolve the role of DNMT1 in mouse epidermis by correlating DNMT1 reduction with induced innate immunity and immune cell infiltration. The authors demonstrated a ~60% down-regulation of genomic DNA-methylation in epidermal cells after DNMT1/Dnmt1 knockout. Thus ~40% DNA-methylation remains. The manuscript is interesting to researchers in the general DNA methylation, cytosolic DNA and innate immunity field and specifically stimulates the research field of skin disease. The claims in this manuscript are strongly convincing, except the proposed non-involvement of Dnmt3A/B, which should be further analyzed and substantiated. The experiment with human keratinocytes in the presence of DAC is unfortunately not convincing. This experiment should be repeated with the Dnmt1 knockout cells in the presence of DAC. In addition, several other points should be taken into account:

Major points:
1) Overall the manuscript should mention the role and function of DNMT3A/B and the inhibition of DNMTs via DAC per se in the introduction more in detail (de novo and maintenance) and discussion (esp. the papers by McLaughlin et al. 2020 in PNAS about AZA and DNA-repair; and Li et al. 2016 about DNMT3A and HDAC9 and IFNb).
2) The authors analyzed mainly ISGs and also CXCL10 and TNF, but what about the first wave of innate immunity: IL1B/IL6/IL8 and IFNs? Esp. in regards to the inflammatory skin diseases, where IL and IFN play an essential role.
3) cGAS was analyzed and double-knocked out together with DNMT1. What about the other essential gene for cytosolic dsDNA: AIM2? AIM2 should be analyzed as well, esp. in regards to the published induction of this gene in inflammatory skin diseases (e.g. de Koning 2012, Exp. Dermatol)
4) The authors show a ~60% downregulation of genomic DNA-methylation in Dnmt1 knockout cells

(p. 5= DNMT1 or Dnmt1?). It would be important to treat Dnmt1-knockout cells with DAC in order to prove: A) if a ~100% downregulation of genomic DNA-methylation can be achieved; B) if DAC also targets Dnmt3A/B, which the authors claim is not occurring in human keratinocytes (p. 8), but the past literature does show DNMT3A/B inhibition with other cells. In summary, a similar experiment like in Fig. 4c, but with Dnmt1 knockout cells should be performed.
5) Fig. 4c: please quantify the DNMT1 and DNMT3A signals normalized to ß-actin. How long was the DAC treatment performed? Please include DNMT3B as well.

Minor points:
1) Please review the use of the gene names for mice and humans (Dnmt1 and DNMT1, respectively) throughout the text.
2) P. 12 sentence: Mutations in DNMT3B... is incomplete.
3) P.15: was the inhibitor C-178 purchased?
4) P. 18-19: write the primers in 5'-3' orientations.
5) P.21: Winter et al. 2103?

Referee #3:

Beck, Fisher and colleagues describe that loss of the DNA methyltransferase DNMT1 specifically in keratinocytes causes severe skin inflammation. Chronic DNA demethylation in keratinocytes in vivo results in DNA damage, loss of cell cycle progression and micronuclei formation. This results in the recognition of micronucleated DNA by the innate dsDNA sensor cGAS resulting in the development of a type I IFN response. This in itself is an important finding as it challenges previous studies in cancer cells showing that DNA demethylating drugs such as 5-Azacytidine drive an MDA5/MAVS-dependent type I IFN response through recognition of endogenous dsRNA molecules (PMIDs 26317465, 26317465 and 33087935).
Overall, I find the technical quality of the study by Beck, Fisher et al. very high and the conclusions originating from the data are very well supported by different experimental approaches. In my opinion, this study is worthy of publication in EMBO journal and I only have minor concerns, which are listed below.

1. In a follow up study from Roulois et al, Mehdipour et al. (PMID 33087935) find that intronic and intergenic SINEs rather than ERVs activate MDA5 upon inhibition of DNA methylation in cancer cells. Can the authors include these findings in the discussion?

2. Epidermis-specific DNMT1 mice crossed to cGAS knockout animals display reduced inflammation, but still die 11-12 days after birth. Can the authors comment on the possible cGAS/type I IFN-independent mechanisms that cause pathology and death in these animals?

3. Can the authors include a detailed protocol on how primary keratinocytes from Figure 2E were cultured? It is very hard to trace back the original protocol.

We would like to thank the reviewers for the positive comments and the constructive for the suggestions.

Referee #1:

1. In Fig 1e DNA-methylation levels only decreased 60% as detected by HPLC. Why not more? Please explain in the text.

The genome-wide quantitative HPLC data on DNA methylation showed that the overall DNA methylation was reduced by 60% in the epidermis of *Dnmt1*$^{\Delta/\Delta ep}$ mice in comparison to the control littermates. These results are in accordance with data obtained with the germline deletion of *Dnmt1* in mice. Dnmt1-deficient murine ES cells showed a 66% reduction in DNA methylation (Li *et al*, 1992), which is only slightly more than the decrease we observed in the conditional epidermis DNMT1 knockout pubs. The remaining DNA methylation levels are in part due to DNMT1 positive immune cells migrating into the epidermis (Fig. 2a) and potentially to compensating activities of DNMT3A and DNMT3B (Tsumura *et al*, 2006).
As requested by the reviewer we have added two sentences explaining these facts on page 5.

2. RNAseq: The incomplete loss of DNA methylation could perhaps explain the relatively low FC in the RNA seq data (Table S2)? It would be interesting to see a more detailed analysis of transposable elements from the RNAseq data in a scatter plot (including the visualization of TE families). Quite a low number of elements that are affected according to Table S2.

We thank the reviewer for this helpful suggestion. We have added two corresponding graphs showing that LTR retrotransposons are upregulated in *Dnmt1*-deficient keratinocytes as **Supplementary Fig.S5c**. The repeat counts table from repseq was annotated with the class/family name of the repeat and plotted in a scatter plot of log fold change versus log10(adjusted p-values).

3. *It would be valuable to investigate if the upregulated IAP-elements are reverse transcribed resulting in the generation of dsDNA-molecules.*

This is an interesting point. We tackled this question in two ways. We isolated cytosolic nucleic acids from wildtype and *Dnmt1*-deficient epidermis and performed quantitative PCR analysis for IAPs using cDNA prepared from RNA of *Dnmt1*-deficient epidermis as positive control. While IAP could be detected in up to 10,000-fold diluted cDNA we were unable to directly amplify IAP cDNA isolated from the cytosol of *Dnmt1*-deficient epidermal cells. In a second approach we used the S9.6 antibody previously used to detect cytosolic RNA:DNA duplexes generated by endogenous reverse transcriptase activity as autonomous inducers of skin inflammation (Moles *et al*, 2017) and performed IF experiments with epidermis

sections from wildtype and *Dnmt1*-deficient epidermis. We did not detect differences in cytosolic signals in these samples. However, we cannot exclude that our methods were not sensitive enough to detect minute amounts of IAP cDNA.

4. I felt that the human in vitro experiments were very preliminary in nature. I think the manuscript would be better if the human part was removed.

During the last years it became clear that epigenetic drugs have a sensitizing effect on immune checkpoint therapy. In particular, hypomethylating agents such as DAC are currently tested in combination with immune checkpoint blockade and show encouraging results (Daver *et al*, 2018). In addition, it has been shown that cancer-cell-intrinsic expression of cGAS determines tumor immunogenicity and makes tumors "hot" (Schadt *et al*, 2019). Therefore, our data might be indeed important for medical applications. Given that the regulation of the innate immune system is similar but not identical in mice and men we set out to test the effect of DNA hypomethylation in human cells. Finally the human keratinocyte system was also crucial for the response to the comments of Reviewer #2.

5. Since the additional loss of cGAS not fully abolishes the inflammation, they speculate (page 12) that it could be other sensing pathways involved, such as TLR receptors sensing dsRNA, ssDNA, RNA/DNA hybrids. To their speculation, I think it would be fair to add that the presence of viral proteins (IAP-Gag) could also activate inflammation via TLRs.

We fully agree. We have added this information to the relevant sentence in the Discussion on page 12.

6. The last paragraph in the discussion becomes a bit far-fetched speculation for me.

See response to Point 4.

7. The knowledge of the various roles of DNA methylation has dramatically expanded the last decade due to technical advancements, yet the paper (Schubeler) cited in the introduction is 6 years old. Please include some recent references as well.

As suggested by the reviewer we have added more recent references in the Introduction on pages 3 and 4.

8. Fig1: In the upper left photo in 1f it is a bit unclear which pups belong to control or Dnmt∆/∆ep, please indicate this better.

We added arrows to better indicate the respective genotypes.

9. Please change the text accordingly:

DNMT1 - human protein

DNMT1 - human gene

Dnmt1 - mouse protein

Dnmt1 - mouse gene

We have changed the organism-specific gene/protein nomenclature throughout the manuscript. However, murine protein symbols should be all upper case.
https://www.biosciencewriters.com/Guidelines-for-Formatting-Gene-and-Protein-Names.aspx

10. Page 12: Sentence starting on second row "Mutations in DNMT3..." needs re-writing to fit into the following sentence.

We have completed the sentence on page 12.

Referee #2:

Major points:

1) Overall the manuscript should mention the role and function of DNMT3A/B and the inhibition of DNMTs via DAC per se in the introduction more in detail (de novo and maintenance) and discussion (esp. the papers by McLaughlin et al. 2020 in PNAS about AZA and DNA-repair; and Li et al. 2016 about DNMT3A and HDAC9 and IFNb).

As suggested by the reviewer we have expanded the introduction with respect to the de novo methyltransferases DNMT3A/B and DAC effects (pages 3 and 4) and have included also the mentioned references in the Discussion on page 12.

2) The authors analyzed mainly ISGs and also CXCL10 and TNF, but what about the first wave of innate immunity: IL1B/IL6/IL8 and IFNs? Esp. in regards to the inflammatory skin diseases, where IL and IFN play an essential role.

We added data for IL1B, IL6, IFNa and IFNg to **Figure 2d**. IL1B, IL6 and IFNg show significant induction in the *Dnmt1*$^{\Delta/\Delta ep}$ epidermis, while IFNa remained unchanged.

3) cGAS was analyzed and double-knocked out together with DNMT1. What about the other essential gene for cytosolic dsDNA: AIM2? AIM2 should be analyzed as well, esp. in regards to the published induction of this gene in inflammatory skin diseases (e.g. de Koning 2012, Exp. Dermatol)

This is an important suggestion. We have analyzed AIM2 levels in the *Dnmt1*$^{\Delta/\Delta ep}$ and in the *Dnmt1*$^{\Delta/\Delta ep}$ and *Dnmt1*$^{\Delta/\Delta ep}$ *cGas*$^{-/-}$ epidermis and the corresponding controls. As shown in **Fig. 5e**, AIM2 expression was significantly induced in the absence of DNMT1. Interestingly, this induction was largely dependent on the presence of cGAS supporting the idea of a cross-talk between the cGAS and the inflammosome as previously described (Swanson *et al*, 2017).

4) *The authors show a ~60% downregulation of genomic DNA-methylation in Dnmt1 knockout cells (p. 5= DNMT1 or Dnmt1?). It would be important to treat Dnmt1-knockout cells with DAC in order to prove: A) if a ~100% downregulation of genomic DNA-methylation can be achieved; B) if DAC also targets Dnmt3A/B, which the authors claim is not occurring in human keratinocytes (p. 8), but the past literature does show DNMT3A/B inhibition with other cells. In summary, a similar experiment like in Fig. 4c, but with Dnmt1 knockout cells should be performed.*

5) Fig. 4c: please quantify the DNMT1 and DNMT3A signals normalized to ß-actin. How long was the DAC treatment performed? Please include DNMT3B as well.

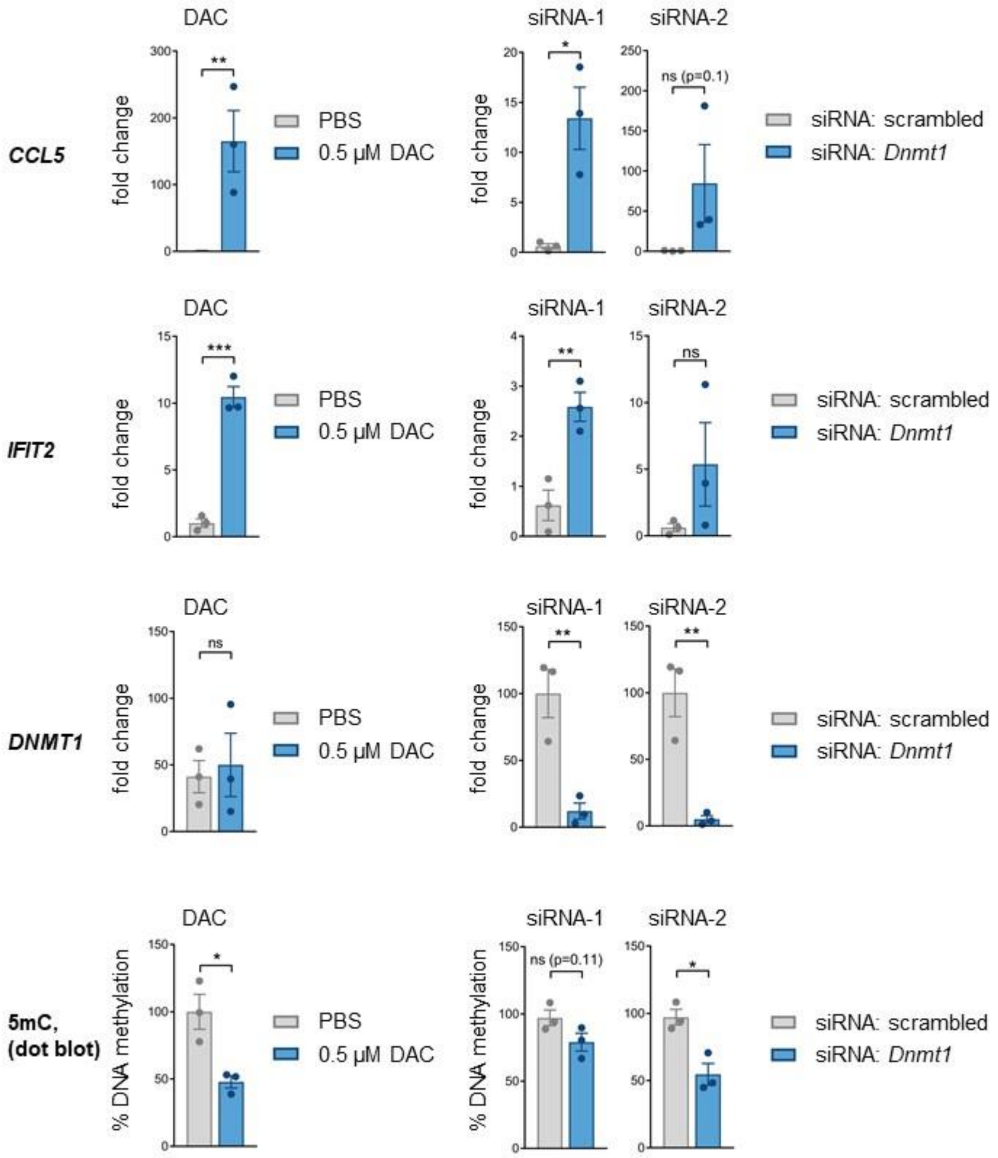

DAC, 5-aza-2′-deoxycytidine

FIGURE for reviewer 2

Mouse *Dnmt1* knockout keratinocytes do not tolerate DAC treatment and die within 48 hours. Therefore, we generated human *DNMT1* knock-down keratinocytes by using two different siRNAs. Both siRNAs caused a strong reduction in DNMT1 mRNA levels and a significant induction of immune genes such as *CCL5* and *IFIT2* (see Figure below). Again, DAC treatment resulted in loss of viability of *DNMT1* knock-down keratinocytes. The comparison of DAC-treated and knock-down keratinocytes gives more conclusive results: Depending on the siRNA DAC led to a stronger or

similar reduction in DNA methylation. Accordingly. DAC treatment gave a more robust induction of immune genes than the one of DNMT1 knock-down suggesting that DNMT3A/B also contribute to the effect on the innate immune system. In summary, the DAC treatment most likely affects the activities of all three DNMTs. We have added the data on DAC-induced DNA hypomethylation as **Supplementary Fig. S8e**.
The DAC treatment was for 72 hours as indicated now on page 9. We have included DNMT3B in the immunoblot figure shown now as **Supplementary Fig.S8d** and have quantified the signals. Interestingly, DNMT3A and DNMT3B also show reduced protein levels in response to DAC treatment suggesting again that DNMT3A/B could contribute to the effect of DAC in human keratinocytes. We have discussed this finding on page 9.

Minor points:
1) Please review the use of the gene names for mice and humans (Dnmt1 and DNMT1, respectively) throughout the text.

Done.

2) P. 12 sentence: Mutations in DNMT3B... is incomplete.

We have completed the sentence on page 12.

3) P.15: was the inhibitor C-178 purchased?

The inhibitor C-178 was provided by Andrea Ablasser. This is now mentioned in the section Materials and Methods on page 16.

4) P. 18-19: write the primers in 5'-3' orientations.

Primers are written in 5'-3' orientation.

5) P.21: Winter et al. 2103?

We have added the correct reference.

Referee #3:

1. In a follow up study from Roulois et al, Mehdipour et al. (PMID 33087935) find that intronic and intergenic SINEs rather than ERVs activate MDA5 upon inhibition of DNA methylation in cancer cells. Can the authors include these findings in the discussion?
We have included the findings of this paper in the Discussion on page 12 as suggested by the reviewer.

2. Epidermis-specific DNMT1 mice crossed to cGAS knockout animals display reduced inflammation, but still die 11-12 days after birth. Can the authors comment on the possible cGAS/type I IFN-independent mechanisms that cause pathology and death in these animals?

We have extended our discussion on cGAS/type I IFN-independent mechanisms on pages 9/10.

3. Can the authors include a detailed protocol on how primary keratinocytes from Figure 2E were cultured? It is very hard to trace back the original protocol.

We have added the requested information to the Materials and Methods section on page 15.

REFERENCES

Daver N, Boddu P, Garcia-Manero G, Yadav SS, Sharma P, Allison J, Kantarjian H (2018) Hypomethylating agents in combination with immune checkpoint inhibitors in acute myeloid leukemia and myelodysplastic syndromes. *Leukemia* 32: 1094-1105

Li E, Bestor TH, Jaenisch R (1992) Targeted mutation of the DNA methyltransferase gene results in embryonic lethality. *Cell* 69: 915-926

Moles JP, Griez A, Guilhou JJ, Girard C, Nagot N, Van de Perre P, Dujols P (2017) Cytosolic RNA:DNA Duplexes Generated by Endogenous Reverse Transcriptase Activity as Autonomous Inducers of Skin Inflammation in Psoriasis. *PloS one* 12: e0169879

Schadt L, Sparano C, Schweiger NA, Silina K, Cecconi V, Lucchiari G, Yagita H, Guggisberg E, Saba S, Nascakova Z *et al* (2019) Cancer-Cell-Intrinsic cGAS Expression Mediates Tumor Immunogenicity. *Cell reports* 29: 1236-1248 e1237

Swanson KV, Junkins RD, Kurkjian CJ, Holley-Guthrie E, Pendse AA, El Morabiti R, Petrucelli A, Barber GN, Benedict CA, Ting JP (2017) A noncanonical function of cGAMP in inflammasome priming and activation. *J Exp Med* 214: 3611-3626

Tsumura A, Hayakawa T, Kumaki Y, Takebayashi S, Sakaue M, Matsuoka C, Shimotohno K, Ishikawa F, Li E, Ueda HR *et al* (2006) Maintenance of self-renewal ability of mouse embryonic stem cells in the absence of DNA methyltransferases Dnmt1, Dnmt3a and Dnmt3b. *Genes Cells* 11: 805-814

1st Revision - Editorial Decision 24th Aug 2021

Dear Christian,

Thank you for submitting your revised manuscript to The EMBO Journal. Your study has now been seen by the original two referees #1 and 2. As you can see below, both referees appreciate the added changes and support publication here.

I am therefore very pleased to accept the manuscript for publication here. Before sending the formal acceptance letter there are just a few editorial things that have to be sorted out.

- The Austrian Science Fund P34998, P28705 are in the online submission system but not in the Acknowledgements

- Author contributions for Lisa Grabner, Simone Tangermann, Carina Fischer or Christina Folie (need differentiation), Julia Arand, Christian Schöfer, Michael Mildner, Lukas Kenner, Peter Petzelbauer, Andrea Ablasser are missing

- We need a COI

- The 4 appendix tables need to be compiled in 1 pdf with legends & ToC. Correct nomenclature is "Appendix Table S1" etc please also correct call out in text. The table legends need to be removed from manuscript.

- You have 9 expanded view figures but can only have 5. The other 4 can be added to the appendix and rename "Appendix Figure S1", etc. Add legends to the appendix file. The remaining 5 should be renamed "Figure EV1" etc. and have their legends in the manuscript after the main figure legends.

- There is a callout for Fig 4G but no such figure

- We include a synopsis of the paper (see http://emboj.embopress.org/). Please provide me with a general summary statement and 3-5 bullet points that capture the key findings of the paper.

- We also need a summary figure for the synopsis. The size should be 550 wide by [200-400] high (pixels).

- Our publisher has also done their pre-publication check on your manuscript. When you log into the manuscript submission system you will see the file "Data Edited Manuscript file". Please take a look at the word file and the comments regarding the figure legends and respond to the issues.

That should be all - let me know if you have any further questions.

With best wishes

Karin

Karin Dumstrei, PhD
Senior Editor
The EMBO Journal

Guide For Authors: https://www.embopress.org/page/journal/14602075/authorguide

The revision must be submitted online within 90 days; please click on the link below to submit the revision online before 22nd Nov 2021.

https://emboj.msubmit.net/cgi-bin/main.plex

Referee #1:

The authors addressed our queries and the new data and analysis supplied further supports their exciting hypothesis. A very nice paper that is a value to the field.

Referee #2:

This reviewer has no further comments. All suggestions were addressed accordingly.

2nd Revision - Editorial Decision 31st Aug 2021

Dear Christian,

Thank you for submitting your revised manuscript to The EMBO Journal. I have now had a chance to take a careful look at everything and all looks good!

Congratulations on a nice study

With best wishes

Karin

Karin Dumstrei, PhD
Senior Editor
The EMBO Journal

Please note that it is EMBO Journal policy for the transcript of the editorial process (containing referee reports and your response letter) to be published as an online supplement to each paper. If you do NOT want this, you will need to inform the Editorial Office via email immediately. More information is available here:
https://www.embopress.org/page/journal/14602075/authorguide#transparentprocess

Your manuscript will be processed for publication in the journal by EMBO Press. Manuscripts in the PDF and electronic editions of The EMBO Journal will be copy edited, and you will be provided with page proofs prior to publication. Please note that supplementary information is not included in the proofs.

Please note that you will be contacted by Wiley Author Services to complete licensing and payment information. The 'Page Charges Authorization Form' is available here:
https://www.embopress.org/pb-assets/embo-site/tej_apc.pdf

Should you be planning a Press Release on your article, please get in contact with embojournal@wiley.com as early as possible, in order to coordinate publication and release dates.

If you have any questions, please do not hesitate to call or email the Editorial Office. Thank you for your contribution to The EMBO Journal.

** Click here to be directed to your login page: https://emboj.msubmit.net

EMBO PRESS

YOU MUST COMPLETE ALL CELLS WITH A PINK BACKGROUND

PLEASE NOTE THAT THIS CHECKLIST WILL BE PUBLISHED ALONGSIDE YOUR PAPER

Corresponding Author Name: Christian Seiser

Journal Submitted to: EMBO Journal

Manuscript Number: EMBOJ-2021-108234

Reporting Checklist For Life Sciences Articles (Rev. June 2017)

This checklist is used to ensure good reporting standards and to improve the reproducibility of published results. These guidelines are consistent with the Principles and Guidelines for Reporting Preclinical Research issued by the NIH in 2014. Please follow the journal's authorship guidelines in preparing your manuscript.

A- Figures

1. Data

The data shown in figures should satisfy the following conditions:

- the data were obtained and processed according to the field's best practice and are presented to reflect the results of the experiments in an accurate and unbiased manner.
- figure panels include only data points, measurements or observations that can be compared to each other in a scientifically meaningful way.
- graphs include clearly labeled error bars for independent experiments and sample sizes. Unless justified, error bars should not be shown for technical replicates.
- if n< 5, the individual data points from each experiment should be plotted and any statistical test employed should be justified
- Source Data should be included to report the data underlying graphs. Please follow the guidelines set out in the author ship guidelines on Data Presentation.

2. Captions

Each figure caption should contain the following information, for each panel where they are relevant:

- a specification of the experimental system investigated (eg cell line, species name).
- the assay(s) and method(s) used to carry out the reported observations and measurements
- an explicit mention of the biological and chemical entity(ies) that are being measured.
- an explicit mention of the biological and chemical entity(ies) that are altered/varied/perturbed in a controlled manner.

- the exact sample size (n) for each experimental group/condition, given as a number, not a range;
- a description of the sample collection allowing the reader to understand whether the samples represent technical or biological replicates (including how many animals, litters, cultures, etc.).
- a statement of how many times the experiment shown was independently replicated in the laboratory.
- definitions of statistical methods and measures:
  - common tests, such as t-test (please specify whether paired vs. unpaired), simple χ2 tests, Wilcoxon and Mann-Whitney tests, can be unambiguously identified by name only, but more complex techniques should be described in the methods section;
  - are tests one-sided or two-sided?
  - are there adjustments for multiple comparisons?
  - exact statistical test results, e.g., P values = x but not P values < x;
  - definition of 'center values' as median or average;
  - definition of error bars as s.d. or s.e.m.

Any descriptions too long for the figure legend should be included in the methods section and/or with the source data.

In the pink boxes below, please ensure that the answers to the following questions are reported in the manuscript itself. Every question should be answered. If the question is not relevant to your research, please write NA (non applicable). We encourage you to include a specific subsection in the methods section for statistics, reagents, animal models and human subjects.

USEFUL LINKS FOR COMPLETING THIS FORM

http://www.antibodypedia.com
http://1degreebio.org
http://www.equator-network.org/reporting-guidelines/improving-bioscience-research-repor
http://grants.nih.gov/grants/olaw/olaw.htm
http://www.mrc.ac.uk/Ourresearch/Ethicsresearchguidance/Useofanimals/index.htm
http://ClinicalTrials.gov
http://www.consort-statement.org
http://www.consort-statement.org/checklists/view/32-consort/66-title
http://www.equator-network.org/reporting-guidelines/reporting-recommendations-for-tumo
http://datadryad.org
http://figshare.com
http://www.ncbi.nlm.nih.gov/gap
http://www.ebi.ac.uk/ega
http://biomodels.net/
http://biomodels.net/miriam/
http://jjj.biochem.sun.ac.za
https://osp.od.nih.gov/biosafety-biosecurity-and-emerging-biotechnology/
http://www.selectagents.gov/

B- Statistics and general methods

Please fill out these boxes (Do not worry if you cannot see all your text once you press return)

1.a. How was the sample size chosen to ensure adequate power to detect a pre-specified effect size?	Mouse experiments were at least conducted twice with at least three individuals per genotype of the same litter. If the litter did not provide three control and three knockout individuals, mice of different litters of the same age were used for isolate biological material. The material of different litters was futher processed together.
1.b. For animal studies, include a statement about sample size estimate even if no statistical methods were used.	For mouse experiments, processing of tissue samples was conducted from at least three mice per genotype, which was then conducted on at least two separate occassions.
2. Describe inclusion/exclusion criteria if samples or animals were excluded from the analysis. Were the criteria pre-established?	For qRT-PCR of epidermal mouse samples (Fig. 3c, 5e) Grubbs-Outlyer Test was conducted (https://www.graphpad.com/quickcalcs/Grubbs1.cfm), and samples that were identified as significant outlyer were removed (Significance level <0.05, two sided)
3. Were any steps taken to minimize the effects of subjective bias when allocating animals/samples to treatment (e.g. randomization procedure)? If yes, please describe.	NA
For animal studies, include a statement about randomization even if no randomization was used.	In our experiments we compared control mice with knockout mice within one litter, randomization of these animals was not required.
4.a. Were any steps taken to minimize the effects of subjective bias during group allocation or/and when assessing results (e.g. blinding of the investigator)? If yes please describe.	For experiments in which data were collected manually (histo score: Fig. 5b, EV 6c , quantification of micronuclei: Fig 4d), samples previously blinded and randomized
4.b. For animal studies, include a statement about blinding even if no blinding was done	Scanned histology pictures were analyzed by certified pathologist in a blinded manner.
5. For every figure, are statistical tests justified as appropriate?	yes
Do the data meet the assumptions of the tests (e.g., normal distribution)? Describe any methods used to assess it.	yes, normal distribution
Is there an estimate of variation within each group of data?	yes

Is the variance similar between the groups that are being statistically compared?	yes

C- Reagents

6. To show that antibodies were profiled for use in the system under study (assay and species), provide a citation, catalog number and/or clone number, supplementary information or reference to an antibody validation profile. e.g., Antibodypedia (see link list at top right), 1DegreeBio (see link list at top right).	Primary antibodies for immunoblotting: DNMT1 (sc-20701, Santa Cruz Biotechnology), DNMT3a (sc-20703, Santa Cruz Biotechnology), DNMT3b (sc-376043, Santa Cruz Biotechnology) beta-Actin (A5316, Sigma), Lamin B (sc-6216, Santa Cruz Biotechnology), H3 C-terminal (ab1791, Abcam), IAP (DOI:10.1093/nar/gkj416, kindly provided by the Bryan R. Cullen lab), PCNA (C1 PC10, Dako), gammaH2A.X S139ph (ab2893, Abcam), cGas (31659, Cell Signaling). Primary antibodies used for IHC: Ki67 (Novocastra), Keratin 10, Keratin 6, Loricrin (Covance), p53 (NCL-p53-CM5p, Novocastra), cleaved-caspase 3 (CC3, cs-96615, Cell Signaling), PCNA (DAKO), DNMT1 (Abcam, ab188453), CGAS (Cell Signaling, 31659; Cell Signaling 15102) 5mC (Diagenode 006-500), CD45 (Abcam ab10558), histone H3S10ph (Santa Cruz Biotechnology SC-8656-R). Primary antibodies used for FACS: CD45 APC-Cy7 (Biolegend, clone 30-F11), MHC-II PE (Biolegend, clone M5), CD11b PE-Cy7 (Biolegend, clone M1/70), F4/80 APC (eBioscience, clone BM8), Ly6G PE (Biolegend), 7-AAD PerCP (Sigma).
7. Identify the source of cell lines and report if they were recently authenticated (e.g., by STR profiling) and tested for mycoplasma contamination.	Immortalized human epidermal keratinocytes NHEK/SVTERT3-5 (DOI:10.1038/s41598-018-31829-0) were provided by EVERCYTE, Vienna, Austria, (CLHT-011-0026-5). Cell line is from bacterial (incl. mycoplasma) and fungal contaminations (https://www.evercyte.com/nhek-svtert3-23.html)

* for all hyperlinks, please see the table at the top right of the document

D- Animal Models

8. Report species, strain, gender, age of animals and genetic modification status where applicable. Please detail housing and husbandry conditions and the source of animals.	All mouse lines were bred to a mixed genetic background of C57BL/ 6J 129SV. K5-Cre+ Dnmt1f/f (Dnmt1Δ/Δep) mice were generated in our laboratory. Mavs-/- and Cgas-/- mice were obtained from Jackson laboratory. Breeding and housing of control and mutant animals was performed at the animal facility of the Medical University of Vienna. The animal facility is equipped with a time switch (12 hours day/night cycles). The individually ventilated cages are connected to the Facility Management of the Medical University of Vienna). Mice are fed with autoclaved fortified mouse pellets (company SSNIFF) and drinking water. Food and water supply will be monitored daily and recorded.
9. For experiments involving live vertebrates, include a statement of compliance with ethical regulations and identify the committee(s) approving the experiments.	All animal experiments were performed in accordance with a protocol authorized by the Austrian Ministry of Science and Research, following the approval by the National Ethical Committee for Animal Experimentation. The Austrian Ministry for Science and Research approved the corresponding proposals GZ BMWFW-66.009/0138-WF/V/3b/2017 and GZ BMWFW-66.009/0276-WF/V/3b/2017.
10. We recommend consulting the ARRIVE guidelines (see link list at top right) (PLoS Biol. 8(6), e1000412, 2010) to ensure that other relevant aspects of animal studies are adequately reported. See author guidelines, under 'Reporting Guidelines'. See also: NIH (see link list at top right) and MRC (see link list at top right) recommendations. Please confirm compliance.	We confirm the compliance of these guidelines.

E- Human Subjects

11. Identify the committee(s) approving the study protocol.	no human subjects
12. Include a statement confirming that informed consent was obtained from all subjects and that the experiments conformed to the principles set out in the WMA Declaration of Helsinki and the Department of Health and Human Services Belmont Report.	NA
13. For publication of patient photos, include a statement confirming that consent to publish was obtained.	NA
14. Report any restrictions on the availability (and/or on the use) of human data or samples.	NA
15. Report the clinical trial registration number (at ClinicalTrials.gov or equivalent), where applicable.	NA
16. For phase II and III randomized controlled trials, please refer to the CONSORT flow diagram (see link list at top right) and submit the CONSORT checklist (see link list at top right) with your submission. See author guidelines, under 'Reporting Guidelines'. Please confirm you have submitted this list.	NA
17. For tumor marker prognostic studies, we recommend that you follow the REMARK reporting guidelines (see link list at top right). See author guidelines, under 'Reporting Guidelines'. Please confirm you have followed these guidelines.	NA

F- Data Accessibility

18: Provide a "Data Availability" section at the end of the Materials & Methods, listing the accession codes for data generated in this study and deposited in a public database (e.g. RNA-Seq data: Gene Expression Omnibus GSE39462, Proteomics data: PRIDE PXD000208 etc.) Please refer to our author guidelines for 'Data Deposition'.<br><br>Data deposition in a public repository is mandatory for:<br>a. Protein, DNA and RNA sequences<br>b. Macromolecular structures<br>c. Crystallographic data for small molecules<br>d. Functional genomics data<br>e. Proteomics and molecular interactions	Data availability<br>The RNA-seq data sets have been deposited to the NCBI Gene Expression Omnibus (GEO) under GSE166356 with the sample ids 34189 – 34194.
19. Deposition is strongly recommended for any datasets that are central and integral to the study; please consider the journal's data policy. If no structured public repository exists for a given data type, we encourage the provision of datasets in the manuscript as a Supplementary Document (see author guidelines under 'Expanded View' or in unstructured repositories such as Dryad (see link list at top right) or Figshare (see link list at top right).	
20. Access to human clinical and genomic datasets should be provided with as few restrictions as possible while respecting ethical obligations to the patients and relevant medical and legal issues. If practically possible and compatible with the individual consent agreement used in the study, such data should be deposited in one of the major public access-controlled repositories such as dbGAP (see link list at top right) or EGA (see link list at top right).	
21. Computational models that are central and integral to a study should be shared without restrictions and provided in a machine-readable form. The relevant accession numbers or links should be provided. When possible, standardized format (SBML, CellML) should be used instead of scripts (e.g. MATLAB). Authors are strongly encouraged to follow the MIRIAM guidelines (see link list at top right) and deposit their model in a public database such as Biomodels (see link list at top right) or JWS Online (see link list at top right). If computer source code is provided with the paper, it should be deposited in a public repository or included in supplementary information.	

G- Dual use research of concern

22. Could your study fall under dual use research restrictions? Please check biosecurity documents (see link list at top right) and list of select agents and toxins (APHIS/CDC) (see link list at top right). According to our biosecurity guidelines, provide a statement only if it could.	no
